# Modular synthesis of zinc(ii)-bis(triazole) recognition sites for the conformational control of foldamers

**DOI:** 10.1039/d5ob01226k

**Published:** 2025-08-28

**Authors:** Flavio della Sala, Benedicte Doerner, Simon J. Webb

**Affiliations:** a Department of Chemistry, University of Manchester Oxford Road Manchester M13 9PL UK S.Webb@manchester.ac.uk; b Manchester Institute of Biotechnology, University of Manchester 131 Princess Street Manchester M1 7DN UK

## Abstract

Zinc(ii) bis(triazolyl)(pyridyl)amine (Zn(BTPA)) complexes on the end of α-amino-iso-butyric acid (Aib) foldamers are able to transfer chirality from bound anions to the helical foldamer body. Zn(BTPA) could be obtained by simple synthetic methodology that allowed a range of functional groups to be installed around the binding site, exemplified with a fluorophore, a macrocyclic bridge and Aib itself. Changing functional group did not prevent chiral ligands from controlling foldamer conformation, although differences in complexation kinetics and equilibria were observed. Addition of acetate gave a 2 : 1 foldamer : acetate intermediate at sub-stoichiometric acetate; a similar intermediate was implied during titration with Boc-Pro. A bulkier phosphate ligand or a more sterically hindered site did not form similar intermediates. The modular construction of Zn(BTPA)-capped foldamers will allow these conformational relays to be installed in a wide range of biomimetic constructs.

## Abstract 2

Zinc(ii) bis(triazole)pyridyl complexes are able to mediate chirality transfer from bound anions to the body of a 3_10_-helical foldamer. Simple synthetic methodology allows a range of functional groups to be installed around the binding site.

## Graphical abstract

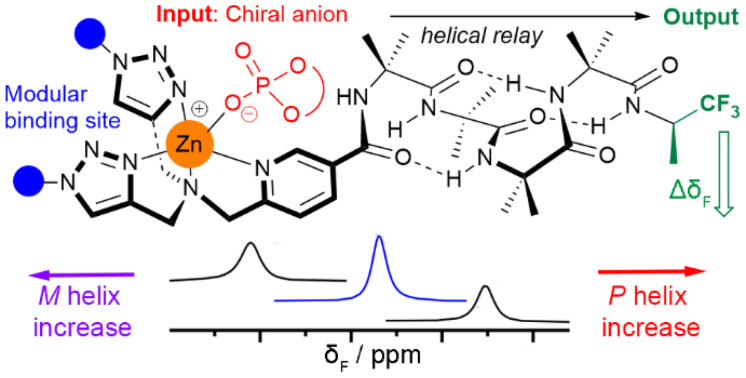

## Introduction

Metal ions play important roles in enzyme active sites and during ligand binding to proteins. Zinc(ii) for example can control protein shape by coordinating to side chains on different secondary structures, as found in the zinc finger motif. The resulting structurally defined regions are critical for protein–DNA binding.^1^ Metal ions in proteins can also directly bind to cognate ligands, *e.g.* some calcium(ii)-dependent animal lectins form direct coordination links between the sugar hydroxyls and bound calcium(ii).^2^ Ligand binding to metal ions in proteins can then induce global conformational changes, with the binding of oxygen to haemoglobin one of the best known examples.^3^ Protein folding into geometrically defined pockets around the metal ions are important for these proteins to function.

Folded oligomers (foldamers) can coordinate to metal ions,^4,5^ with some foldamers shown to mimic metalloprotein structure, including the zinc finger motif.^6,7^ The metal ions can also become catalytic centres on the foldamers and/or provide locations for ligand binding. Ligand-induced conformational change, as observed in haemoglobin, can also be replicated. Upon binding, some ligands will perturb the conformational landscape of dynamic foldamers,^8–11^ which are a type of foldamer that undergo rapid conformational interchange. α-Amino-iso-butyric acid (Aib) foldamers are rod-like dynamic foldamers that can undergo rapid long range (>1 nm) conformational change in response to external stimuli. Their chief conformational populations are 3_10_ helices that have either a right-handed (*P*) or left-handed (*M*) screw-sense. Ligand binding at one terminus can cause these Aib foldamers to undergo end-to-end conformational change, which changes the proportion of *P* to *M* helices. These changes can be expressed as the helical excess, h.e., which is the fractional excess of *P* helix over *M* helix (h.e. = ([*P*] − [*M*])/([*P*] + [*M*])); this can be calculated from representative NMR spectra.^12^ This simple *P vs. M* conformational landscape has led to Aib foldamers being used to mimic aspects of biological signal transduction, particularly how ligand recognition can initiate conformational change across multi-nanometre distances. To mediate ligand recognition, Zn(ii) and Cu(ii) complexes can be placed at one end of the Aib foldamers.^9,13–15^ Like related complexes in the literature,^16–21^ they bind chiral anions, including carboxylates.^9*a*^

**Fig. 1:**
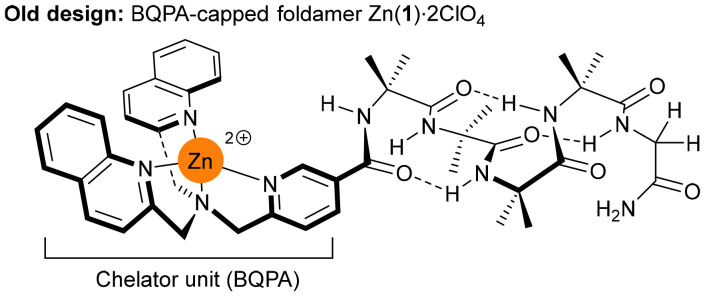
Reported foldamer Zn(1)·2ClO_4_ bearing the bis(quinolyl)(pyridyl)amine (BQPA) binding pocket.^15^ Perchlorate anions omitted for clarity.

The M(ii)-bis(quinolyl)(pyridyl)amine (BQPA, Fig. 1) binding site is the best to date for turning ligand chirality into a change in the *P* : *M* screw-sense ratio of an Aib foldamer. Its effectiveness has been ascribed to the steric bulk of the quinolyl arms and the “propeller” conformation they adopt.^22^ Both Zn(ii) and Cu(ii) complexes are effective. Foldamers capped with Zn(ii)-BQPA could sense the enantiomeric excess (ee) of scalemic mixtures of chiral carboxylates,^22^ due to rapid ligand exchange at the Zn(ii) site.^15^ A Cu(ii)-(BQPA) recognition site provided a synthetic receptor that responded to chiral carboxylates (the input signal) by undergoing a conformational change either in solution or deep into lipid bilayers.^9*a*^

These previous Aib foldamers with BQPA have a metal ion-chelating pocket that is symmetric and unfunctionalised,^13–15^ unlike the naturally asymmetric binding pockets of proteins. We wished to retain the desirable recognition characteristics of BQPA but add functionality to the “arms” around the binding site. BQPA itself was difficult to modify and its relatively poor stability also required it to be added last during synthesis.

**Fig. 2:**
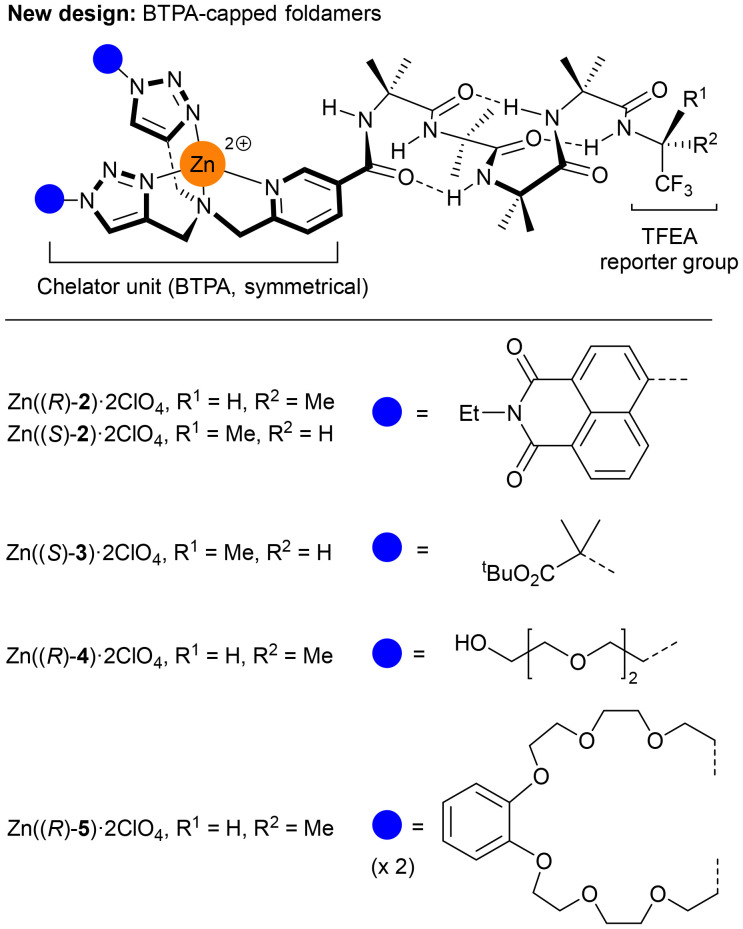
Foldamers Zn(2–5)·2ClO_4_ bearing the bis(triazolyl)(pyridyl)amine (BTPA) binding pocket, which is constructed in a modular fashion from different azides. Perchlorate anions are omitted for clarity.

**Fig. 3:**
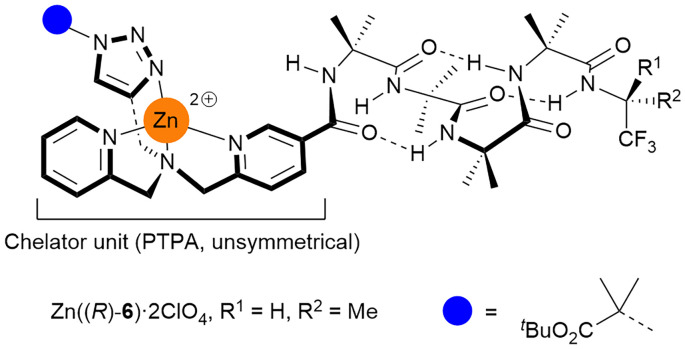
Foldamer Zn((*R*)-6)·2ClO_4_ bearing the (pyridyl)(triazolyl)(pyridyl) (PTPA) binding pocket with different binding arms. Perchlorate anions are omitted for clarity.

To better replicate metal ion containing binding pockets in proteins, simpler methods were needed to introduce functionality and decrease symmetry. To this end, we have explored the use of copper-catalysed alkyne–azide cycloaddition (CuAAC) reactions to create metal ion binding pockets^23,24^ (Fig. 2 and 3) that are flanked by selected substituents. The use of CuAAC allows simple modification of the synthetic route to provide either symmetrical or unsymmetrical metal ion binding pockets.

**Fig. 4:**
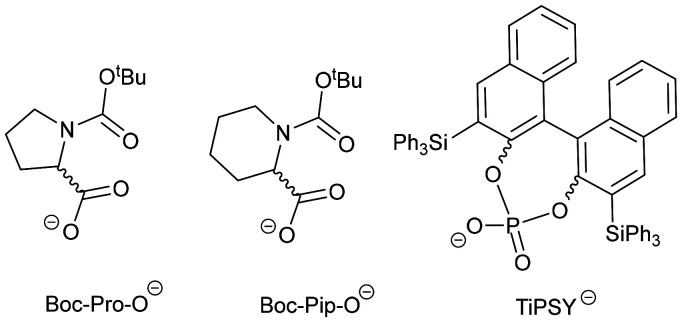
Chiral anionic ligands for zinc(ii). Boc-Pro-O^−^, Boc-Pip-O^−^ and TiPSY^−^ are derived from *N*-(*tert*-butoxycarbonyl)-proline, *N*-(*tert*-butoxycarbonyl)-pipecolinic acid and 3,3′-bis(triphenylsilyl)-1,1′-binaphthyl-2,2′-diyl hydrogenphosphate respectively.

To study the effect of these binding sites at the N-terminus of Aib foldamers, we placed the recently reported (*R*)-1-(trifluoromethyl)-ethylamido ((*R*)-TFEA) reporter group at the C-terminus.^25^ This group provides ^19^F NMR spectroscopic reports on changes in the conformational populations of Aib foldamers that are induced by chiral anionic ligands binding to the Zn(ii) (such as carboxylates or phosphates, Fig. 4).^26^

## Results and discussion

### Synthesis

**Scheme 1:**
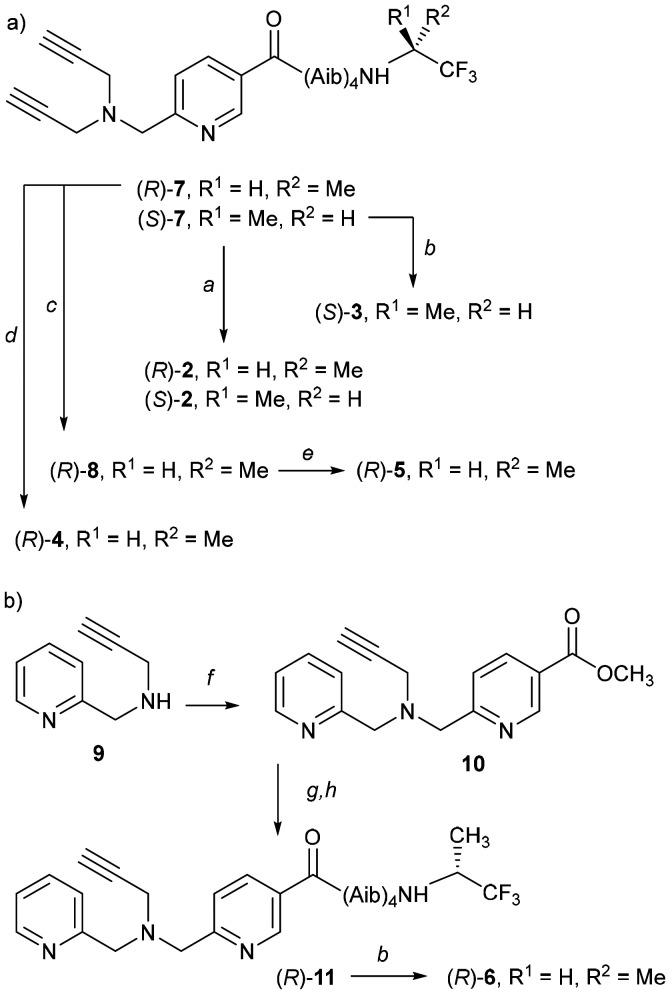
(a) Synthesis of foldamers 2–5. Reagents: *a*. 6-Azido-2-ethyl-1*H*-benzo[*de*]isoquinoline-1,3(2*H*)-dione, CuSO_4_·5H_2_O, sodium ascorbate, DMF, rt.^26^*b*. N_3_AibO^*t*^Bu,^13^ CuSO_4_·5H_2_O, sodium ascorbate, DMF, rt. *c*. 8-Azido-3,6-dioxaoctyl mesylate S18, CuSO_4_·5H_2_O, sodium ascorbate, DMF, rt. *d*. 8-Azido-3,6-dioxaoctanol, CuSO_4_·5H_2_O, sodium ascorbate, DMF, rt. *e*. Catechol, Cs_2_CO_3_, CH_3_CN, reflux. (b) Synthesis of foldamers 6, 11. Reagents: *f*. Methyl 6-(bromomethyl)nicotinate S15, DIPEA, CH_3_CN, rt. *g*. KOH, CH_3_OH, reflux. *h*. (Aib)_4_((*R*)-TFEA), EDC·HCl, HOBt, Et_3_N, CH_3_CN, rt.

Fluorinated motifs have great utility for both controlling the helical screw-sense in Aib foldamers and reporting on the helical excess of Aib foldamers.^8,27–29^ The TFEA ^19^F NMR reporter group allows the determination of the helical excess induced at the N-terminus of Aib foldamers (h.e._0_) both in organic solvents, micelles and when embedded in phospholipid vesicles.^25,26^ The robustness of TFEA towards synthetic conditions made it attractive for the development of new binding pockets. Either the (*R*)- or the (*S*)-TFEA reporter were conjugated to the readily accessible foldamer N_3_(Aib)_4_OH, then azide hydrogenation and elaboration at the N-terminus gave key bis-alkyne precursors (*R*)-7 and (*S*)-7 (Scheme 1). CuAAC reactions on these precursors had been shown to give BTPA-capped fluorescent foldamers (*R*)-2 and (*S*)-2.^26^ Applying the same CuAAC procedure but using N_3_AibO^*t*^Bu,^13^ 8-azido-3,6-dioxaoctanol or 8-azido-3,6-dioxaoctyl mesylate gave the other *N*-functionalised BTPA foldamers (*S*)-3, (*R*)-4 and (*R*)-8 in good yield (see SI, Section S2). Foldamer (*R*)-8 in turn gave access to (*R*)-5, which has a catechol/oligo(ethyleneglycol) bridge that was created through a Cs^+^ templated S_N_2 reaction. This oligoether bridge is close to the tetrapodal Zn(ii) chelating site and might enhance or otherwise alter ligand recognition at Zn(ii). It could also permit the introduction of rotaxanated structures.

Installing a monoalkyne in the place of the dialkyne in 7 can give unsymmetrical metal ion binding sites (Fig. 3). The (pyridyl)(triazolyl)(pyridyl)amine (PTPA) moiety of (*R*)-6 was accessed by reductive amination of amine 9.^30^ Mono-alkyne precursor 10 was hydrolysed then coupled to NH_2_(Aib)_4_((*R*)-TFEA). Finally a CuAAC reaction with N_3_AibO^*t*^Bu^13^ provided PTPA-capped foldamer (*R*)-6.

### Addition of zinc(ii) perchlorate

**Fig. 5:**
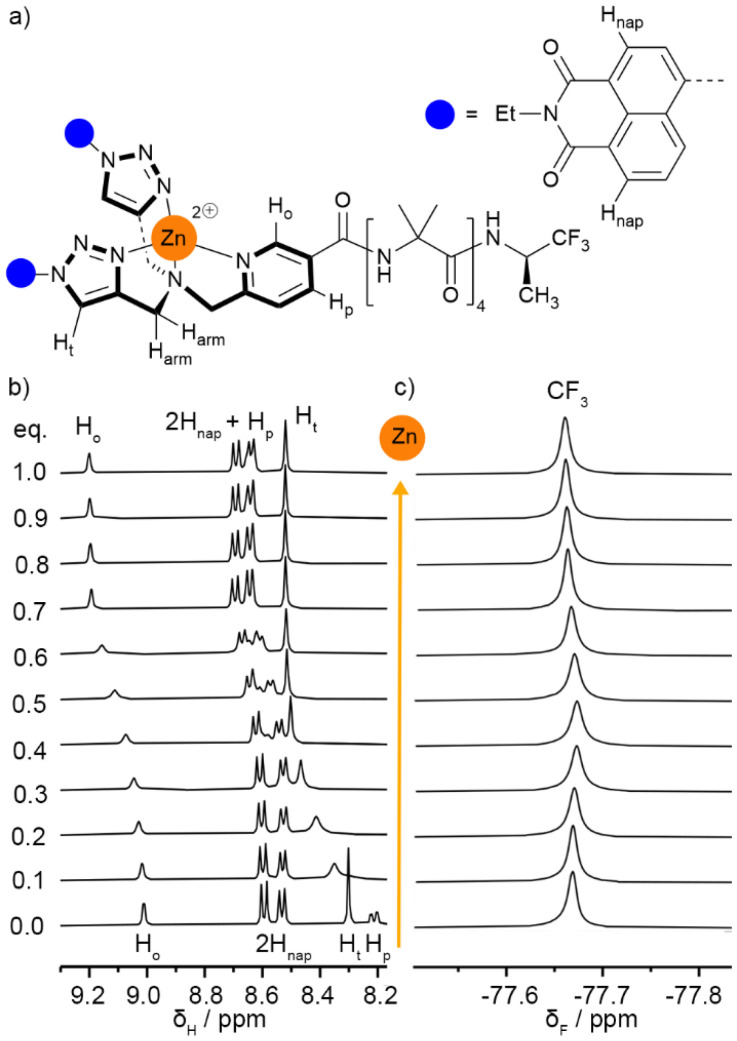
Addition of one equivalent of zinc(ii) perchlorate to foldamer (*R*)-2. (a) Labelling of selected protons around the Zn(ii) ion. (b) Partial stacked ^1^H NMR spectra (CD_3_CN, 400 MHz, 298 K). (c) Partial stacked ^19^F NMR spectra (CD_3_CN, 376 MHz, 298 K); spectra referenced with C_6_F_6_ at −164.38 ppm.^33^

Foldamer 2 was chosen to exemplify complexation of Zn(ii) by the BTPA group. Foldamer (*R*)-2 was titrated with zinc perchlorate in CD_3_CN (Fig. 5).^31^^1^H NMR spectroscopy showed a gradual, generally downfield, shift of aromatic peaks over the course of the titration, consistent with fast exchange between free and complexed foldamers at substoichiometric ratios of Zn(ii). Concurrent resonance broadening was also initially observed, before sharpening and a decrease of chemical shift movement at *ca.* 0.7 eq. of zinc. After complexation, significant downfield shifts were observed for the *ortho*-pyridyl (H_o_), *para*-pyridyl (H_p_), and triazole (H_t_) proton resonances (Δ*δ* = 187, 428 and 218 ppb respectively, Fig. 5 and Fig. S1 in the SI), shifts that are consistent with coordination to Zn(ii).^32^ Another diagnostic change was a downfield shift (280 ppb) and splitting of the triazole-methylene (H_arm_) protons from a singlet to four doublets (Fig. S1 in the SI), consistent with Zn(ii) complexation stopping tertiary amine inversion and making the arms inequivalent with diastereotopic methylenes. ^19^F NMR spectroscopy showed that Zn(ii) complexation at the N-terminus gave only a very small +8 ppb downfield shift for the C-terminal CF_3_ resonance of the reporter group (Fig. 5c), indicating little involvement with the newly installed Zn(ii).

Similar changes were observed for (*S*)-3, (*R*)-4 and (*R*)-5 (see SI, Section S3). However, the ^1^H NMR spectrum of Zn((*R*)-5)·2ClO_4_ (see the SI, Fig. S4) showed an increase in the number and broadness of peaks from the crown-ether protons, suggesting additional conformational states for the macrocycle after Zn(ii) addition. Interestingly, the catechol protons were downfield shifted (Δ*δ ca.* 130 ppb, see SI Fig. S4) suggesting that the phenyl ring may be bent over the binding pocket. Analysis of model compound Zn(S21)·2ClO_4_, which lacks the Aib foldamer, supported this suggestion as it showed a NOE between the catechol protons and a methylene at the other end of the crown ether macrocycle (the OC̲H̲_2_CH_2_N protons, see SI Fig. S6 and 7).

**Fig. 6:**
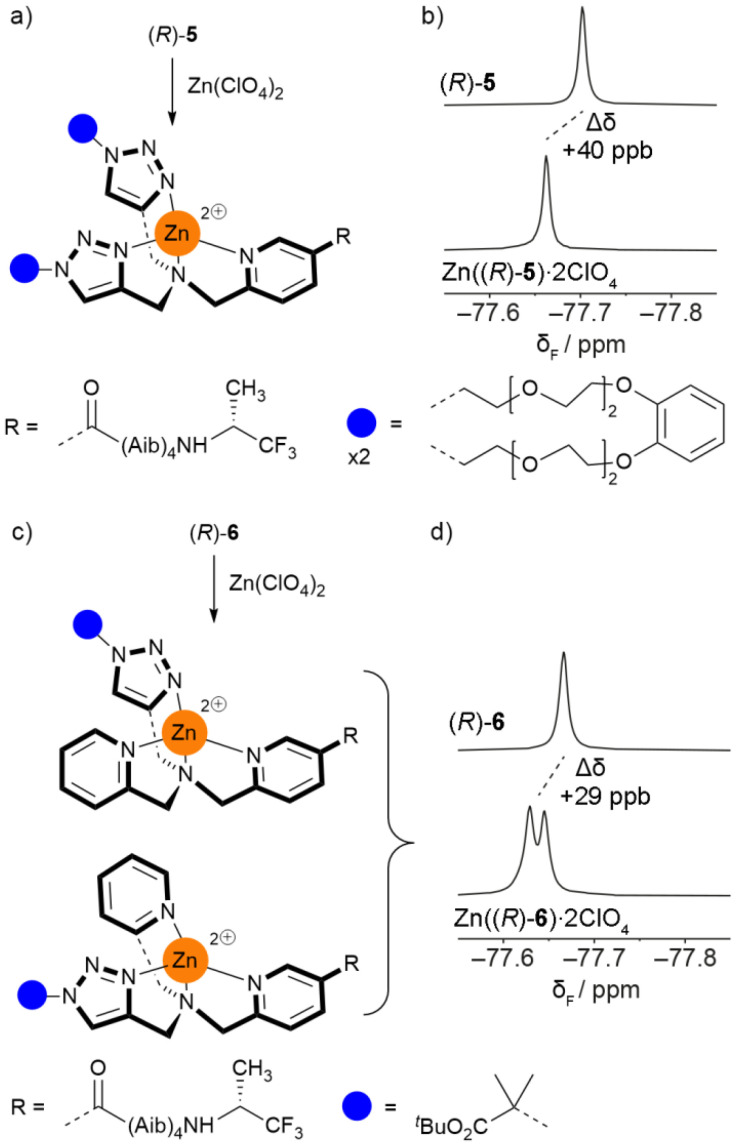
(a and b) Complexation of Zn(ii) to (*R*)-5 in CD_3_CN gives a small change in *δ*_F_(CF_3_) in the ^19^F NMR spectrum. (c and d) Complexation of Zn(ii) to (*R*)-6 in CD_3_CN causes splitting of the CF_3_ resonance in the ^19^F NMR spectrum due to the generation of a stereogenic centre at the N-terminus and the production of diastereomers.

Although the binding pocket of Zn((*R*)-6)·2ClO_4_ is less symmetric than the others, analogous behaviour was observed upon addition of zinc(ii) perchlorate (see SI Fig. S8 and 9). However, unlike the foldamers with symmetric binding pockets (*e.g.* (*R*)-5, Fig. 6a), the addition of zinc(ii) perchlorate to (*R*)-6 caused the ^19^F singlet to split into two overlapping singlets (Fig. 6d, Δ*δ* = 29 ppb). This is consistent with complexation to Zn(ii) generating a chiral centre at the N-terminus (Fig. 6c), which in conjunction with the chiral (*R*)-TFEA group leads to the formation of diastereomeric complexes with distinct CF_3_ resonances. Addition of EDTA to sequester the zinc(ii) supported this hypothesis, as the two peaks merged and returned to their original position (see SI Fig. S9).

### Complexation studies with anions in CD_3_CN

#### Binding to acetate

As a simple anion for initial binding studies, achiral tetra-*n*-butylammonium (TBA) acetate was used. TBA acetate (up to 2 eq.) was titrated into Zn((*R*)-2)·2ClO_4_. Both ^1^H and ^19^F NMR spectroscopy confirmed that acetate bound to the Zn(ii) pocket. Its enantiomer, Zn((*S*)-2)·2ClO_4_, showed the same changes.

**Fig. 7:**
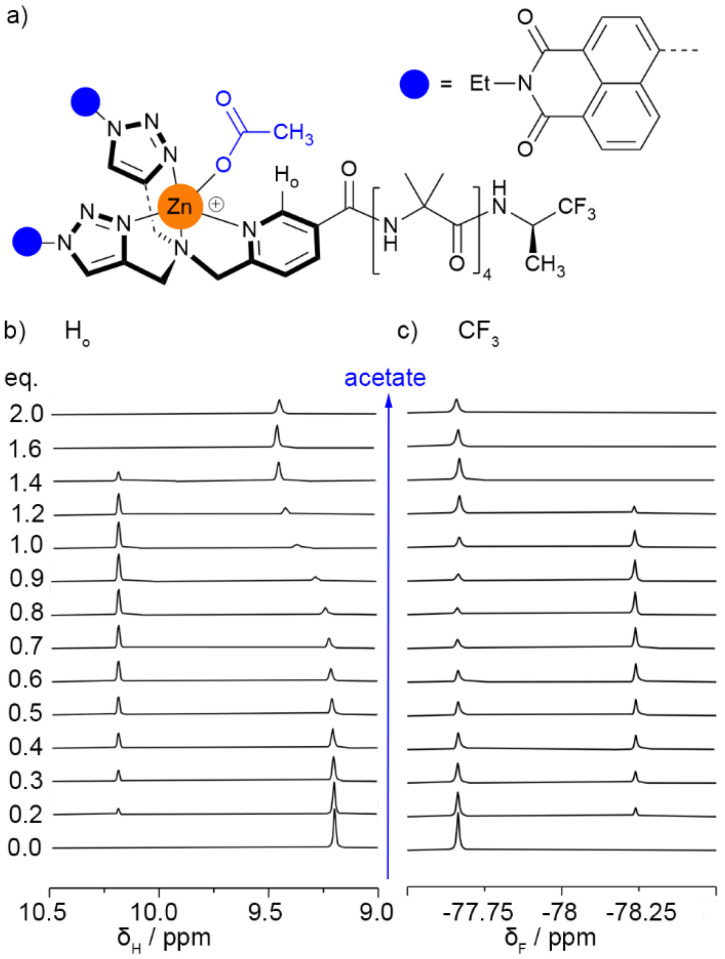
(a) Acetate complexed to Zn((*R*)-2)·2ClO_4_. (b) Partial ^1^H NMR spectra (400 MHz, 298 K) showing the *ortho*-CH resonance during the titration of Zn((*R*)-2)·2ClO_4_ (4.25 mM, 550 μL) in CD_3_CN with TBA acetate (47 mM, up to 2 eq.). (c) Partial ^19^F NMR spectra (376 MHz, 298 K) showing the TFEA reporter region during the titration of Zn((*R*)-2)·2ClO_4_ (4.25 mM, 550 μL) in CD_3_CN with TBA acetate (47 mM, up to 2 eq.). Spectra referenced with C_6_F_6_ at −164.38 ppm.^33^

The *ortho*-pyridyl proton (H_o_) resonance splits into two. One resonance gradually moves downfield by *ca*. 0.213 ppm, suggesting the unbound state and this bound state are in fast exchange on the ^1^H NMR spectroscopy timescale. Another ^1^H NMR resonance for H_o_ also appears further downfield (by *ca*. 1 ppm) after 0.1 eq. ligand has been added; this resonance disappears after 1.4 eq. ligand has been added (Fig. 7b). This H_o_ signal seems to come from a new species that is in slow exchange with the other two species. A corresponding new ^19^F resonance also appears, upfield of the original reporter signal, mirroring the new signal in the ^1^H NMR spectrum by appearing at 0.1 eq. and disappearing at 1.4 eq. This mirroring indicates that the new signals arise from a single species. Indeed after this new peak has disappeared, the ^19^F NMR spectrum showed no shift from the uncomplexed ^19^F peak (Fig. 7c), confirming that an achiral carboxylate has no effect over the *P*/*M* ratio. Diffusion ordered spectroscopy (DOSY) ^1^H spectra of Zn((*R*)-2)·2ClO_4_ with and without 0.7 eq. of TBA acetate confirmed that these new peaks belong to a single separate species. The DOSY data also shows that this species is larger than uncomplexed Zn((*R*)-2)·2ClO_4_, with a hydrodynamic radius of 13.6 Å compared to 10.2 Å for the acetate-free foldamer (see SI Section S4.3.3). These DOSY data suggest the new species may involve more than one foldamer.

### Binding to chiral anions

In previous work, Boc-d-Pro, Boc-d-Pip and *S*-TiPSY, were all shown to produce an *M* screw-sense in Zn((*R*)-2)·2ClO_4_ or Zn((*S*)-2)·2ClO_4_.^26^ The maximum h.e._0_ (the helical excess induced adjacent to the chiral group) of each was estimated by interpolation of Δ*δ*_F_(CF_3_) into our previously reported calibration curve (see SI Section S5),^25,26^ giving h.e._0_ values of −21%, −7% and −23% respectively.^34^ Although these values are half (or less) of the h.e._0_ values that these ligands induced in Zn(1)·2ClO_4_,^35^ this performance is better than other analogues of Zn(1)·2ClO_4_ that had the quinolyl or pyridyl motifs replaced by pyridyl or triazolyl respectively; these gave no clear relays of chirality. Instead complex behaviour was observed, including equilibria with unfavourable exchange kinetics for NMR studies and low solubility.^14,35^ To understand how replacing the quinolyl groups with triazolyl avoids these problems, these three chiral anions (Fig. 4) were titrated into Zn(BTPA)-capped foldamers and the equilibria monitored by ^1^H and ^19^F NMR spectroscopy.

**Fig. 8:**
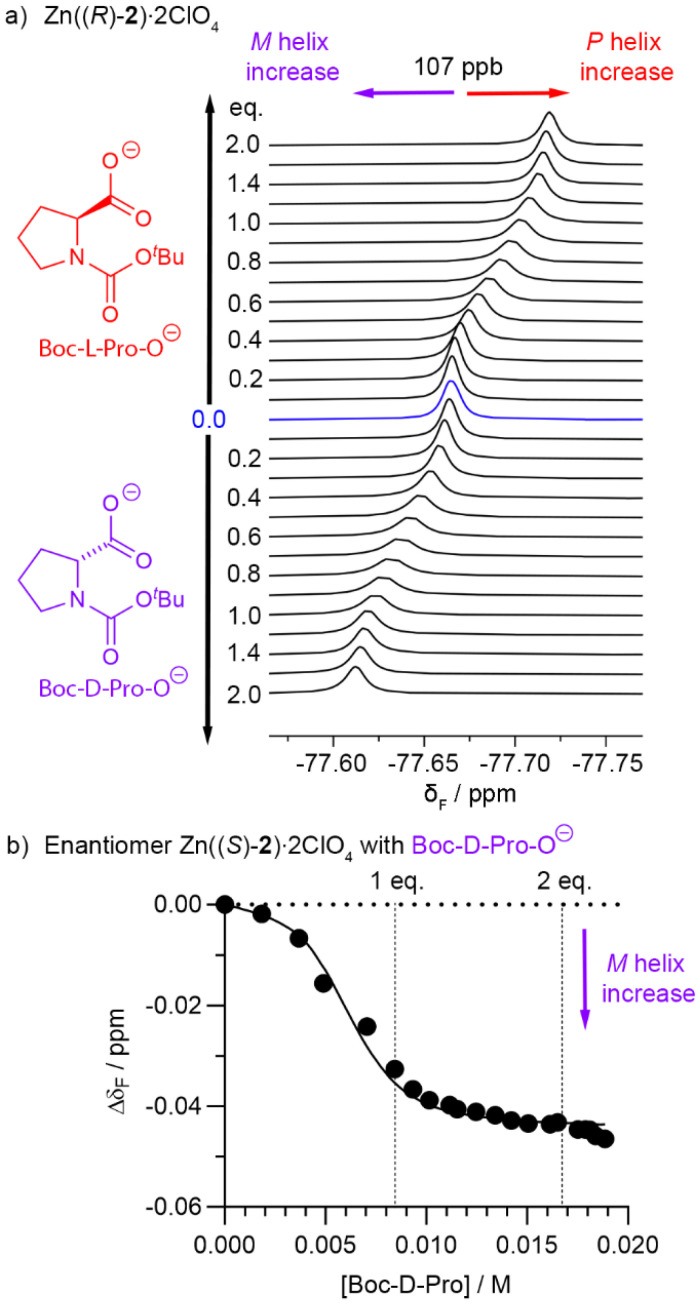
(a) Partial ^19^F NMR spectra (376 MHz, 298 K) showing the CF_3_ region during the titration of Zn((*R*)-2)·2ClO_4_ (4.25 mM, 550 μL) in CD_3_CN with 47 mM Boc-Pro (up to 2 eq. and 2.4 eq. 2,6-lutidine). C_6_F_6_ as an internal standard, referenced at −164.38 ppm.^33^ This internal standard gave *δ*_F_(CF_3_)_0_ as −77.6648 ppm. (b) Representative data fitting of Δ*δ*_F_(CF_3_) using Dynafit during the titration of Zn((*S*)-2)·2ClO_4_ in CD_3_CN with Boc-d-Pro. Binding model: 2 : 1 [Zn((*S*)-2)·2ClO_4_]/[Boc-d-Pro] with *K*_11_ = 1 × 10^5^ M^−1^ and *K*_21_ = 2 × 10^3^ M^−1^. Conditions: [Zn((*S*)-2)·2ClO_4_] = 8.36 mM, [Boc-Pro] = 0–18.9 mM, [2,6-lutidine] = 0–22.7 mM. CFCl_3_ as an internal standard, referenced at −1.14 ppm.^31,33^ This internal standard gave *δ*_F_(CF_3_)_0_ as −77.6205 ppm.

Both Zn((*R*)-2)·2ClO_4_ and Zn((*S*)-2)·2ClO_4_ were titrated with up to 2 eq. Boc-d-Pro or Boc-l-Pro in the presence of 2,6-lutidine (a non-coordinating base, 1.2 eq. with respect to Boc-Pro).^9*a*^ The four combinations give pairs of enantiomeric mixtures, *e.g.* Zn((*R*)-2)·2ClO_4_/Boc-l-Pro is enantiomeric with Zn((*S*)-2)·2ClO_4_/Boc-d-Pro (Fig. 8) and gives identical NMR spectra during the titration.

No significant problems with exchange kinetics or solubility were observed. The ^1^H NMR spectra of Zn((*R*)-2)·2ClO_4_ showed a downfield shift of the *ortho*-pyridyl (H_o_) resonance with Boc-d-Pro and Boc-l-Pro (with 2,6-lutidine, see SI Fig. S14). As observed for acetate, this shift was gradual with increasing ligand concentration, which is indicative of fast exchange between the unbound and ligand-bound states on the ^1^H NMR spectroscopy time-scale at 298 K. However, unlike during the addition of TBA acetate, no additional H_o_ signal appeared further downfield in the ^1^H NMR spectrum. Instead the resonance became much weaker and quite broad (albeit still visible) between 0.3 and 1 eq. of Boc-Pro. The ^1^H NMR resonances from the (*R*)-TFEA reporter were little affected by the addition of carboxylate.

In the ^19^F NMR spectrum, the titration of up to 2 eq. Boc-d-Pro (with 2,6-lutidine) into Zn((*R*)-2)·2ClO_4_ gave a gradual downfield shift in *δ*_F_(CF_3_), confirming an increase in the proportion of *M* helix (Fig. 8a). Conversely, titration of Boc-d-Pro into the enantiomer Zn((*S*)-2)·2ClO_4_ gave a gradual upfield shift in *δ*_F_(CF_3_); this is also consistent with an increase in the proportion of *M* helix (Fig. 8b). Plotting Δ*δ*_F_(CF_3_) against concentration revealed a clear sigmoidal profile. Above 2 eq. Boc-d-Pro, *δ*_F_(CF_3_) remaining constant. The sigmoidal titration profiles for Zn((*R*)-2)·2ClO_4_ and Zn((*S*)-2)·2ClO_4_ are consistent with the formation of intermediates at sub-stoichiometric Boc-Pro that are not visible in the ^19^F NMR spectrum.

Titration with Boc-d-Pip produced similar effects to Boc-d-Pro. Addition of Boc-d-Pip/2,6-lutidine to Zn((*R*)-2)·2ClO_4_ in CD_3_CN resulted in strong broadening of the H_o_ resonance in the ^1^H NMR spectrum with the concurrent appearance of a new, downfield broadened peak at 9.46 ppm (see SI Fig. S18). Both H_o_ resonances were very weak between 0.3 and 1.0 equivalents of ligand (see SI Section S4.3.6) but the observation of two, albeit broadened, resonances during the titration suggests slower exchange on the ^1^H NMR spectroscopy timescale than Boc-Pro. In contrast, *δ*_F_(CF_3_) in the ^19^F NMR spectrum gradually shifts downfield, consistent with fast exchange between free and bound foldamer. *δ*_F_(CF_3_) follows a sigmoidal profile during the titration but with extensive signal broadening between 0.4 and 1 eq. Boc-d-Pip (see SI Fig. S19).

**Fig. 9:**
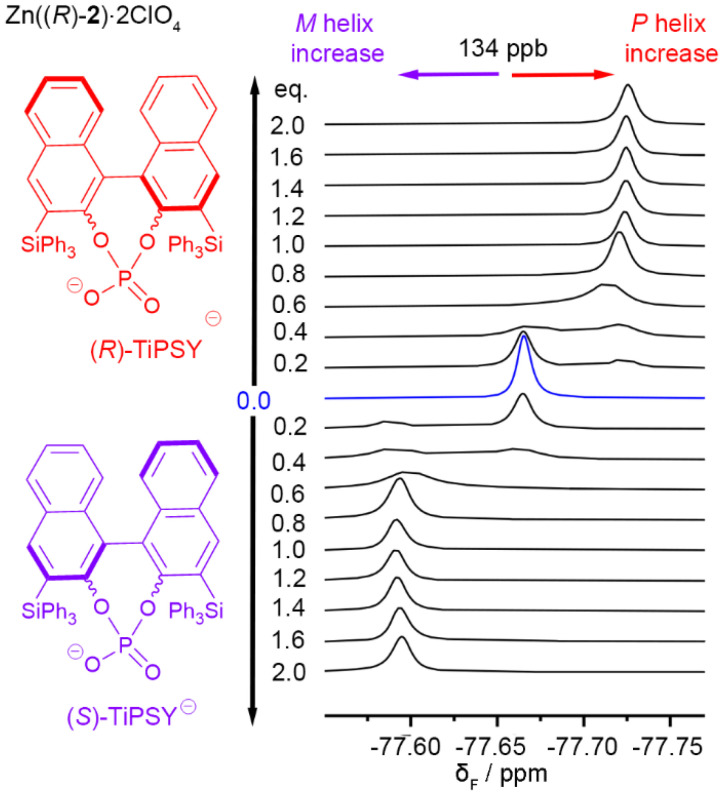
Partial ^19^F NMR spectra (376 MHz, 298 K) showing the CF_3_ region for the titration of Zn((*R*)-2)·2ClO_4_ (4.25 mM, 550 μL) in CD_3_CN with 47 mM TiPSY (up to 2 eq. and 2.4 eq. 2,6-lutidine). C_6_F_6_ as an internal standard, referenced at −164.38 ppm.^33^

Like carboxylates, phosphates are reported to complex to zinc-tetraamine complexes,^36–38^ so the chiral TiPSY anion was also assessed (Fig. 4). TiPSY/2,6-lutidine however produced different behaviour upon titration into Zn((*R*)-2)·2ClO_4_. The ^1^H and ^19^F NMR spectra show the bound and unbound states are now in slow exchange (Fig. 9 and see SI Fig. S20–22), with integration of the signals providing no evidence for the formation of an intermediate species during the addition of TiPSY/2,6-lutidine. The TiPSY anion is a much larger ligand than acetate, Boc-Pro-O^−^ or Boc-Pip-O^−^, perhaps creating a steric block to the formation of intermediate complexes that involve more than one foldamer.

Changing from the flat aromatic moieties in Zn((*S*)-2)·2ClO_4_ to the 2-isobutyrate of Zn((*S*)-3)·2ClO_4_ was hoped to encumber the binding site and allow bound ligands to better control helical screw-sense. Adding Boc-d-Pro/2,6-lutidine to Zn((*S*)-3)·2ClO_4_ induced an *M*-helical bias, the same as for Zn((*S*)-2)·2ClO_4_ (Fig. S24). Indeed the induction of *M* helix by Boc-d-Pro-O^−^ (and *vice versa* for Boc-l-Pro-O^−^) was observed for all BTPA-capped Aib foldamers tested (see SI Section S5), which is the same screw-sense induced in BQPA-capped Aib foldamers by Boc-d-Pro-O^−^.^9*a*,14,15,39^ The kinetics of exchange in this complex are slower and more comparable to Boc-Pip with Zn((*S*)-2)·2ClO_4_. For Zn((*S*)-3)·2ClO_4_, interpolating the induced chemical shifts for Boc-d-Pro (−81 ppb; +76 ppb for Boc-l-Pro) into the calibration curve showed the h.e._0_ (32%) is greater than for Zn((*S*)-2)·2ClO_4_ (21%, see Table 1).

**Table 1:** Calculated helical excess (h.e._0_) values induced by excess Boc-Pro/2,6-lutidine (≥2 eq.) for Zn(2–6)·2ClO_4_. Values calculated from shifts of resonances (Δ*δ*_F_(CF_3_)) in the ^19^F NMR spectra (downfield for Zn((S)-3)·2ClO_4_, upfield for the others)

Foldamer	h.e._0 _a
Zn((*R*)-2)·2ClO_4_	+21% (with Boc-l-Pro)
Zn((*S*)-3)·2ClO_4_	+32% (with Boc-l-Pro)^b^
Zn((*R*)-4)·2ClO_4_	+29% (with Boc-l-Pro)
Zn((*R*)-5)·2ClO_4_	+29% (with Boc-l-Pro)
Zn((*R*)-6)·2ClO_4_	+21% (with Boc-l-Pro)

a h.e._0_ values are estimated from Δ*δ*_F_(CF_3_) values according to a nonlinear model^25^ (see SI Section S5).

b The spectroscopic changes are reversed but Boc-l-Pro still induces *P* helix.

Very similar behaviour was observed for the titration of Zn((*R*)-4)·2ClO_4_ and Zn((*R*)-5)·2ClO_4_ with Boc-Pro (either d or l) under analogous experimental conditions (see SI Sections S4.5 and S4.6 respectively). Both showed fast exchange between bound and unbound foldamer and both gave h.e._0_ values of +29% after addition of 2 eq. Boc-l-Pro/2,6-lutidine (Table 1). As observed for Zn(1)·2ClO_4_,^15^ fast carboxylate exchange at zinc(ii) on the NMR spectroscopy timescale was confirmed and the non-coordinating base 2,6-lutidine only bound weakly (see SI Fig. S35). The former was confirmed by using different scalemic mixtures of Boc-Pro; *δ*_F_(CF_3_) correlated with the ee of the mixtures (see SI Fig. S37). Notably, addition of a 1 : 1 mixture of Boc-(d/l)-Pro to Zn((*R*)-4)·2ClO_4_ gave *δ*_F_(CF_3_) at the same position, within the experimental error, as that of uncomplexed Zn((*R*)-4)·2ClO_4_ (*i.e.* h.e._0_ = 0 in both cases).

As expected, given that it exists as two diastereomers, titration of unsymmetrical Zn((*R*)-6)·2ClO_4_ with Boc-Pro (either d- or l-) gave complex data with multiple CF_3_ resonances observed (see SI Section S4.7). Nonetheless these changes were broadly similar to those observed with symmetric Zn((*R*)-4)·2ClO_4_.

### Binding model and estimation of carboxylate affinity for Zn(2–5)·2ClO_4_ in CD_3_CN

The equilibria between different Boc-Pro/foldamer complexes were modelled. The sigmoidal profiles in the ^1^H NMR and ^19^F NMR data during titrations with Boc-Pro or Boc-Pip are similar to that observed during the TBA acetate titration of Zn((*R*)-2)·2ClO_4_ and suggest that an intermediate is formed. This is presumed to be a 2 : 1 foldamer : ligand complex. We believe that because of the exchange kinetics when Boc-Pro or Boc-Pip (each with 2,6-lutidine) are used as titrants, this intermediate species is not observable by NMR spectroscopy for these anions. The structure of this intermediate 2 : 1 complex is unknown but we speculate that the carboxylate is bridging between zinc centres, which has been reported for similar complexes.^40^ This may be a coordination mode permitted by the relatively open face of the BTPA binding pocket compared to the encumbered BQPA binding pocket of Zn(1)·2ClO_4_. This bridged species may be disfavoured for the large bulky TiPSY anion.

**Fig. 10:**
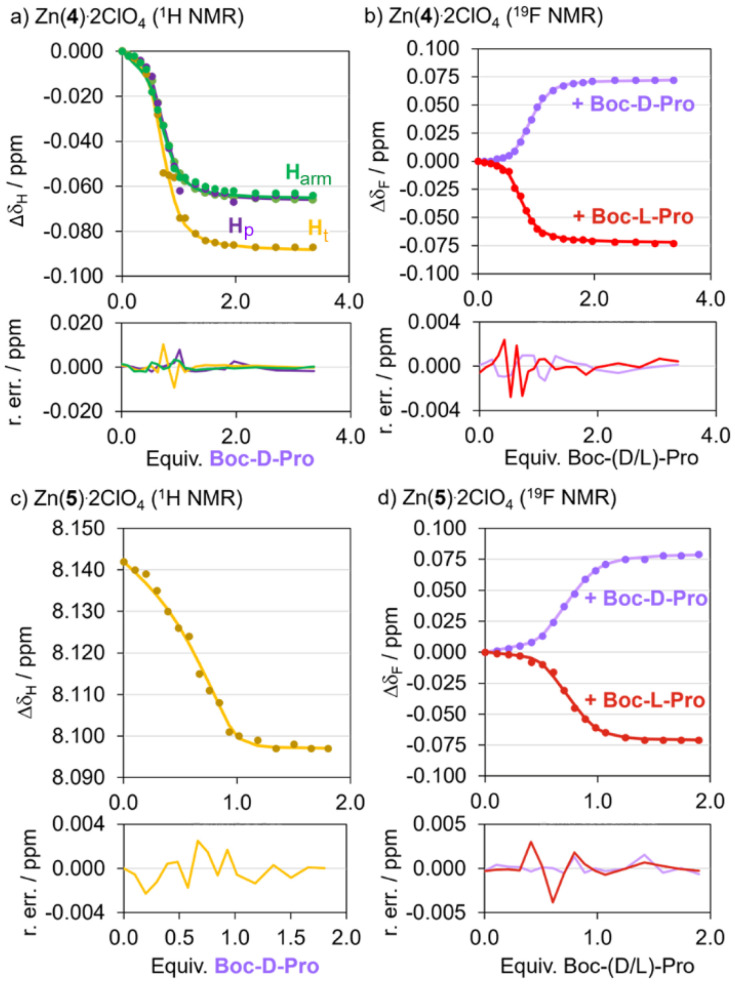
Representative data fitting with Suprafit.^41^ For fits, see the SI Sections S4.5 and S4.6. (a) Global fit of ^1^H protons of Zn((*R*)-4)·2ClO_4_ titrated with Boc-d-Pro. (b) Fits of the ^19^F peak of Zn((*R*)-4)·2ClO_4_ titrated with Boc-d-Pro (purple) or Boc-l-Pro (red). (c) Fit of the triazole proton of Zn((*R*)-5)·2ClO_4_ (H_t_) titrated with Boc-d-Pro. (d) Fits of the ^19^F peak of Zn((*R*)-5)·2ClO_4_ titrated with Boc-d-Pro (purple) or Boc-l-Pro (red). Binding model: 2 : 1 [foldamer]/[anion] (see eqn (1) and (2) in SI Section S4.2). Conditions: [Zn((*R*)-4)·2ClO_4_] = 2 mM, [Boc-Pro] = 0–6.7 mM, [2,6-lutidine] = 0–7.2 mM. [Zn((*R*)-5)·2ClO_4_] = 2 mM, [Boc-Pro] = 0–3.6 mM, [2,6-lutidine] = 0–4.3 mM. ^19^F NMR spectra referenced with C_6_F_6_ at –164.38 ppm.^31,33^ R. err.: residual error (in ppm).

In order to estimate the affinity of Boc-Pro for the Zn(BTPA)-capped foldamers, we used SupraFit and Dynafit to calculate the binding constants (Fig. 8b and 10, also see the SI).^41,42^ We used a 2 : 1 foldamer : anion binding model for data fitting, with the binding constant *K*_11_ representing the formation of the 1 : 1 complex and the binding constant *K*_21_ representing the formation of the intermediate 2 : 1 complex from the 1 : 1 complex and another equivalent of foldamer. Since for acetate binding, uncomplexed Zn((*R*)-4)·2ClO_4_ and the 1 : 1 complex are in fast exchange with each other but the 2 : 1 complex is not, we assume the 2 : 1 complex should not affect the chemical shift of the 1 : 1 complex and fitting of the latter's chemical shift data should give an estimate of both binding constants. Nonetheless fitting the data to two equilibria presents challenges, with small uncertainties in concentration leading to significant differences in the individual binding constants *K*_11_ and *K*_21_.

The titration of Zn((*S*)-2)·2ClO_4_ with Boc-d-Pro gave ^19^F NMR data (Fig. 8b) and ^1^H data (see SI Fig. S16) that fitted a 2 : 1 binding model adequately using *K*_11_ = 1 × 10^5^ M^−1^ and *K*_21_ = 2 × 10^3^ M^−1^. The titration of foldamers Zn((*R*)-4)·2ClO_4_ and Zn((*R*)-5)·2ClO_4_ with both Boc-l-Pro and Boc-d-Pro could also be adequately fitted to a 2 : 1 binding model (Fig. 10). Within the uncertainty associated with fitting the formation of multiple complexes, there is reasonable agreement (within an order of magnitude) between the binding constants calculated for foldamers Zn((*R*)-4)·2ClO_4_ and Zn((*R*)-5)·2ClO_4_ (approximate values: *K*_11_ = 1 to 4 × 10^6^ M^−1^ and *K*_21_ = 1 to 2 × 10^4^ M^−1^); these are an order of magnitude higher than the analogous values for Zn((*S*)-2)·2ClO_4_. These values for *K*_11_ (*ca*. ∼10^6^ M^−1^) are similar to *K*_11_ for the complexation of Boc-Pro to Zn(1)·2ClO_4_ (4 × 10^6^ M^−1^) in CD_3_CN, although in that case there was no indication of an intermediate complex.^35^ The *K*_21_ binding constants (*ca*. 10^4^ M^−1^) are approximately 100-fold smaller than *K*_11_, which is consistent with a steric barrier inhibiting the formation of this 2 : 1 complex. The similarity of the Boc-Pro binding constants for Zn((*R*)-4)·2ClO_4_ and Zn((*R*)-5)·2ClO_4_ indicate that changing the oligoethyleneglycol for a crown ether has little influence on the binding of carboxylates. Estimated *K* values for Zn((*R*)-4–6)·2ClO_4_ are summarised in Tables S1–S5 (see the SI).

## Conclusions

The modular nature of CuAAC allows the construction of bis(triazole)pyridyl (BTPA) metal ion chelation sites that are flanked by different substituents, *e.g.* by a fluorophore or a crown ether bridge. Carboxylate and phosphate ligands both bind tightly to the Zn(BTPA) group, with the Zn(BTPA) structure allowing bound ligands to induce local conformational changes in the adjacent 3_10-_helical Aib foldamer body. Corresponding increases in the proportion of either the *P* or the *M* screw-sense were detected by ^19^F NMR spectroscopy using the recently developed 1-(trifluoromethyl)-ethylamido (TFEA) reporter group.^25^

The strength of helical induction by different ligands was lower than that reported for the same ligands when bound to a previously described bis(quinolyl)pyridyl (BQPA)-capped foldamer. The greater hindrance created by the quinolyl arms compared to the more open face presented by the triazoles in Zn(BTPA) complexes is proposed to lead to the better performance of BQPA. Lower steric encumbrance around the Zn(BTPA) site is also proposed to permit additional coordination equilibria, with new intermediates observed at sub-stoichiometric carboxylate that are not found with Zn(BQPA) foldamers. DOSY and titration data suggest these intermediates are (foldamer)_2_(carboxylate) complexes.

Nonetheless, the new BTPA binding site does not suffer from the drawbacks observed in previous replacements for BQPA.^14^ Despite showing similar coordination geometries to Zn(1)·2ClO_4_, either replacing the quinolyl arms with pyridyl arms or replacing the pyridyl link with a triazolyl link led to foldamers with undesirable characteristics, including poor solubility, unfavourable ligand exchange rates on the NMR timescale, and inefficient relays of conformational information from the bound carboxylate (*i.e.* Boc-Pro). In contrast, all tested Zn(BTPA)-capped foldamers were soluble, had clear resonances at room temperature and possessed an effective conformational relay. These properties show that the modular BTPA motif is a versatile alternative to BQPA.

The versatility of CuAAC chemistry also permitted the creation of an unsymmetrical binding site on an Aib foldamer. The unsymmetrical binding site generated a chirogenic centre at the N-terminus upon Zn(ii) complexation. The conformational preference of each handedness of the chirogenic Zn(ii) complex is relayed along the foldamer body to the chiral CF_3_-containing reporter group, leading to different ^19^F NMR spectroscopic outputs from each diastereomer. The net effect is to allow an achiral messenger (Zn(ii)) to produce a spectroscopic output from the remote chiral TFEA reporter group.

With the Zn(BTPA) ligand binding site shown to mediate ligand-induced conformational change, the modular assembly of functional binding sites by CuAAC should lend itself to the generation of more complex constructs that better mimic natural binding sites in proteins. In this way ligand-triggered conformational change in larger and more functional foldamers will become possible.

## Author contributions

S. J. W. conceived the idea, acquired the funding, administered the project. F. D. S., B. D. and S. J. W. designed the experiments, analysed the data and wrote the manuscript. F. D. S. and B. D. and carried out the experimental work. S. J. W. provided resources and supervision.

## Conflicts of interest

There are no conflicts to declare.

## Supplementary Material

OB-023-D5OB01226K-s001

## Data Availability

The data supporting this article have been included as part of the SI. See DOI: https://doi.org/10.1039/d5ob01226k.
