# Supplementary material for: Modular synthesis of zinc(ii)-bis(triazole) recognition sites for the conformational control of foldamers
Source: Org Biomol Chem. 2025 Aug 28;23(38):8719–27. doi: 10.1039/d5ob01226k (PMC12421298; doi:10.1039/d5ob01226k)
Supplement: OB-023-D5OB01226K-s001 [file OB-023-D5OB01226K-s001.pdf]

Supplementary Information (SI) for Organic & Biomolecular Chemistry.
This journal is © The Royal Society of Chemistry 2025

Supporting Information

Modular synthesis of zinc(II)-bis(triazole) recognition sites for the conformational control of foldamers

Flavio della Sala,[1,2] Benedicte Doerner,[1,2] and Simon J. Webb*,[1]

----

1. Department of Chemistry
University of Manchester
Oxford Road, Manchester M13 9PL, United Kingdom
E-mail: S.Webb@manchester.ac.uk

2. Manchester Institute of Biotechnology
University of Manchester
131 Princess Street, Manchester M1 7DN, United Kingdom

TABLE OF CONTENTS

1. General experimental procedures **4**
1.1 Abbreviations 5
2. Synthetic procedures **6**
2.1 Reported experimental procedures 7
2.2 Synthetic procedures for new compounds 7
2.2.1 Compound (*S*)-**3** 7
2.2.2 Compound (*R*)-**4** 8
2.2.3 Compound (*R*)-**5** 8
2.2.4 Compound (*R*)-**6** 9
2.2.5 Compound **S14** 10
2.2.6 Compound **S16** 10
2.2.7 Compound **S17** 11
2.2.8 Compound (*R*)-**7** 11
2.2.9 Compound **S18** 12
2.2.10 Compound (*R*)-**8** 12
2.2.11 Compound **10** 13
2.2.12 Compound **S19** 13
2.2.13 Compound (*R*)-**11** 14
2.2.14 Compound **S20** 14
2.2.15 Compound **S21** 15
3. Synthesis of Zn(II) perchlorate foldamer complexes **16**
3.1 General procedure 16
3.2 Compound Zn((*S*)-**3**)·$2ClO_4$ 16
3.3 Compound Zn((*R*)-**4**)·$2ClO_4$ 17
3.4 Compound Zn((*R*)-**5**)·$2ClO_4$ 17
3.5 Compound Zn(**S21**)·$2ClO_4$ 18
3.6 Compound Zn((*R*)-**6**)·$2ClO_4$ 18
4. Titrations with anions **24**
4.1 General procedures 24
4.2 Fitting of titration data 24
4.3 Host: Zn((*R*)-**2**)·$2ClO_4$ 25
4.3.1 Titration with tetrabutylammonium acetate (^{1}H NMR) 25
4.3.2 Titration with tetrabutylammonium acetate (^{1}H NMR) 27
4.3.3 Diffusion ordered spectroscopy (DOSY) with TBA acetate 28
4.3.4 Titration with Boc-Pro/2,6-lutidine (^{1}H NMR) 30
4.3.5 Titration with Boc-Pro/2,6-lutidine (^{19}F NMR) 31
4.3.6 Titration with Boc-Pip/2,6-lutidine (^{1}H NMR) 33
4.3.7 Titration with Boc-Pip/2,6-lutidine (^{19}F NMR) 34
4.3.8 Titration with TiPSY/2,6-lutidine (^{1}H NMR) 35

4.3.9 Titration with TiPSY/2,6-lutidine (^{19}F NMR) 38
4.4 Host: Zn((*S*)-**3**)·2ClO$_4$ 39
4.4.1 Titration with Boc-Pro/2,6-lutidine (^{1}H NMR) 39
4.4.2 Titration with Boc-Pro/2,6-lutidine (^{19}F NMR) 40
4.5 Host: Zn((*R*)-**4**)·2ClO$_4$ 41
4.5.1 Titration with Boc-Pro/2,6-lutidine (^{1}H NMR) 41
4.5.2 Titration with Boc-Pro/2,6-lutidine (^{19}F NMR) 44
4.5.3 Titration with tetrabutylammonium acetate (^{1}H NMR) 46
4.5.4 Titration with tetrabutylammonium acetate (^{19}F NMR) 47
4.5.5 Control: titration with 2,6-lutidine (^{1}H NMR) 48
4.6 Host: Zn((*R*)-**5**)·2ClO$_4$ 50
4.6.1 Titration with Boc-Pro/2,6-lutidine (^{1}H NMR) 50
4.6.2 Titration with Boc-Pro/2,6-lutidine (^{19}F NMR) 52
4.7 Host: Zn((*R*)-**6**)·2ClO$_4$ 54
4.7.1 Titration with Boc-Pro/2,6-lutidine (^{1}H NMR) 54
4.7.2 Titration with Boc-Pro/2,6-lutidine (^{19}F NMR) 55
4.7.3 Control: Zn((*R*)-**6**)·2ClO$_4$ with an excess of Boc-L-Pro and 2,6-lutidine 57
5. Modelling of the ^{19}F chemical shift in CD_3CN 58
6. NMR spectra of new compounds 61
6.1 Compound (*S*)-**3** 61
6.2 Compound (*R*)-**4** 63
6.3 Compound (*R*)-**5** 65
6.4 Compound (*R*)-**6** 67
6.5 Compound **S13** 69
6.6 Compound **S16** 71
6.7 Compound **S17** 72
6.8 Compound (*R*)-**7** 73
6.9 Compound **S18** 75
6.10 Compound (*R*)-**8** 76
6.11 Compound **10** 78
6.12 Compound **S19** 79
6.13 Compound (*R*)-**11** 80
6.14 Compound **S20** 82
6.15 Compound **S21** 83
7. References 84

1. General experimental procedures

Unless stated otherwise, chemicals were purchased from Sigma-Aldrich Co. Ltd., Dorset, UK. and from Fluorochem, Derbyshire. Anhydrous THF was obtained by drying with activated 4 Å molecular sieves. Anhydrous acetonitrile and *N*,*N*-dimethylformamide (DMF) were purchased from Sigma-Aldrich. Petroleum ether refers to the fraction of light petroleum ether boiling between 40 and 60 °C. Dichloromethane and toluene were dried over molecular sieves 4 Å under a nitrogen atmosphere. All other solvents and commercially available reagents were used as received without further purification. Foldamer starting materials were synthesised following the procedures referenced in Section 2.1.

All reactions were carried out using oven-dried glassware under an atmosphere of nitrogen using standard anhydrous techniques. All synthesised products were dried first on a rotary evaporator followed by further drying under high vacuum to remove any residual solvent. Flash chromatography was performed on silica gel (Merck 60H, 40-60 nm, 230–300 mesh). Analytical thin layer chromatography (TLC) was performed on Macherey Nagel alugram SIL G/UV254 TLC sheets and TLC plates were visualised by UV irradiation (254 nm), a ninhydrin stain or a potassium permanganate stain where appropriate.

NMR spectra were recorded in deuterated solvents using either Brüker AVANCE 400 MHz or Brüker AVANCE 500 MHz spectrometers. Chemical shifts are quoted in parts per million (ppm) and coupling constants (*J*) are quoted in Hz to the nearest 0.5 Hz. ^{1}H NMR spectra were referenced to the residual deuterated solvent peak ($CDCl_3$: 7.27; CD_3OD: 3.31; CD_3CN: 1.94 ppm).[S1] ^{13}C NMR spectra were referenced to the resonance of the solvent ($CDCl_3$: 77.2, CD_3OD: 49.0, CD_3CN: 118.3 ppm).[S1] ^{19}F spectra were referenced to an added standard that was either in the sample or in an coaxial tube (C_6F_6 at –164.38 ppm).[S2] Multiplicities are reported as s (singlet), d (doublet), t (triplet), q (quartet), m (multiplet), broad peak (b) or some combination of these, where appropriate. Assignments of the peaks were performed by analysis of chemical shifts, coupling constants, ^{1}H-^{1}H COSY data, and ^{1}H-^{13}C HSQC data.

Low- and high-resolution mass spectra were recorded by staff at the University of Manchester. Electrospray (ES) spectra were recorded on an Agilent 6530 LC/MS. High resolution mass spectra (HRMS) were recorded on a Thermo Q-Exactive and are accurate to ±0.001 Da.

1.1 Abbreviations

Aib: Aminoisobutyric acid;

Boc: *tert*-Butyloxycarbonyl;

BQPA: Bis(2-quinolymethyl)-(2-pyridylmethyl)amine;

BTPA: Bis(triazole)-(2-pyridylmethyl)amine;

DIPEA: *N*,*N*-Diisopropylethylamine;

EDC·HCl: *N*-Ethyl-*N′*-(3-dimethylaminopropyl)-carbodiimide hydrochloride;

EDTA: Ethylenediaminetetraacetic acid;

ES: Electrospray;

HATU: Hexafluorophosphate azabenzotriazole tetramethyluronium;

h.e.: Helical excess;

HOBt: Hydroxybenzotriazole;

HRMS: High resolution mass spectrometry;

IS: Internal standard;

MS: Mass spectrometry;

NMR: Nuclear magnetic resonance;

Phe: Phenylalanine;

Pip: Pipecolinic acid;

ppm: parts per million;

Pro: Proline

PTPA: (pyridyl)(triazolyl)(pyridyl)amine;

rt: Room temperature;

TBA:Tetrabutylammonium;

TEA: Triethylamine;

(*R*)-TFEA: (*R*)-1-(Trifluoromethyl)ethylamine;

(*S*)-TFEA: (*S*)-1-(Trifluoromethyl)ethylamine;

TiPSY: 3,3’-Bis(triphenylsilyl)-1,1’-binaphthyl-2,2’-diyl hydrogenphosphate;

2. Synthetic procedures

a) Synthesis of (*R*)- and (*S*)-TFEA reporter

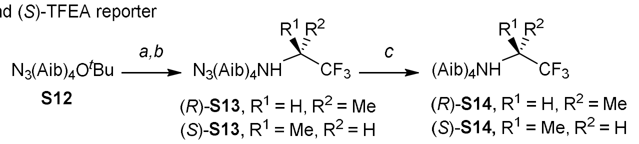

b) Synthesis of BTPA foldamers

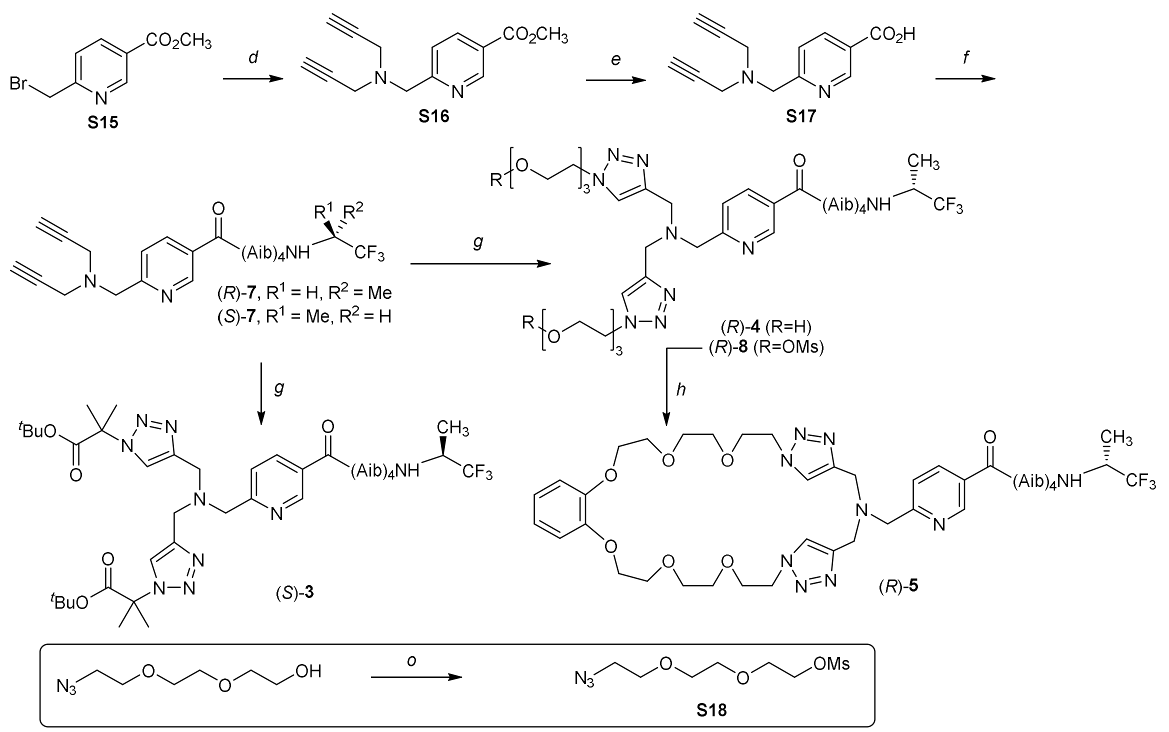

c) Synthesis of PTPA foldamers

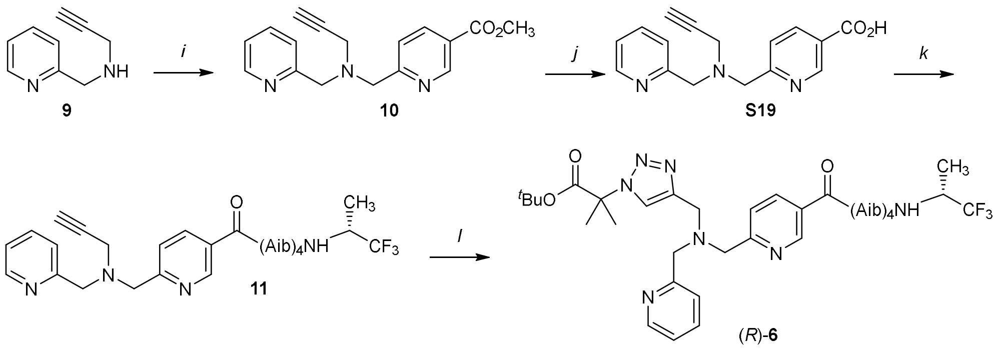

d) Synthesis of BTPA model compound

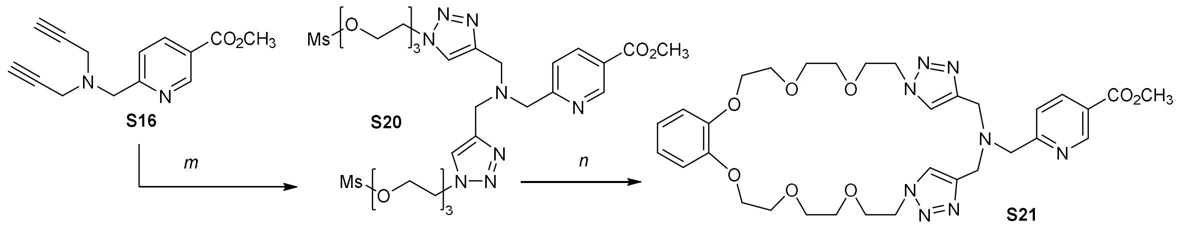

Scheme S1 Synthesis of foldamers. Reagents: *a*. CF_3CO_2H, CH_2Cl_2, rt. *b*. (*R*)-2-Amino-1,1,1-trifluoropropane·HCl, DIPEA, HATU, rt. *c*. H_2/Pd(C), EtOH, rt. *d*. Dipropargylamine, DIPEA, CH_3CN, rt. *e*. KOH, CH_3OH, reflux. *f*. (*R*)- or (*S*)-**S14**, EDC·HCl, HOBt, TEA, CH_3CN, rt. *g*. $N_3CMe_2CO_2{}^tBu$, 8-Azido-3,6-dioxaoctanol or **S18**, $CuSO_4{\cdot}5H_2O$, sodium ascorbate, DMF, rt. *h*. (*R*)-**8**, catechol, Cs_2CO_3, CH_3CN, reflux. *i*. **S15**,[S3] DIPEA, CH_3CN, rt. *j*. KOH, CH_3OH, reflux. *k*. (*R*)-**S14**, EDC·HCl, HOBt, TEA, CH_3CN, rt. *l*. N_3AibO^tBu,[S4] $CuSO_4{\cdot}5H_2O$, sodium ascorbate, DMF, rt. *m*. **S18**, $CuSO_4{\cdot}5H_2O$, sodium ascorbate, DMF, rt. *n*. catechol, Cs_2CO_3, CH_3CN, reflux. *o*. MsCl, CH_2Cl_2, rt.

2.1 Reported experimental procedures

Compounds $N_3(Aib)_4O^tBu$ (**S12**),[S5] $N_3(Aib)_4NHCH(CH_3)CF_3$ ((*R*)-**S13** and (*S*)-**S13**),[S5] methyl 6-(bromomethyl)nicotinate (**S15**),[S3] foldamer (*R*)-**2**, and foldamer (*S*)-**2** were synthesized according to reported procedures.

2.2 Synthetic procedures for new compounds

2.2.1 Compound (*S*)-3

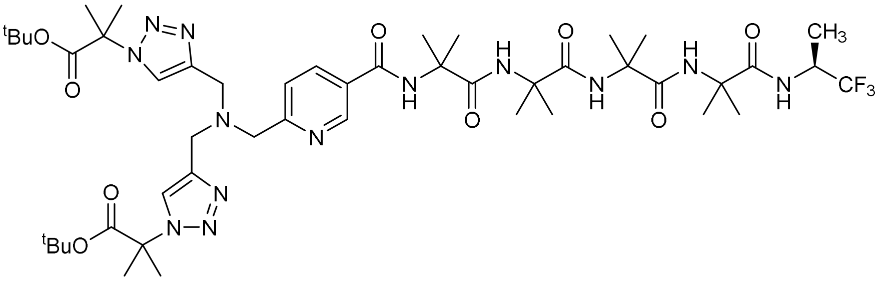

Bis-alkyne (*S*)-**7** (50 mg, 0.075 mmol), $CuSO_4{\cdot}5H_2O$ (3.8 mg, 0.015 mmol) and sodium ascorbate (6 mg, 0.030 mmol) were combined in a flask under Ar. In a separate flask under argon, $N_3CMe_2O^tBu$ (30 mg, 0.158 mmol) was dissolved in dry DMF (1.3 mL) and transferred to the other flask. The solution was left to stir at rt for 3 h, giving a colour change from pale yellow to green to brown. The reaction mixture was diluted with DCM (10 mL) and washed with sat. EDTA solution (5 mL) and 5% LiCl (3 × 5 mL). The organic phase was then dried over $MgSO_4$, filtered, and concentrated under reduced pressure. The resulting crude was purified by column chromatography (SiO_2, DCM/MeOH = 100:0 to 95:5 v/v) to afford the title compound (*S*)-**3** as a pale-yellow solid (51 mg, 65%).

^1H NMR (400 MHz, CD_3CN): δ 8.97 (d, *J* = 1.8 Hz, 1H, H_{Py}), 8.18 (dd, *J* = 8.2, 2.2 Hz,1H, H_{Py}), 7.90 (s, 2H, 2 × $H_{triazole}$), 7.75 (s, 1H, NH), 7.70 (d, *J* = 8.2 Hz ,1H, H_{Py}), 7.56 (s, 1H, NH), 7.56 (d, *J* = 9.3 Hz, 1H, N*H*CHCF$_3$), 7.51 (s, 1H, NH), 7.17 (s, 1H, NH), 4.56 (m, 1H, CF$_3$C*H*), 3.81 (s, 2H, CH_2), 3.78 (s, 4H, 2 × CH_2), 1.85 (s, 12H, 2 × $(CH_3)_2$), 1.51 (s, 3H, CH_3), 1.51 (s, 3H, CH_3), 1.46 (s, 3H, CH_3), 1.42 (s, 3H, CH_3), 1.41 (s, 3H, CH_3), 1.40 (s, 3H, CH_3), 1.36 (s, 3H, CH_3), 1.35 (s, 18H, 2 × $C(CH_3)_3$), 1.33 (s, 3H, CH_3), 1.31 (s, 3H, CH_3) ppm;

^{13}C NMR (101 MHz, $CDCl_3$): δ 176.3 (CO), 176.3 (CO), 175.5 (CO), 175.1 (CO), 171.5 (CO), 167.2 (CO), 164.4 (ArC$_q$), 149.1 (ArCH$_{py}$), 144.5 (ArC$_q$), 136.7 (ArCH$_{py}$), 128.7 (ArC$_q$), 123.5 (ArCH$_{triazole}$), 123.3 (ArCH$_{py}$), 83.2 (*C*(CH$_3$)$_3$), 65.6 (triazole-*C*(CH$_3$)$_2$), 59.6 (CH_2), 58.2 (C$_q$), 57.6 (C$_q$), 57.6 (C$_q$), 57.4 (C$_q$), 49.0 (2 × CH_2), 47.1 (q, *J* = 31.0 Hz, *C*HCF$_3$), 27.8 (2 × $(CH_3)_3$), 26.9 (CH_3), 26.3(CH_3), 26.1(CH_3), 25.7 (2 × triazole-(C(*C*H$_3$)$_2$)$_2$), 25.6 (CH_3), 24.6 (CH_3), 24.4 (CH_3), 24.2 (CH_3), 13.9 (NHCH*C*H$_3$) ppm; the CF_3 quartet was too weak to be identified by ^{13}C NMR spectroscopy.

^{19}F NMR (376 MHz, CD_3CN): δ -77.7 (s, 3F, CF_3) ppm;

MS (ES$^+$, MeCN): *m/z* = 1056.6 ([M+Na]$^+$, 100%);

HRMS (ESI$^+$): *m/z* calcd. for $C_{48}H_{74}O_9N_{13}F_3Na$ ([M+Na]$^+$): 1056.5577, found 1056.5567.

2.2.2 Compound (*R*)-4

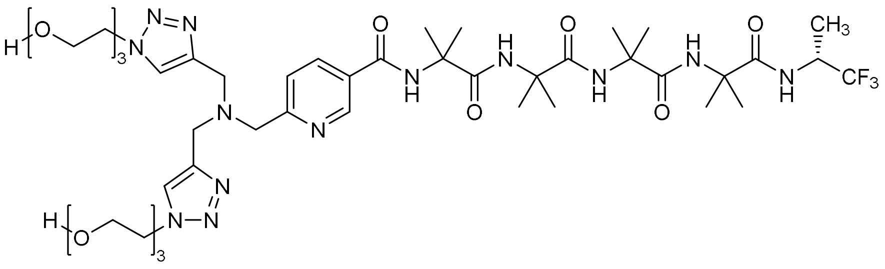

$CuSO_4 \cdot 5H_2O$ (9 mg, 0.04 mmol) and sodium ascorbate (14 mg, 0.07 mmol) were added to a solution of bis-alkyne (*R*)-**7** (120 mg, 0.18 mmol) and 2-[2-(2-azidoethoxy)ethoxy]ethanol (0.8 mL, 0.38 mmol) in dry DMF (3.6 mL). The mixture was stirred under Ar for 3 h. The solvent was then evaporated under high vacuum. The residue was re-dissolved in $CHCl_3$ (10 mL) and washed with saturated EDTA solution (2 mL). The aqueous phase was re-extracted with $CHCl_3$ (3 × 2 mL). The combined organic layers were dried over $MgSO_4$, filtered and evaporated under reduced pressure. The crude was purified by flash chromatography (SiO_2, $CH_2Cl_2/CH_3CN/MeOH$ 5:5:1 to 0:0:1) to afford the title compound as a yellow oil (110 mg, 60%).

^1H NMR (400 MHz, $CDCl_3$): δ 9.00 (d, *J* = 2.2 Hz, 1H, H_{Py}), 8.15 (dd, *J* = 8.2, 2.2 Hz, 1H, H_{Py}), 8.07 (bs, 1H, NH), 7.93 (s, 2H, 2 × $H_{triazole}$), 7.72 (bs, 1H, NH), 7.62–7.54 (m, 3H, 2 × NH + H_{Py}), 6.93 (bs, 1H, NH), 4.61–4.51 (m, 5H, C*H*CF_3 + 2 × CH_2O), 3.84–3.86 (m, 4H, 2 × CH_2O), 3.80 (s, 2H, C*H*$_2$Py), 3.77 (s, 4H, 2 × C*H*$_2$-triazole), 3.68–3.71 (m, 4H, 2 × CH_2O), 3.61 (bs, 8H, 4 × CH_2O), 3.53–3.56 (m, 4H, 2 × CH_2O), 1.56 (s, 6H, 2 × CH_3), 1.50 (s, 3H, CH_3), 1.47 (s, 6H, 2 × CH_3), 1.45 (s, 3H, CH_3), 1.44 (s, 3H, CH_3), 1.36 (s, 3H, CH_3), 1.34 (s, 3H, CH_3).

^{19}F NMR (377 MHz, $CDCl_3$): δ –77.2 (s, 3F, CF_3).

^{13}C NMR (101 MHz, $CDCl_3$): δ 176.0, 174.8, 174.4, 174.2, 166.5, 162.9, 148.4, 143.9, 136.1, 127.8, 124.9, 123.4, 72.7, 70.5, 70.3, 69.4, 61.5, 59.0, 57.6, 57.1, 57.0, 56.9, 50.2, 48.3, 27.2, 26.6, 25.9, 24.1, 23.8, 23.6, 13.5.

HRMS (ES$^+$, MeOH): m/z calculated for $C_{44}H_{70}O_{11}N_{13}F_3Na$ $[M+Na]^+$ 1036.5162, found 1036.5139.

2.2.3 Compound (*R*)-5

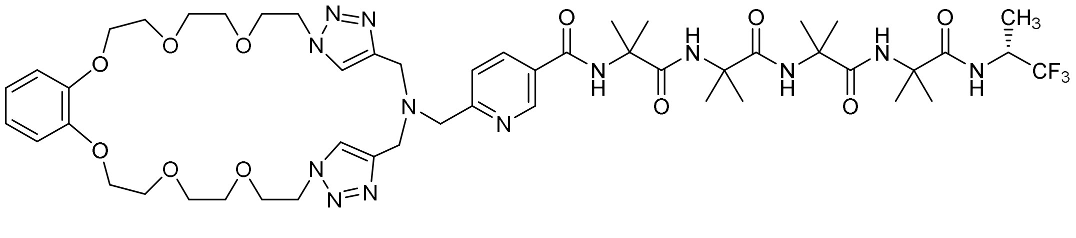

Under N_2, catechol (21 mg, 0.19 mmol) and Cs_2CO_3 (223 mg, 0.68 mmol) were dissolved in degassed CH_3CN (100 mL). Then, a solution of compound (*R*)-**8** (200 mg, 0.17 mmol) in degassed CH_3CN (10 mL) was added and the reaction mixture was heated to reflux for 3 days. The mixture was then left to cool at rt, evaporated under reduced pressure and re-dissolved in $CHCl_3$ (10 mL). The organic layer was washed with H_2O (10 mL) and the aqueous layer was re-extracted with $CHCl_3$ (2 × 10 mL). The combined organic layers were dried over $MgSO_4$, filtered and evaporated under reduced pressure. The crude was purified by flash chromatography (Al_2O_3, $CH_2Cl_2/CH_3CN/CH_3OH$ 1:1:0 to 5:5:1) to afford the title compound as a yellow oil (103 mg, 56%).

^{1}H NMR (400 MHz, $CDCl_3$): δ 9.02 (d, *J* = 2.3 Hz, 1H, H_{Py}), 8.10 (dd, *J* = 8.2, 2.3 Hz, 1H, H_{Py}), 7.75 (s, 2H, 2 × $H_{triazole}$), 7.66 (bs, 1H, NH), 7.60 (d, *J* = 8.2 Hz, 1H, N<u>H</u>CH), 7.51 (d, *J* = 9.0 Hz, 1H, H_{Py}), 7.37 (bs, 1H, NH), 6.92–6.88 (m, 4H, H_{Ar}), 6.47 (bs, 1H, NH), 4.62 (q, *J* = 7.5 Hz, 1H, $CHCF_3$), 4.51–4.47 (m, 4H, CH_2N), 4.11–4.07 (m, 4H, CH_2O), 3.85 (s, 2H, CH_2Py), 3.83 (s, 4H, 2 × CH_2-triazole), 3.82–3.77 (m, 4H, CH_2O), 3.74–3.69 (m, 4H, CH_2O), 3.54–3.50 (m, 8H, 4 × CH_2O), 1.52 (s, 3H, CH_3), 1.51–1.50 (m, 6H, 2 × CH_3), 1.49 (s, 3H, CH_3), 1.46 (s, 3H, CH_3), 1.44 (s, 3H, CH_3), 1.40 (s, 3H, CH_3), 1.37 (s, 3H, CH_3), 1.32 (s, 3H, CH_3).

^{19}F NMR (377 MHz, $CDCl_3$): δ –77.3 (s, 3F, CF_3).

^{13}C NMR (101 MHz, $CDCl_3$): δ 175.8, 174.5, 174.1, 173.6, 166.4, 163.8, 148.7, 144.5, 135.7, 127.1 (q, *J* = 289Hz, <u>C</u>F_3), 124.6, 122.1, 114.8, 70.6, 70.4, 69.8, 69.5, 68.8, 59.3, 57.6, 57.13, 57.03, 56.9, 50.3, 49.5, 46.3 (q, *J* = 31 Hz, <u>C</u>HCF_3), 29.8, 27.4, 26.8, 26.0, 25.8, 23.7, 23.3, 13.5.

HRMS (ES^+, MeOH): m/z calculated for $C_{50}H_{73}O_{11}N_{13}F_3$ $[M+H]^+$ 1088.5499, found 1088.5495.

2.2.4 Compound (*R*)-6

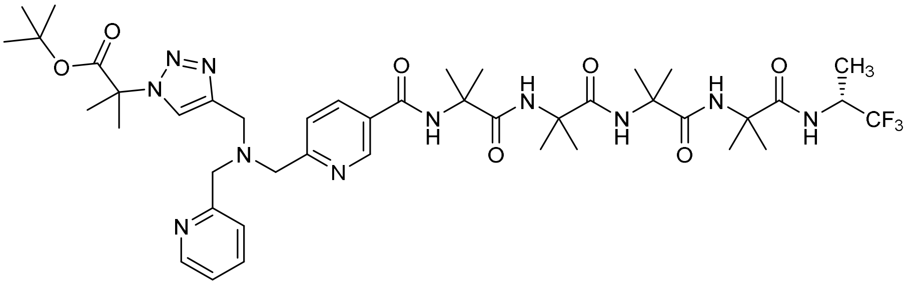

$CuSO_4 \cdot 5H_2O$ (7 mg, 0.03 mmol), sodium ascorbate (11 mg, 0.06 mmol) and alkyne (*R*)-**11** (102 mg, 0.14 mmol) were dissolved in dry DMF (3 mL) under Ar. N_3AibO*t*Bu[S5] (32 mg, 0.17 mmol) was added to the reaction mixture and stirred at rt overnight. The solvent was then evaporated under high vacuum. The residue was re-dissolved in $CHCl_3$ (10 mL) and washed with saturated EDTA solution (2 mL). The aqueous phase was re-extracted with $CHCl_3$ (3 × 2 mL). The combined organic layers were dried over Na_2SO_4, filtered and evaporated under reduced pressure. The crude was purified by flash chromatography (SiO_2, CH_2Cl_2/CH_3CN/MeOH 5:5:1 to 0:0:1) to afford the title compound as a brown oil (47 mg, 37%).

^{1}H NMR (400 MHz, $CDCl_3$): δ 9.03 (s, 1H, H_{Py}), 8.49 (t, *J* = 4.1 Hz, 1H, H_{Py}), 8.18 (dd, *J* = 7.2, 3.5 Hz, 1H, H_{Py}), 7.73 (bs, 1H, NH), 7.69 (s, 1H, $H_{triazole}$), 7.68–7.55 (m, 5H, 3 × H_{Py}, 2 × NH), 7.49 (bs, 1H, NH), 7.14 (m, 1H, H_{Py}), 6.86 (bs, 1H, NH), 4.59–4.47 (m, 1H, $CHCF_3$), 3.86 (s, 2H, CH_2), 3.82 (s, 2H, CH_2), 3.79 (s, 2H, CH_2), 1.87 (s, 6H, 2 × CH_3), 1.56 (s, 6H, 2 × CH_3), 1.52–1.48 (m, 6H, 2 × CH_3), 1.45 (m, 9H, 3 × CH_3), 1.36 (m, 15H, 5 × CH_3).

^{19}F NMR (377 MHz, $CDCl_3$): δ – 77.2 (s, 3F, CF_3).

^{13}C NMR (101 MHz, $CDCl_3$): δ 176.1, 170.5, 166.8, 166.5, 158.8, 149.4, 148.6, 143.3, 136.8, 135.5, 123.4, 122.9, 122.4, 122.1, 83.1, 65.1, 59.4, 59.2, 57.7, 57.1, 57.0, 56.9, 48.5, 46.4, 45.4, 45.2, 27.8, 27.3, 27.1, 26.5, 25.7, 24.1, 24.0, 23.9, 23.6, 13.4.

HRMS (ES^+, MeOH): m/z calculated for $C_{43}H_{62}O_7N_{11}F_3Na$ $[M+Na]^+$ 924.4683, found 924.5121.

2.2.5 Compound S14

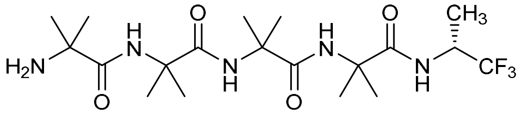

Compound **S13**[S5] (598 mg, 1.35 mmol) was dissolved in EtOH (50 mL). Pd/C (200 mg, 10%) was slowly added and the reaction mixture was vigorously stirred under H_2 atmosphere until NMR indicated consumption of the starting material. The mixture was then washed through a pad of Celite® with EtOAc and the filtrate was concentrated under reduced pressure to give the title compound as a white foam (582 mg, quantitative yield) which was used without further purification.

^{1}H NMR (400 MHz, $CDCl_3$): δ 8.18 (bs, 1H, NH), 7.53 (bs, 1H, NH), 7.44 (d, *J* = 9.1 Hz, 1H, N*H*CH), 6.26 (bs, 1H, NH), 4.70–4.56 (m, 1H, NHC*H*), 1.53 (s, 3H, CH_3), 1.48 (s, 3H, CH_3), 1.46 (s, 3H, CH_3), 1.43 (s, 3H, CH_3), 1.39–1.34 (m, 12H, 4 × CH_3), 1.28–1.23 (m, 3H, CH_3).

^{19}F NMR (377 MHz, $CDCl_3$): δ –77.3.

^{13}C NMR (101 MHz, $CDCl_3$): δ 178.6, 175.6, 173.9, 57.2, 56.8, 56.5, 54.9, 29.8, 29.1, 28.9, 27.1, 26.9, 25.9, 14.3, 13.6.

HRMS (ES$^+$, MeOH): m/z calculated for $C_{19}H_{34}O_4N_5F_3Na$ $[M+Na]^+$ 476.2455, found 476.2438.

2.2.6 Compound S16

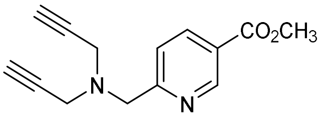

Under N_2, DIPEA (1.0 mL, 5.92 mmol) was added to a solution of dipropargylamine (0.4 mL, 3.95 mmol) and methyl 6-(bromomethyl)nicotinate (1.00 g, 4.34 mmol) in dry CH_3CN (23.0 mL). The mixture was stirred overnight at rt. After solvent evaporation under reduced pressure, the residue was re-dissolved in CH_2Cl_2 (20 mL) and washed with brine (20 mL). The aqueous phase was re-extracted with CH_2Cl_2 (2 × 5 mL). The combined organic layers were dried over $MgSO_4$, filtered and evaporated under reduced pressure to afford the title compound as a brown oil (1.04 g, quantitative yield).

^{1}H NMR (400 MHz, $CDCl_3$): δ 9.15 (d, *J* = 1.6 Hz, 1H, H_{Py}), 8.27 (dd, *J* = 8.1, 2.1 Hz, 1H, H_{Py}), 7.57 (d, *J* = 8.1 Hz, 1H, H_{Py}), 3.93 (s, 3H, OCH_3), 3.92 (s, 2H, CH_2Py), 3.49 (d, *J* = 2.3 Hz, 4H, 2 × CH_2C_{sp}), 2.27 (t, *J* = 2.3 Hz, 2H, 2 × $C_{sp}H$).

^{13}C NMR (101 MHz, $CDCl_3$): δ 165.9, 162.9, 150.7, 137.9, 124.9, 122.8, 78.5, 73.7, 58.8, 52.5, 42.6.

HRMS (ES$^+$, MeOH): m/z calculated for $C_{14}H_{14}O_2N_2K$ $[M+K]^+$ 281.0687, found 281.0676.

2.2.7 Compound S17

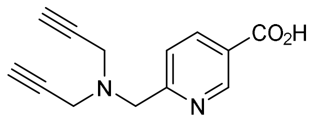

To a solution of compound **S16** (170 mg, 0.70 mmol) in MeOH (15 mL), KOH (186 mg, 3.31 mmol) was added. The mixture was stirred for 3 h at reflux. Then, the mixture was left to cool to rt and neutralized with HCl until pH 5–6 and evaporated under reduced pressure. The resulting solid was dissolved in $CHCl_3$ (20 mL) and brine (10 mL). The organic phase was separated and the aqueous phase was further extracted with $CHCl_3$ (2 × 20 mL). The combined organic phase was then dried over $MgSO_4$, filtered and evaporated under reduced pressure to afford the title compound as a dark yellow solid (137 mg, 86%).

^{1}H NMR (400 MHz, CD_3OD): δ 9.08 (d, *J* = 1.7 Hz, 1H, H_{Py}), 8.40 (dd, *J* = 8.1, 2.0 Hz, 1H, H_{Py}), 7.68 (d, *J* = 8.1 Hz, 1H, H_{Py}), 4.04 (s, 2H, CH_2N), 3.59 (d, *J* = 2.4 Hz, 4H, 2 × CH_2C_{sp}), 2.76 (t, *J* = 2.3 Hz, 2H, 2 × $C_{sp}H$).

^{13}C NMR (101 MHz, CD_3OD): δ 167.6, 162.7, 150.9, 139.9, 127.5, 124.6, 78.4, 75.8, 58.8, 43.2.

HRMS (ES$^+$, MeOH): m/z calculated for $C_{13}H_{11}O_2N_2$ [M–H]$^-$ 227.0826, found 227.0825.

2.2.8 Compound (*R*)-7

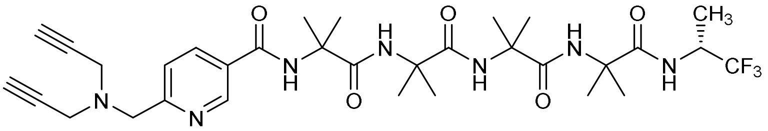

Under N_2, acid **S17** (344 mg, 0.61 mmol), amine **S14** (181 mg, 1.21 mmol) and HATU (253 mg, 0.66 mmol) were dissolved in dry CH_2Cl_2 (8 mL). Then, DIPEA (0.3 mL, 1.82 mmol) was added and the reaction mixture was stirred for 3 days at rt. The mixture was then diluted with CH_2Cl_2 (20 mL) and washed with sat. $NaHCO_3$ solution (20 mL). The aqueous phase was re-extracted with CH_2Cl_2 (2 × 20 mL). The combined organic layers were dried over $MgSO_4$, filtered and evaporated under reduced pressure. The crude product was then purified by flash chromatography (SiO_2, CH_2Cl_2/CH_3CN 1:1) to give the title compound as a white solid (218 mg, 54%).

^{1}H NMR (400 MHz, $CDCl_3$): δ 9.03 (d, *J* = 2.2 Hz, 1H, H_{Py}), 8.22 (bs, 1H, NH), 8.17 (dd, *J* = 8.1, 2.3 Hz, 1H, H_{Py}), 7.74 (bs, 1H, NH), 7.62 (bs, 1H, NH), 7.59 (d, *J* = 9.0 Hz, 1H, N*H*CH), 7.53 (d, *J* = 8.2 Hz, 1H, H_{Py}), 6.80 (bs, 1H, NH), 4.44 (m, 1H, $CHCF_3$), 3.87 (s, 2H, CH_2Py), 3.43 (d, *J* = 2.4 Hz, 4H, 2 × CH_2C_{sp}), 2.25 (t, *J* = 2.4 Hz, 2H, 2 × $C_{sp}H$), 1.54 (s, 6H, 2 × CH_3), 1.47 (s, 3H, CH_3), 1.44 (s, 3H, CH_3), 1.42 (s, 3H, CH_3), 1.41 (s, 3H, CH_3), 1.39 (s, 3H, CH_3), 1.33–1.29 (m, 6H, 2 × CH_3).

^{19}F NMR (377 MHz, $CDCl_3$): δ –77.3 (s, 3F, CF_3).

^{13}C NMR (101 MHz, $CDCl_3$): δ 176.3, 174.9, 174.9, 174.4, 166.6, 165.7, 162.2, 148.9, 135.9, 127.7, 125.7 (q, *J* = 282 Hz, CF_3), 122.8, 78.4, 73.6, 58.6, 57.6, 57.0, 56.8, 56.7, 46.6 (q, *J* = 31 Hz, *C*HCF_3), 42.4, 27.0, 26.4, 25.6, 24.0, 23.5, 13.3.

HRMS (ES$^+$, MeOH): m/z calculated for $C_{32}H_{44}O_5N_7F_3Na$ [M+Na]$^+$ 686.3248, found 686.3233.

2.2.9 Compound S18

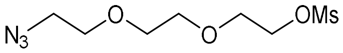

A solution of 8-azido-3,6-dioxaoctanol (2-[2-(2-azidoethoxy)ethoxy]ethanol, 1.18 g, 6.76 mmol) and TEA (1.9 mL, 13.5 mmol) in CH_2Cl_2 (30 mL) was cooled at 0°C. Mesyl chloride (1.0 mL, 13.5 mmol,) was added dropwise and the mixture was stirred for an additional 10 min at 0°C. The mixture was then left to warm to rt and further stirred for 25 h. Then, the solvent was evaporated under reduced pressure and the crude product was purified by flash chromatography (SiO_2, hexane/EtOAc 1:0 to 1:1) to afford the title compound (1.66 g, 97%) as a pale yellow oil.

^{1}H NMR (400 MHz, $CDCl_3$): δ 4.41–4.33 (m, 2H, CH_2O), 3.81–3.73 (m, 2H, CH_2O), 3.72–3.61 (m, 6H, 3 × CH_2O), 3.38 (t, J = 5.0 Hz, 2H, CH_2O), 3.07 (s, 3H, CH_3).

^{13}C NMR (101 MHz, $CDCl_3$): δ 70.8, 70.8, 70.2, 69.3, 69.2, 50.8, 37.8.

HRMS (ES$^+$, MeOH): m/z calculated for $C_7H_{16}O_5N_3S$ [M+H]$^+$ 254.0805, found 254.0802.

2.2.10 Compound (*R*)-8

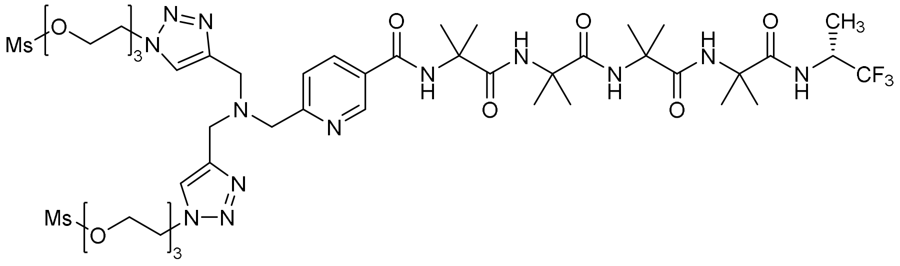

$CuSO_4{\cdot}5H_2O$ (34 mg, 0.14 mmol) and sodium ascorbate (54 mg, 0.14 mmol) were added to a solution of bis-alkyne (*R*)-**7** (451 mg, 0.68 mmol) and azide **S18** (428 mg, 1.69 mmol) in dry DMF (14 mL). The mixture was stirred under Ar for 5 h. The solvent was then evaporated under high vacuum. The residue was re-dissolved in $CHCl_3$ (10 mL) and washed with saturated EDTA solution (2 mL). The aqueous phase was re-extracted with $CHCl_3$ (3 × 5 mL). The organic phase was dried over $MgSO_4$, filtered and evaporated under reduced pressure. The crude was purified by flash chromatography (SiO_2, CH_2Cl_2/CH_3CN/MeOH 5:5:1) to afford the title compound as a yellow oil (422 mg, 53%).

^{1}H NMR (400 MHz, $CDCl_3$): δ 9.02 (d, J = 2.3 Hz, 1H, H_{Py}), 8.15 (dd, J = 8.1, 2.3 Hz, 1H, H_{Py}), 7.81 (s, 2H, 2 × $H_{triazole}$), 7.69 (bs, 2H, 2 × NH), 7.55 (d, J = 8.1 Hz, 1H, H_{Py}), 7.51 (bs, 1H, NH), 6.72 (bs, 1H, NH), 4.66–4.57 (m, 1H, CH), 4.57–4.50 (m, 4H, 2 × CH_2O), 4.30–4.29 (m, 4H, 2 × CH_2O), 3.90–3.82 (m, 6H, 2 × CH_2O, CH_2Py), 3.79 (s, 4H, 2 × CH_2-triazole), 3.72–3.66 (m, 4H, 2 × CH_2O), 3.64–3.56 (m, 8H, 4 × CH_2O), 2.99 (s, 6H, 2 × CH_3), 1.56 (s, 6H, 2 × CH_3), 1.51 (s, 6H, 2 × CH_3), 1.48 (s, 6H, 2 × CH_3), 1.44 (s, 3H, CH_3), 1.37 (d, J = 7.1 Hz, 3H, CH_3CH), 1.35 (s, 3H, CH_3).

^{19}F NMR (377 MHz, $CDCl_3$): δ –77.3 (s, 3F, CF_3).

^{13}C NMR (101 MHz, $CDCl_3$): δ 175.9, 174.7, 174.2, 173.9, 166.3, 163.2, 148.3, 143.8, 136.0, 127.6, 125.5 (q, J_{C-F} = 293 Hz), 123.4, 70.7, 70.6, 69.6, 69.5, 69.1, 58.9, 57.7, 57.1, 56.9, 50.4, 47.9, 46.6 (q, J_{C-F} = 30 Hz), 37.8, 27.2, 26.6, 25.9, 24.1, 23.8, 21.9, 13.5.

HRMS (ES$^+$, MeOH): m/z calculated for $C_{46}H_{74}O_{15}N_{13}F_3NaS_2$ [M+Na]$^+$ 1192.4713, found 1192.4684.

2.2.11 Compound 10

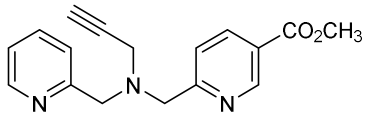

Under N_2, DIPEA (0.9 mL, 5.06 mmol) was added to a solution of amine **9**[S3] (493 mg, 3.37 mmol) and methyl 6-(bromomethyl)nicotinate **S15** (776 g, 3.37 mmol) in dry CH_3CN (18 mL). The mixture was stirred for 2 days at rt. After solvent evaporation under reduced pressure, the residue was purified by flash chromatography (SiO_2, CH_2Cl_2/MeOH 20:1) to afford the title compound as a dark orange oil (611 mg, 61%).

^{1}H NMR (400 MHz, $CDCl_3$): δ 9.14 (dd, *J* = 2.2, 0.8 Hz, 1H, H_{Py}), 8.58 (ddd, *J* = 4.9, 1.8, 0.9 Hz, 1H, H_{Py}), 8.26 (dd, *J* = 8.1, 2.2 Hz, 1H, H_{Py}), 7.70 (td, *J* = 7.7, 2.0 Hz, 1H, H_{Py}), 7.65 (d, *J* = 8.2 Hz, 1H, H_{Py}), 7.53 (d, *J* = 7.8 Hz, 1H, H_{Py}), 7.24–7.17 (m, 1H, H_{Py}), 4.00 (s, 2H, CH_2), 3.96 (s, 2H, CH_2), 3.94 (s, 3H, CH_3), 3.45 (d, *J* = 2.4 Hz, 2H, CH_2C_{sp}), 2.31 (t, *J* = 2.4 Hz, 1H, $C_{sp}H$).

^{13}C NMR (101 MHz, $CDCl_3$): δ 165.9, 163.5, 158.2, 150.6, 149.0, 137.8, 137.1, 124.8, 123.6, 122.8, 122.6, 78.1, 74.1, 59.5, 59.4, 52.5, 43.0.

HRMS (ES$^+$, MeOH): m/z calculated for $C_{17}H_{17}O_2N_3Na$ [M+Na]$^+$ 318.1213, found 318.1205.

2.2.12 Compound S19

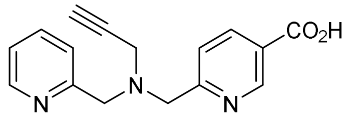

To a solution of compound **10** (611 mg, 2.07 mmol) in MeOH (45 mL), KOH (472 mg, 8.42 mmol) was added. The mixture was stirred for 3 h at reflux. Then, the mixture was left to cool to rt and neutralized with HCl until pH 5–6 and evaporated under reduced pressure. The resulting solid was dissolved in $CHCl_3$ (20 mL) and brine (10 mL). The organic phase was separated and the aqueous phase was further extracted with $CHCl_3$ (2 × 20 mL). The combined organic phase was then dried over $MgSO_4$, filtered and evaporated under reduced pressure to afford the title compound as a dark solid (600 mg, quant.).

^{1}H NMR (400 MHz, CD_3OD): δ 9.04 (dd, *J* = 2.2, 0.9 Hz, 1H, H_{Py}), 8.50 (ddd, *J* = 5.1, 1.8, 0.9 Hz, 1H, H_{Py}), 8.34 (dd, *J* = 8.1, 2.2 Hz, 1H, H_{Py}), 7.92–7.83 (m, 1H, H_{Py}), 7.73 (dd, *J* = 8.1, 0.8 Hz, 1H, H_{Py}), 7.67 (dt, *J* = 7.9, 1.1 Hz, 1H, H_{Py}), 7.37 (ddd, *J* = 7.5, 5.1, 1.2 Hz, 1H, H_{Py}), 4.01 (s, 2H, CH_2), 3.98 (s, 2H, CH_2), 3.45 (d, *J* = 2.4 Hz, 2H, CH_2C_{sp}), 2.73 (t, *J* = 2.4 Hz, 1H, $C_{sp}H$).

^{13}C NMR (101 MHz, CD_3OD): δ 167.9, 163.8, 159.1, 151.0, 149.0, 139.7, 139.6, 127.5, 125.3, 124.5, 124.4, 78.4, 75.7, 59.9, 59.8, 43.6.

HRMS (ES$^+$, MeOH): m/z calculated for $C_{16}H_{14}O_2N_3$ [M–H]$^-$ 280.1092, found 280.1092.

2.2.13 Compound (*R*)-11

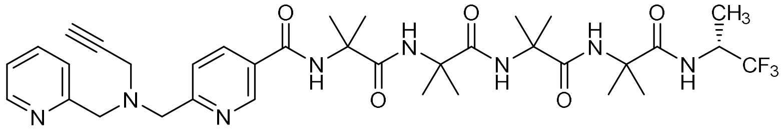

A solution of acid **S19** (183 mg, 0.65 mmol), amine (*R*)-**S14** (236 mg, 0.52 mmol) and HOBt (88 mg, 0.65 mmol) in CH_3CN (15 mL) was cooled at 0 °C. Then, EDC·HCl was added and the mixture was left to warm to rt followed by addition of TEA (0.1 mL, 0.65 mmol). The reaction mixture was further stirred at rt for 3 days. The mixture was then diluted with CH_2Cl_2 (100 mL) and washed with H_2O (2 × 20 mL). The organic phase was dried over $MgSO_4$, filtered and evaporated under reduced pressure. The crude product was then purified by flash chromatography (SiO_2, $CH_2Cl_2/CH_3CN/CH_3OH$ 1:1:0 to 1:1:0.1) to give the title compound as a white solid (204 mg, 55%).

^{1}H NMR (400 MHz, $CDCl_3$): δ 9.04 (d, *J* = 2.2 Hz, 1H, H_{Py}), 8.53 (dd, *J* = 4.7, 1.9 Hz, 1H, H_{Py}), 8.17 (dd, *J* = 8.2, 2.3 Hz, 1H, H_{Py}), 7.80 (bs,1H, NH), 7.72 (bs, 1H, NH), 7.68–7.62 (d, *J* = 7.9 Hz, 2H, 2 × H_{Py}), 7.59 (bs, 2H, 2 × NH), 7.47 (d, *J* = 7.8 Hz, 1H, H_{Py}), 7.15 (ddd, *J* = 7.6, 4.9, 1.3 Hz, 1H, H_{Py}), 6.65 (bs,, 1H, NH), 4.52–4.41 (m, 1H, $CHCF_3$), 3.96 (s, 2H, CH_2), 3.89 (s, 2H, CH_2), 3.38 (d, *J* = 2.5 Hz, 2H, CH_2C_{sp}), 2.29 (t, *J* = 2.3 Hz, 1H, $C_{sp}H$), 1.56 (d, *J* = 2.6 Hz, 6H, 2 × CH_3), 1.49 (s, 3H, CH_3), 1.47 (s, 3H, CH_3), 1.43 (s, 6H, 2 × CH_3), 1.41 (s, 3H, CH_3), 1.34 (s, 3H, CH_3), 1.33 (d, *J* = 6.9 Hz, 3H, $CHCH_3$).

^{19}F NMR (377 MHz, $CDCl_3$): δ –77.2 (s, 3F, CF_3).

^{13}C NMR (101 MHz, $CDCl_3$): δ 176.2, 174.8, 174.5, 174.1, 166.5, 163.1, 158.4, 149.5, 148.7, 136.8, 135.9, 127.5, 123.3, 122.9, 122.5, 116.6, 74.1, 59.5, 59.2, 57.7, 57.1, 56.9, 56.9, 42.7, 27.1, 26.5, 25.8, 24.1, 23.9, 23.5, 13.4.

HRMS (ES$^+$, MeOH): m/z calculated for $C_{35}H_{47}O_5N_8F_3Na$ $[M+Na]^+$ 739.3514, found 739.3484.

2.2.14 Compound S20

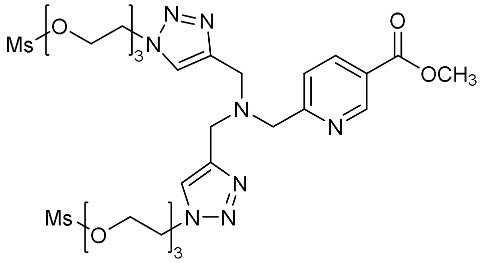

$CuSO_4$·$5H_2O$ (33 mg, 0.13 mmol) and sodium ascorbate (52 mg, 0.26 mmol) were added to a solution of bis-alkyne **S16** (160 mg, 0.66 mmol) and azide **S18** (351 mg, 1.38 mmol) in dry DMF (8 mL). The mixture was stirred under Ar for 5 h. The solvent was then evaporated under high vacuum. The residue was re-dissolved in $CHCl_3$ (10 mL) and washed with saturated EDTA solution (2 mL). The aqueous phase was re-extracted with $CHCl_3$ (3 × 2 mL). The organic phase was dried over $MgSO_4$, filtered and evaporated under reduced pressure. The crude was purified by flash chromatography (SiO_2, $CH_2Cl_2/CH_3CN/MeOH$ 5:5:1 to 0:0:1) to afford the title compound as a yellow oil (237 mg, 48%).

^{1}H NMR (400 MHz, $CDCl_3$): δ 9.16–9.10 (m, 1H, H_{Py}), 8.30–8.22 (m, 1H, H_{Py}), 7.81 (s, 2H, 2 × $H_{triazole}$), 7.74–7.66 (m, 1H, H_{Py}), 4.57–4.49 (m, 4H, 2 × CH_2O), 4.37–4.30 (m, 4H, 2 × CH_2O), 3.96–3.92 (m, 3H, CH_3), 3.91–3.85 (m, 6H, 3 × CH_2), 3.82 (s, 4H, 2 × CH_2), 3.75–3.68 (m, 4H, 2 × CH_2O), 3.66–3.56 (m, 4H, 2 × CH_2O), 3.03 (s, 6H, 2 × OMs).

^{13}C NMR (101 MHz, $CDCl_3$): δ 165.9, 163.8, 150.4, 150.3, 143.7, 137.6, 137.6, 124.6, 123.0, 122.8, 70.6, 69.5, 69.2, 69.1, 59.4, 58.9, 52.4, 50.2, 47.8, 37.6.

HRMS (ES^+, MeOH): m/z calculated for $C_{28}H_{44}O_{12}N_8NaS_2$ $[M+Na]^+$ 771.2418, found 771.2399.

2.2.15 Compound S21

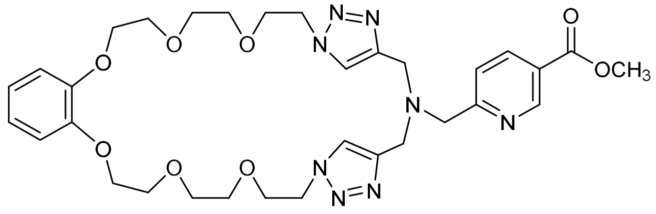

Under N_2, catechol (17 mg, 0.16 mmol) and Cs_2CO_3 (206 mg, 0.63 mmol) were dissolved in degassed CH_3CN (60 mL). Then, a solution of compound **S20** (118 mg, 0.16 mmol) in degassed CH_3CN (3 mL) was added and the reaction mixture was heated to reflux for 3 days. The mixture was then left to cool at rt, evaporated under reduced pressure and re-dissolved in EtOAc (20 mL). The organic layer was washed with brine (10 mL) and the aqueous layer was re-extracted with CH_2Cl_2 (3 × 10 mL). The combined organic layers were dried over $MgSO_4$, filtered and evaporated under reduced pressure. The crude was purified by flash chromatography (SiO_2, $CH_2Cl_2/CH_3CN/CH_3OH$ 5:5:1 to 0:0:1) to afford the title compound as a yellow oil (78 mg, 74%).

^{1}H NMR (400.1 MHz, $CDCl_3$): δ 9.10 (d, J = 2.1 Hz, 1H, H_{Py}), 8.21 (dd, J = 8.1, 2.3 Hz, 1H, H_{Py}), 7.84 (s, 2H, $H_{triazole}$), 7.67 (d, J = 8.2 Hz, 1H, H_{Py}), 6.88–6,81 (m, 4H, H_{Ar}), 4.54–4.48 (m, 4H, 2 × CH_2O), 4.10–4.04 (m, 4H, 2 × CH_2O), 3.91 (s, 3H, CH_3), 3.87–3.83 (m, 4H, 2 × CH_2O + CH_2-triazole), 3.79–3.71 (m, 8H, 2 × CH_2O + 2 × CH_2Py), 3.67–3.56 (m, 8H, 4 × CH_2O) ppm.

^{13}C NMR (100.6 MHz, $CDCl_3$): δ 165.9, 164.0, 150.3, 148.8, 144.3, 137.6, 124.5, 124.4, 122.8, 121.68, 114.7, 70.7, 70.7, 69.8, 69.6, 68.8, 59.5, 52.3, 50.3, 48.1 ppm.

HRMS (ES^+, MeOH): m/z calculated for $C_{32}H_{43}O_8N_8$ $[M+H]^+$ 667.3198, found 667.3198.

3. Synthesis of Zn(II) perchlorate foldamer complexes

3.1 General procedure

The preparation of Zn((*S*)-**3**)·2ClO$_4$, Zn((*R*)-**4**)·2ClO$_4$, Zn((*R*)-**5**)·2ClO$_4$, Zn((*R*)-**6**)·2ClO$_4$ and Zn(**S21**)·2ClO$_4$ was adapted from a reported procedure.[S6] Compounds (*S*)-**3**, (*R*)-**4**, (*R*)-**5**, (*R*)-**6** or **S21** (0.63 mmol) were dissolved in CH_3OH (18 mL). $Zn(ClO_4)_2·6H_2O$ (0.63 mmol) was added dropwise as a solution in MeOH (9 mL) and the resulting solution was stirred at rt for 10 min. Diethyl ether (55 mL) was added dropwise and the suspension was stirred for 1 h. The suspension was filtered to give the title compound. Yields are from 87% to 98%.

3.2 Compound Zn((*S*)-3)·2ClO$_4$

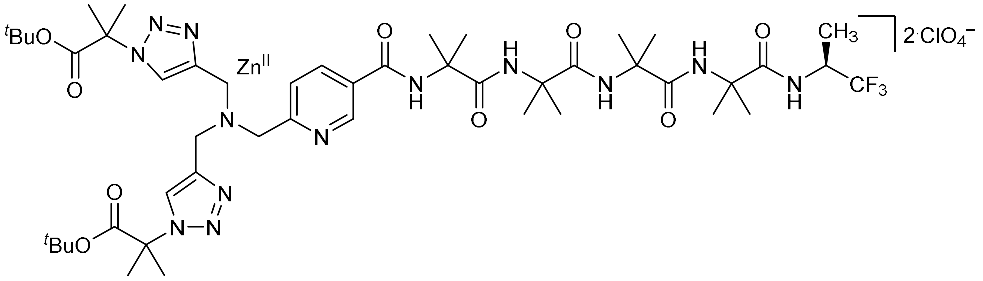

(*S*)-**3** was acquired in reasonable yields before complexing with one equivalent of zinc(II) in acetonitrile to arrive at the novel receptor Zn((*S*)-**3**). To a solution of (*S*)-**3** (50 mg, 0.048 mmol) in CH_3CN, $Zn(ClO_4)_2·6H_2O$ (18 mg, 0.048 mmol) was added. Upon solvation, the solvent was evaporated under reduced pressure, to afford the title compound as a pale yellow solid (62 mg, quant. yield).

^{1}H NMR (400 MHz, CD_3CN): δ 9.09 (m, 1H, H_{Py}), 8.60 (dd, *J* = 8.3, 1.9 Hz,1H, H_{Py}), 8.15 (s, 2H, 2 × $H_{triazole}$), 7.93 (s, 1H, NH), 7.77 (d, *J* = 8.3 Hz ,1H, H_{Py}), 7.60-7.58 (m, 2H, NH + N*H*CHCF$_3$), 7.53 (s, 1H, NH), 7.25 (s, 1H, NH), 4.56 (m, 1H, CF$_3$C*H*), 4.38 (s, 2H, CH$_2$), 4.20 (s, 4H, 2 × CH$_2$), 1.89 (s, 12H, 2 × (CH$_3$)$_2$), 1.55 (s, 3H, CH$_3$), 1.54 (s, 3H, CH$_3$), 1.46 (s, 3H, CH$_3$), 1.43 (s, 3H, CH$_3$), 1.40 (m, 6H, 2 × CH$_3$), 1.38 (s, 18H, 2 × C(CH$_3$)$_3$), 1.37 (s, 3H, CH$_3$), 1.33-1.32 (m, 6H, 2 × CH$_3$) ppm;

^{13}C NMR (101 MHz, $CDCl_3$): δ 176.0 (CO), 175.8 (CO), 174.8 (CO), 174.6 (CO), 169.9 (CO), 164.4 (CO), 159.2 (ArC$_q$), 148.6 (ArCH$_{py}$), 144.9 (ArC$_q$), 141.7 (ArCH$_{py}$), 131.9 (ArC$_q$), 126.6 (q, *J* = 280.0 Hz, CF$_3$), 125.9 (ArCH$_{py}$), 124.4 (ArCH$_{triazole}$), 84.2 (*C*(CH$_3$)$_3$), 68.3 (triazole-*C*(CH$_3$)$_2$), 58.2 (C$_q$), 57.7 (CH$_2$), 57.2 (C$_q$), 57.1 (C$_q$), 57.0 (C$_q$), 49.8 (2 × CH$_2$), 46.7 (q, *J* = 32.0 Hz, *C*HCF$_3$), 27.3 (2 × (CH$_3$)$_3$), 26.4 (CH$_3$), 25.9 (CH$_3$), 25.5 (CH$_3$), 25.0 (d, *J* = 5.0 Hz, 2 × triazole-(C(*C*H$_3$)$_2$)$_2$), 25.0 (CH$_3$), 24.4 (CH$_3$), 24.1 (CH$_3$), 24.1 (CH$_3$), 23.8 (CH$_3$), 13.5 (NHCH*C*H$_3$) ppm;

^{19}F NMR (376 MHz, CD_3CN): δ -77.6 (d, *J* =7.2 Hz, 3F, CF$_3$) ppm;

HRMS (ESI$^+$): *m/z* calcd. for $C_{48}H_{74}O_{17}N_{13}Cl_2F_3NaZn$ ([M+Na]$^+$): 1318.3838, found 1318.3836.

3.3 Compound Zn((*R*)-4)·2ClO4

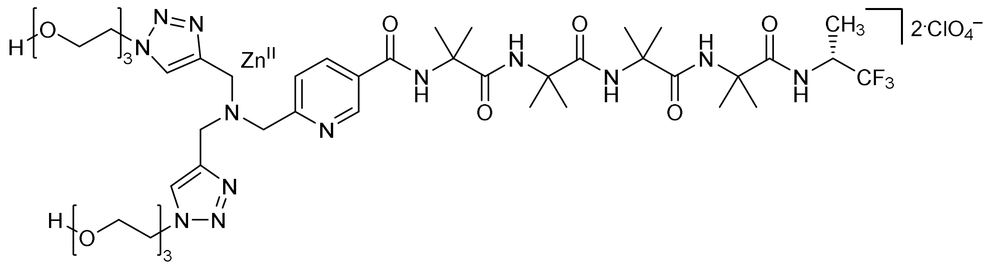

^{1}H NMR (400 MHz, CD_3CN): δ 9.76–9.03 (m, 1H, H_{Py}), 8.56 (s, 1H, HPy), 8.13 (s, 2H, 2 × $H_{triazole}$), 7.92 (bs, 1H, NH), 7.73 (bs, 1H, NH), 7.66–7.46 (m, 3H, 2 × NH, H_{Py}), 7.24 (bs, 1H, NH), 4.65–4.59 (m, 4H, 2 × CH_2O), 4.60–4.50 (m, 1H, C*H*CF3), 4.32 (bs, 2H, CH_2Py), 4.15 (bs, 4H, 2 × CH_2-triazole) , 3.91–3.83 (m, 4H, 2 × CH_2O), 3.61–3.55 (m, 4H, 2 × CH_2O), 3.54–3.48 (m, 8H, 4 × CH_2O), 1.54 (s, 3H, CH_3), 1.52 (s, 3H, CH_3), 1.46 (s, 3H, CH_3), 1.43 (s, 3H, CH_3), 1.39 (s, 6H, 2 × CH_3), 1.36 (s, 3H, CH_3), 1.33–1.32 (m, 6H, 2 × CH_3).

^{19}F NMR (377 MHz, CD_3CN): δ –77.5 (s, 3F, CF_3).

^{13}C NMR (101 MHz, CD_3CN): δ 176.5, 176.3, 175.3, 175.1, 159.9, 149.3, 145.6, 126.1, 73.1, 70.9, 70.7, 69.2, 66.3, 61.9, 58.6, 57.7, 57.6, 57.4, 52.8, 47.3, 47.0, 26.9, 26.4, 26.0, 25.5, 24.7, 24.6, 24.2, 15.6, 13.9, 13.9.

HRMS (ES$^+$, MeOH): m/z calculated for $C_{44}H_{70}O_{11}N_{13}F_3Zn$ $[M+Zn]^{2+}$ 538.7275, found 538.7300.

3.4 Compound Zn((*R*)-5)·2ClO4

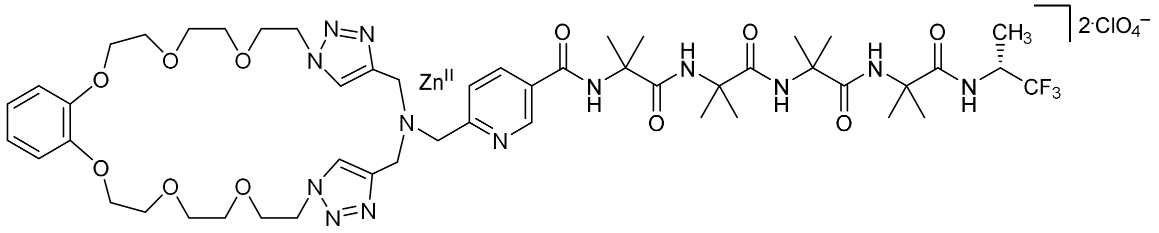

^{1}H NMR (400 MHz, $CDCl_3$): δ 9.06 (s, 1H, H_{Py}), 8.57 (d, *J* = 8.4 Hz, 1H, H_{Py}), 8.14 (s, 2H, 2 × $H_{triazole}$), 7.88 (bs, 1H, NH), 7.72 (d, *J* = 8.2 Hz, 1H, H_{Py}), 7.62–7.48 (m, 3H, 3 × NH), 7.21 (bs, 1H, NH), 7.08–6.79 (m, 4H, H_{Ar}), 4.68–4.51 (m, 5H, 2 × CH_2, $CHCF_3$), 4.19–3.96 (m, 6H, 3 × CH_2), 3.93–3.74 (m, 6H, 3 × CH_2), 3.70–3.62 (m, 4H, 2 × CH_2), 3.59–3.48 (m, 8H, 4 × CH_2), 1.58 – 1.52 (m, 6H, 2 × CH_3), 1.47–1.41 (m, 6H, 2 × CH_3), 1.40–1.36 (m, 9H, 3 × CH_3), 1.35–1.31 (m, 6H, 2 × CH_3).

^{19}F NMR (377 MHz, $CDCl_3$): δ –78.2 (s, 3F, CF_3).

^{13}C NMR (101 MHz, $CDCl_3$): δ 176.4, 176.2, 175.3, 175.1, 149.7, 145.1, 136.6, 126.3, 122.7, 118.3, 115.4, 70.9, 70.7, 70.3, 69.7, 69.1, 66.3, 58.6, 57.7, 57.6, 57.5, 52.8, 50.3, 47.0, 26.9, 26.4, 25.9, 25.4, 24.6, 24.2, 15.6, 13.9, 1.9, 1.7.

HRMS (ES$^+$, MeOH): m/z calculated for $C_{50}H_{72}O_{11}N_{13}F_3Zn$ $[M+Zn]^{2+}$ 575.7353, found 575.7369.

3.5 Compound Zn(S21)·2ClO$_4$

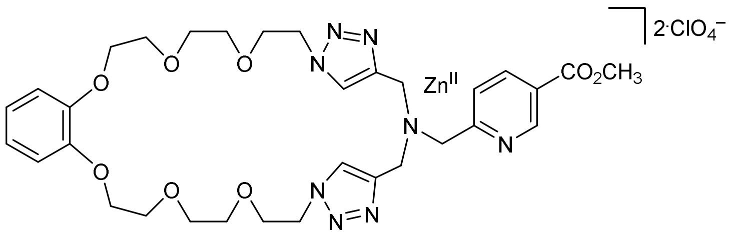

^{1}H NMR (400 MHz, CD_3CN): δ 9.27 (s, 1H), 8.56 (d, *J* = 2.1 Hz, 0H), 8.12 (s, 1H), 7.68 (d, *J* = 8.3 Hz, 1H), 7.13–6.72 (m, 3H), 4.72–4.46 (m, 3H), 4.19–3.98 (m, 2H), 3.94 (s, 2H), 3.92–3.83 (m, 1H), 3.82–3.71 (m, 1H), 3.70–3.44 (m, 5H), 1.96 (s, 2H).

^{13}C NMR (101 MHz, CD_3CN): δ 164.9, 160.8, 149.7, 145.1, 142.8, 128.9, 126.4, 126.2, 122.7, 118.4, 115.3, 70.8, 70.7, 70.2, 69.7, 69.1, 61.8, 53.7, 52.7.

HRMS (ES$^+$, MeOH): m/z calculated for $C_{32}H_{42}O_8N_8Zn$ $[M+Zn]^{2+}$ 365.1203, found 365.1195.

3.6 Compound Zn((*R*)-6)·2ClO$_4$

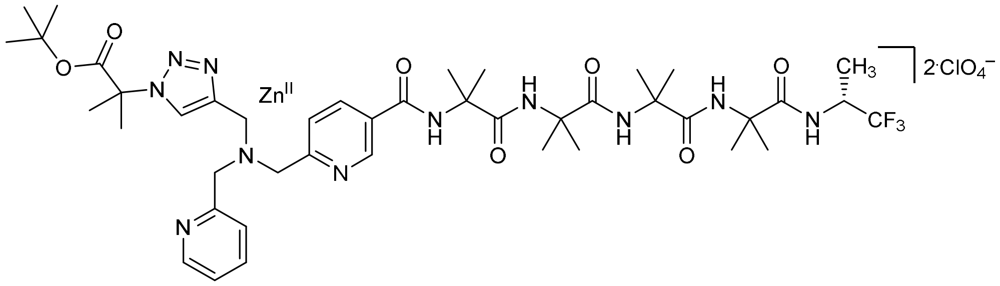

^{1}H NMR (400 MHz, CD_3CN): δ 9.11 (s, 1H, H_{Py}), 8.75 (d, *J* = 5.4 Hz, 1H, H_{Py}), 8.57 (d, *J* = 8.4 Hz, 1H, H_{Py}), 8.18–8.07 (m, 2H, NH + $H_{triazole}$), 7.94 (s, 1H, NH), 7.76–7.66 (m, 2H, 2 × H_{Py}), 7.65–7.52 (m, 4H, 2 × NH + 2 × H_{Py}), 7.26 (s, 1H, NH), 4.62–4.49 (m, 1H, $CHCF_3$), 4.37 (s, 2H, CH_2), 4.30 (s, 2H, CH_2), 4.21 (s, 2H, CH_2), 1.88 (s, 6H, 2 × CH_3), 1.56–1.53 (m, 6H, 2 × CH_3), 1.47–1.45 (m, 3H, CH_3), 1.42 (s, 3H, CH_3), 1.40 (s, 3H, CH_3), 1.36 (m, 12H, 4 × CH_3), 1.34–1.31 (m, 6H, 2 × CH_3), 1.26 (s, 3H, 2 × CH_3).

^{19}F NMR (377 MHz, CD_3CN): δ –77.6 (s, 3F, CF_3).

^{13}C NMR (101 MHz, CD_3CN, as a mixture of diastereoisomers): δ 176.4, 176.3, 175.28, 175.1, 170.3, 164.8, 159.3, 156.3, 149.5, 148.9, 145.0, 143.2, 142.1, 132.2, 128.5, 126.6, 126.3, 126.1, 125.7, 124.7, 84.6, 68.6, 58.6, 58.2, 57.6, 57.6, 57.4, 50.3, 50.0, 47.1 (q, *J* = 31.0 Hz), 27.8, 26.9, 26.3, 25.9, 25.5, 25.4, 24.8, 24.6, 2.2, 13.9.

HRMS (ES$^+$, MeOH): m/z calculated for $C_{42}H_{62}O_{11}N_7F_3Zn$ $[M+Zn]^{2+}$ 482.7026, found 482.7027.

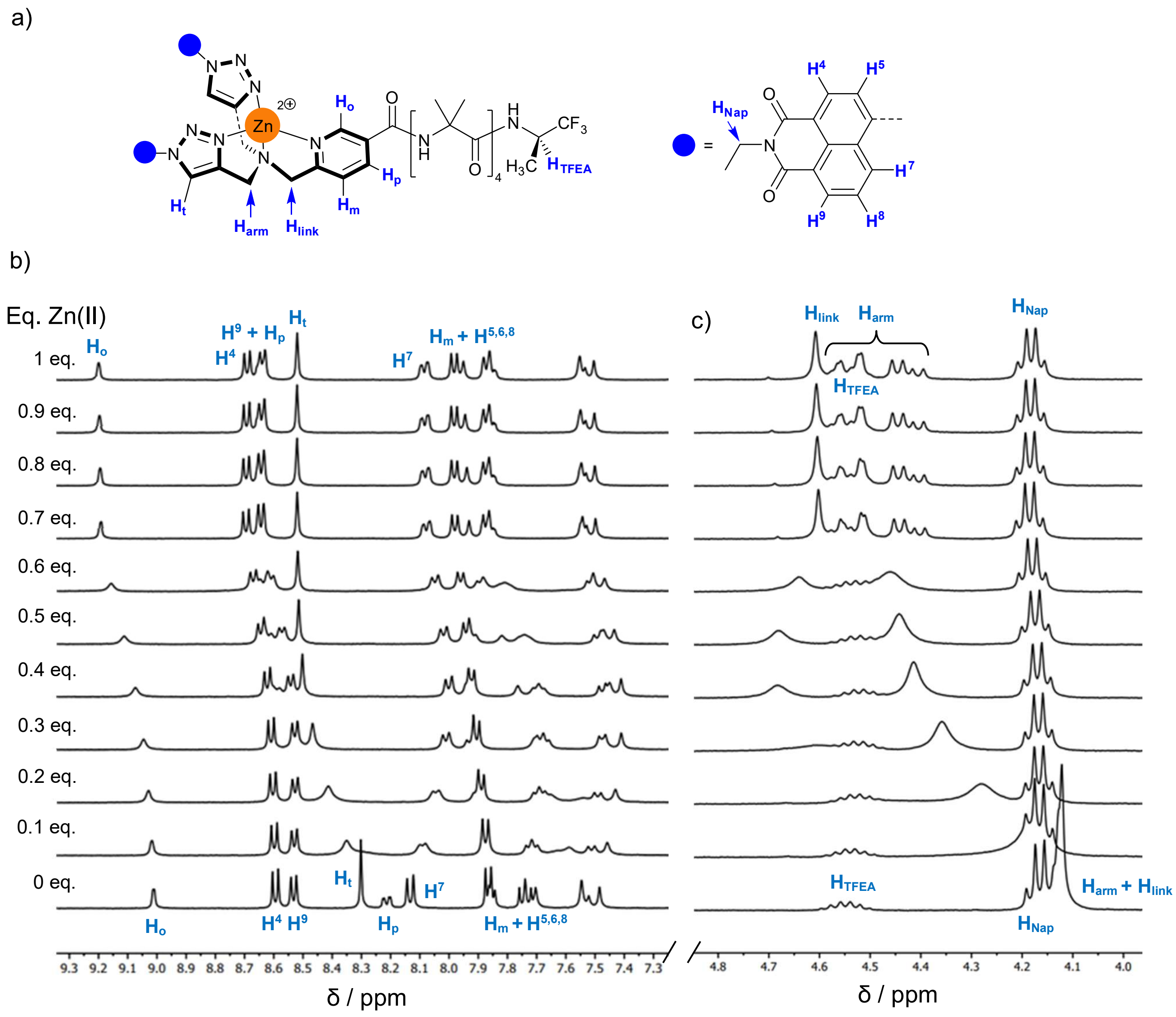

Figure S1 (a) Structure of Zn((*R*)-**2**)·2ClO4 with key protons labelled. (b) Partial ^{1}H NMR spectra (400 MHz, 298 K) showing the (b) aromatic and (c) aliphatic regions for the titration of (*R*)-**2** (4.25 mM, 500 µL) in CD_3CN with 42.5 mM $Zn(ClO_4)_2.6H_2O$ (up to 1 eq.).

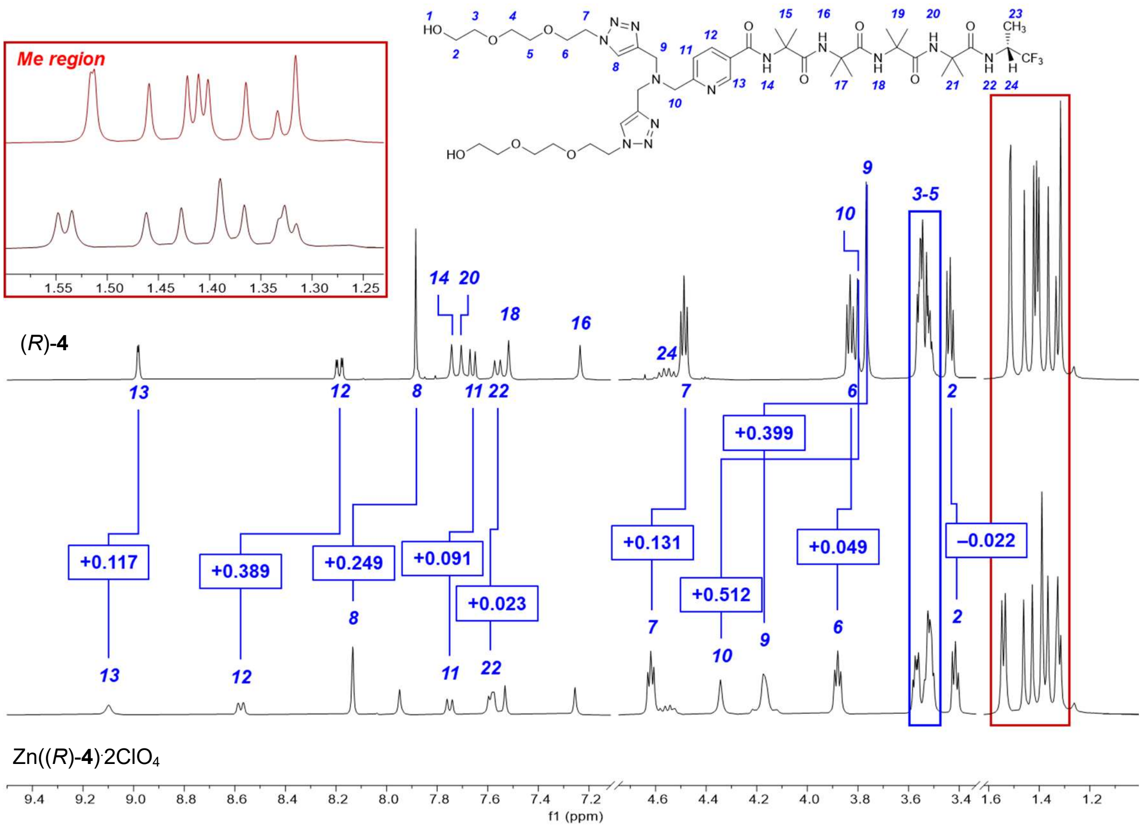

Figure S2 ^{1}H NMR spectra (400 MHz, CD_3CN, 298 K) of compounds (*R*)-**4** (top) and Zn((*R*)-**4**)·2ClO_4 (bottom).

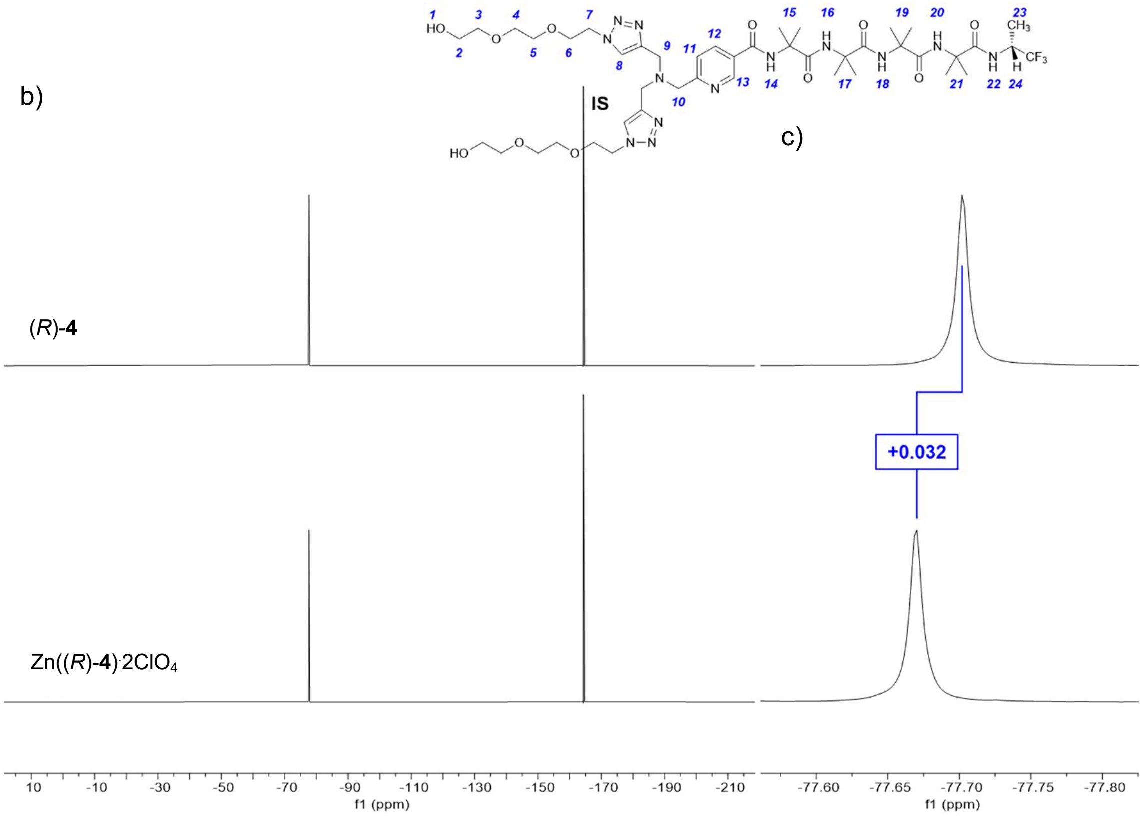

Figure S3 Full width and (c) expanded ^{19}F NMR spectra (377 MHz, CD_3CN, 298 K) of compounds (*R*)-**4** (top) and Zn((*R*)-**4**)·2ClO_4 (bottom). IS: C_6F_6 referenced at –164.38 ppm.

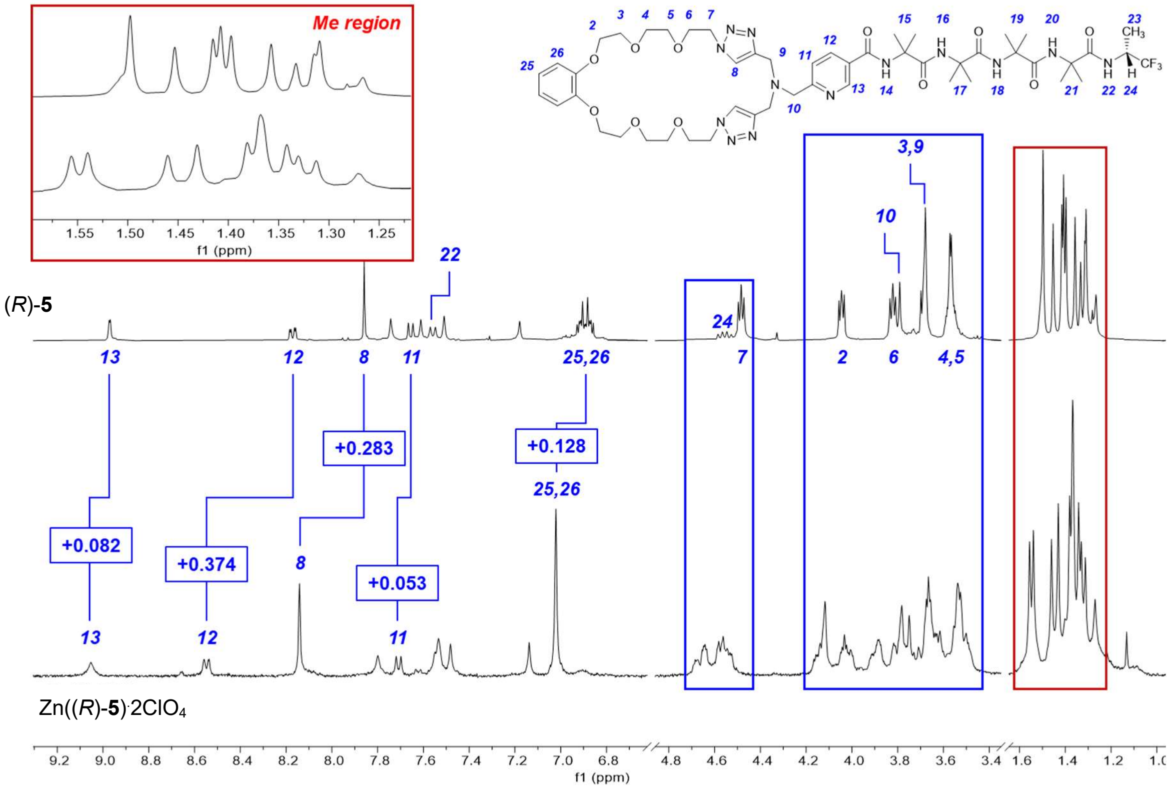

Figure S4 ^{1}H NMR spectra (400 MHz, CD_3CN, 298 K) of compounds (*R*)-**5** (top) and Zn((*R*)-**5**)·$2ClO_4$ (bottom).

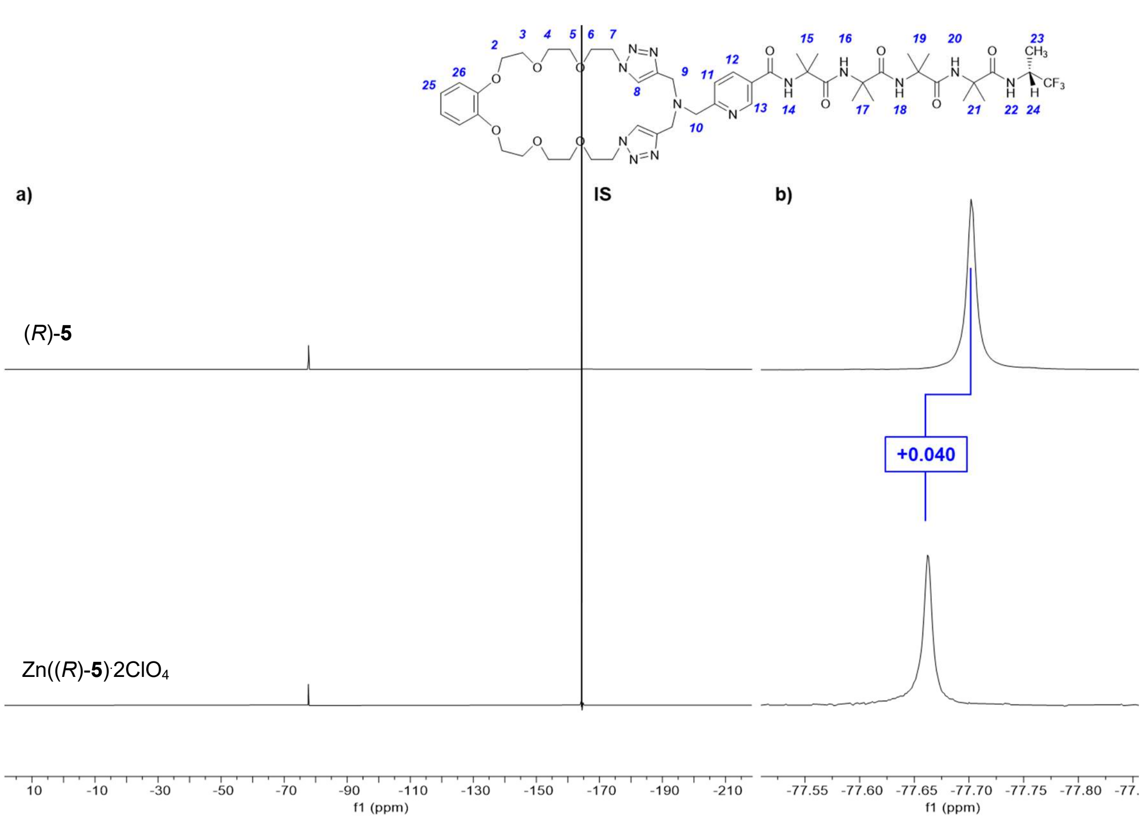

Figure S5 (a) Full sweep width and (b) expanded ^{19}F NMR spectra (377 MHz, CD_3CN, 298 K) of compounds (*R*)-**5** (top) and Zn((*R*)-**5**)·$2ClO_4$ (bottom). IS: C_6F_6 referenced at –164.38 ppm.

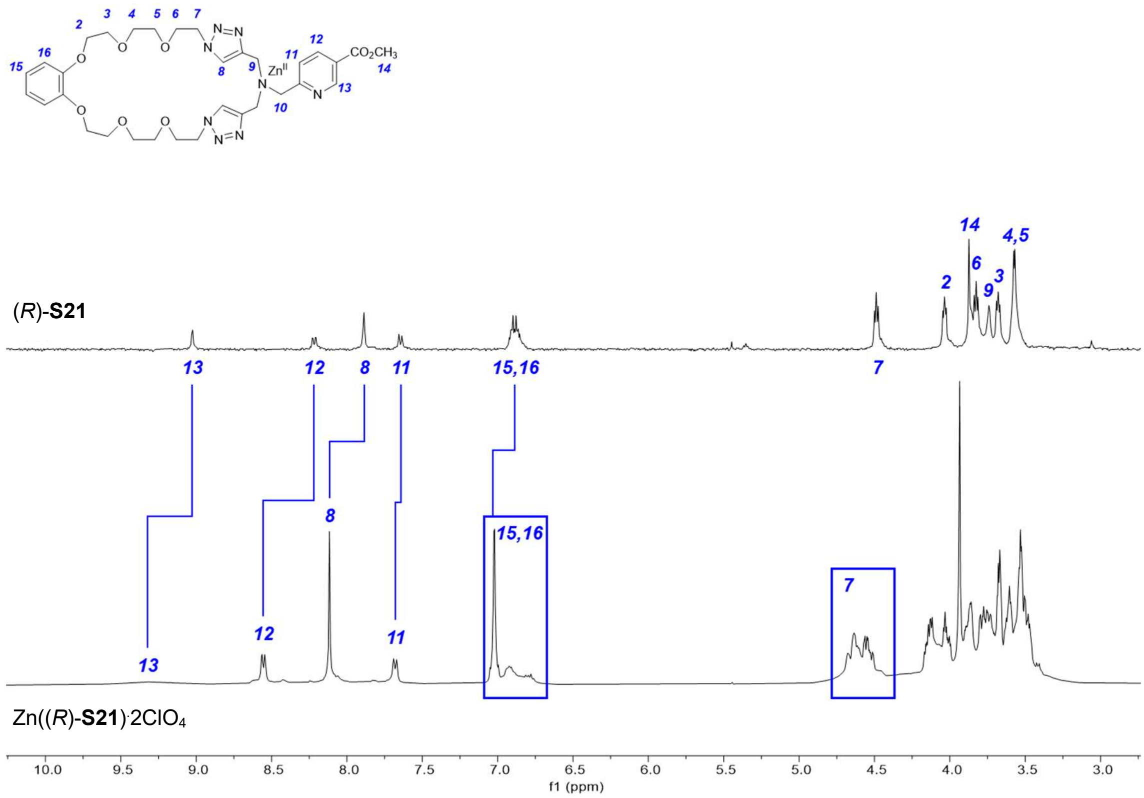

Figure S6 ^{1}H NMR spectra (400 MHz, CD_3CN, 298 K) of compounds (*R*)-**S21** (top) and Zn((*R*)-**S21**)·$2ClO_4$ (bottom).

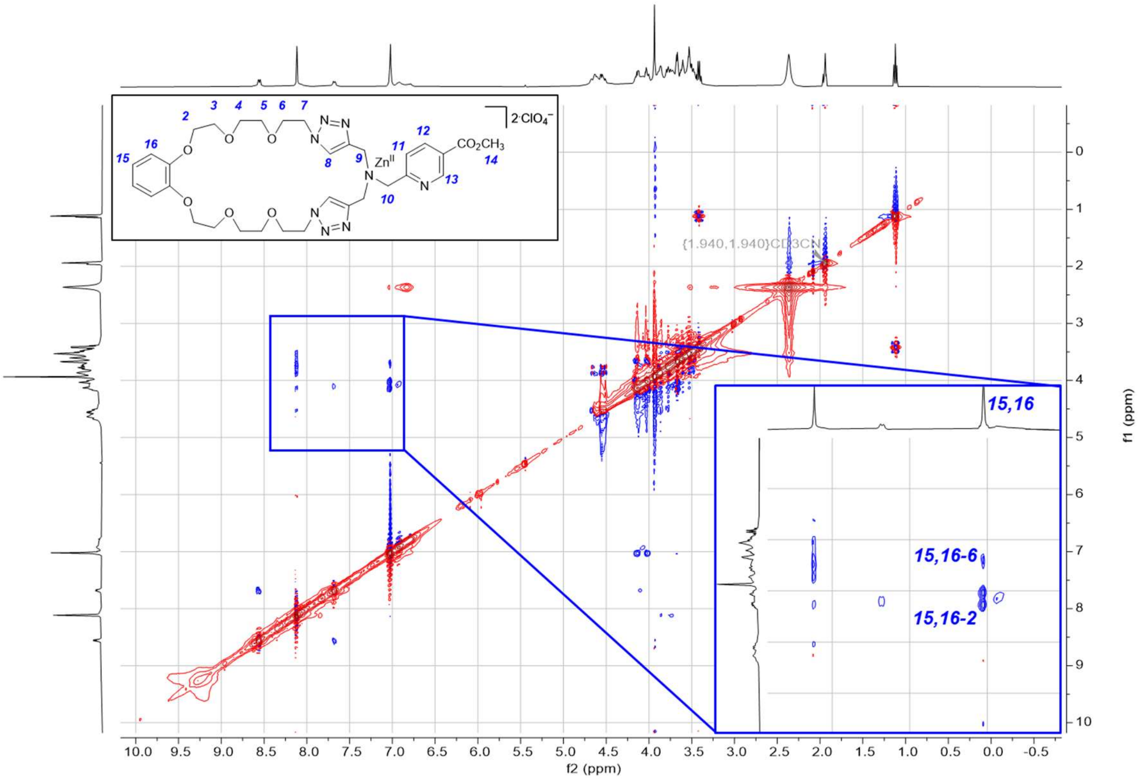

Figure S7 ^{1}H NOESY NMR spectrum (400 MHz, CD_3CN, 298 K) of compound Zn((*R*)-**S21**)·$2ClO_4$.

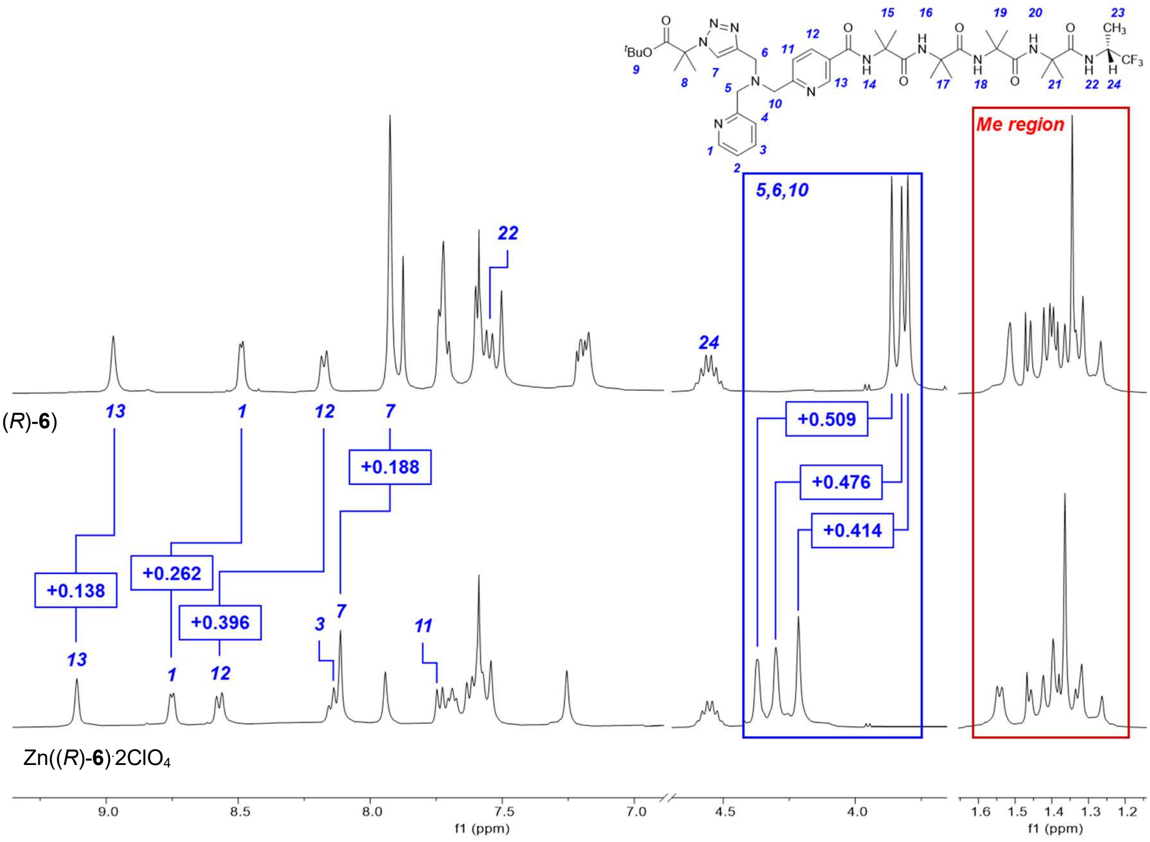

Figure S8 ^1H NMR spectra (400 MHz, CD_3CN, 298 K) of compounds (*R*)-**6** (top) and Zn((*R*)-**6**)·$2ClO_4$ (bottom).

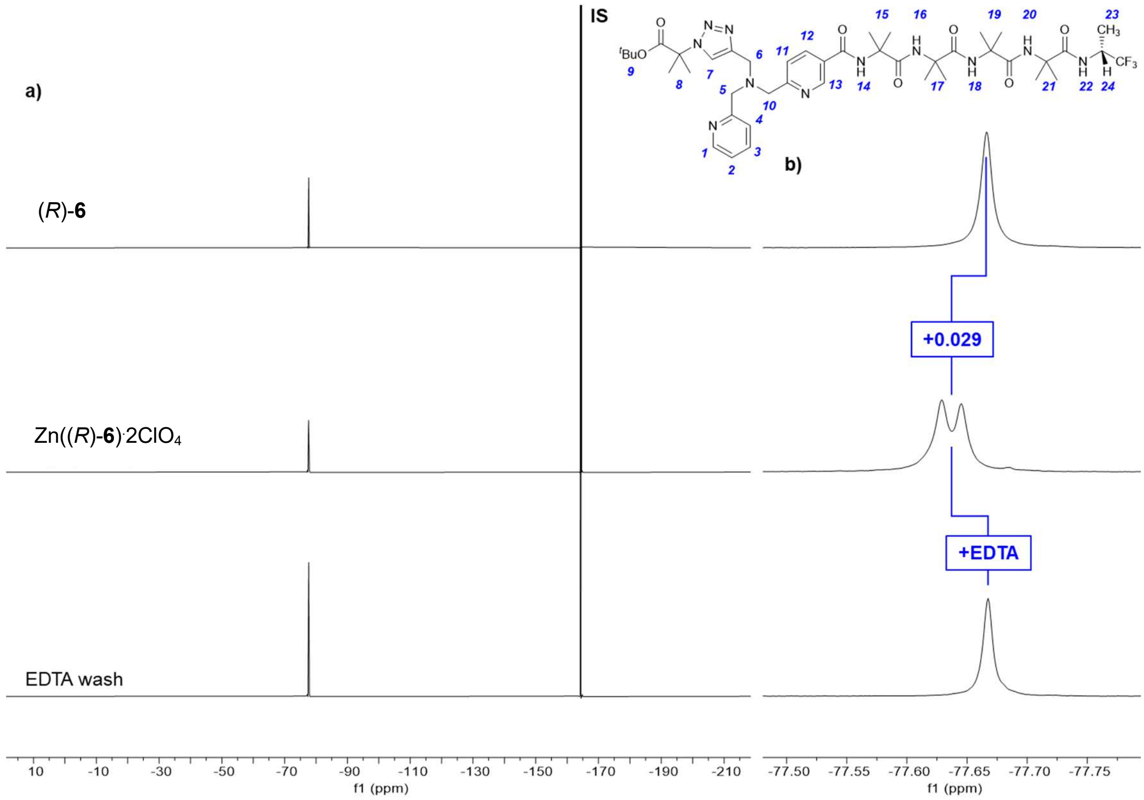

Figure S9 (a) Full sweep width and (b) expanded ^{19}F NMR spectra (377 MHz, CD_3CN, 298 K) of compounds (*R*)-**6** (top) and Zn((*R*)-**6**)·$2ClO_4$ before (middle) and after EDTA wash (bottom). IS: C_6F_6 referenced at –164.38 ppm.

4. Titrations with anions

4.1 General procedures

Procedure 1: Zn((*R*)-**2**)·2ClO$_4$, Zn((*S*)-**2**)·2ClO$_4$, or Zn((*S*)-**3**)·2ClO$_4$ (5.5 µmol or 11 µmol) was dissolved in CD$_3$CN (1.3 mL) to give a 4.25 or 8.5 mM solution. Aliquots of this solution (550 µL) were added to two NMR tubes for the titration with each carboxylate. To each solution, a drop of C$_6$F$_6$ was added as an internal ^{19}F reference. Stock solutions of 47 mM carboxylate/phosphate were made by dissolving each acid (23 µmol) and 2,6-lutidine (3.3 µL, 28 µmol, where appropriate) in deuterated solvent (500 µL). Aliquots of carboxylate/phosphate stock solution (50 µL = 1 eq. carboxylate/phosphate; 1.2 eq. 2,6-lutidine, where appropriate) were sequentially added to the solutions of either Boc-L-Pro or Boc-D-Pro and ^{1}H and ^{19}F NMR spectra were acquired at 0, 0.1, 0.2, 0.3, 0.4, 0.5, 0.6, 0.7, 0.8, 0.9, 1.0, 1.2, 1.4, 1.6, 2.0 eq. of anion.

Procedure 2: Zn((*R*)-**4**)·2ClO$_4$, Zn((*R*)-**5**)·2ClO$_4$, or Zn((*R*)-**6**)·2ClO$_4$ were dissolved in CD$_3$CN and further diluted to reach a concentration of 2 mM in 600 µL (including 10 µL of C$_6$F$_6$ as internal standard). The solutions were transferred into a NMR tube and ^{1}H and ^{19}F spectra were recorded for reference. Stock solutions of carboxylic acids (Boc-D-Pro, Boc-L-Pro, 24.2 mM) were prepared with 2,6-lutidine (29.0 mM) and Zn((*R*)-**4**-**6**)·2ClO$_4$ (2 mM). Tetrabutylammonium acetate (24.2 mM) was used without 2,6-lutidine but with Zn((*R*)-**4**-**6**)·2ClO$_4$ (2 mM). Aliquots of the appropriate anion solution were added sequentially (5 µL, or 10 µL, or 25 µL) and NMR spectra were recorded after every addition.

4.2 Fitting of titration data

The change in chemical shift of the some peaks of Zn(**2**)·2ClO$_4$ were fitted to a 2:1 host/guest binding isotherm using the software DynaFit.[S7] The change in chemical shift of the some peaks of Zn(**4-6**)·2ClO$_4$ (see Sections 4.5, 4.6 and 4.7 for details) were fitted to a 2:1 host/guest binding isotherm using either the software SupraFit.[S8]

Given the sigmoidal profile observed from the titration experiments (Figures 7 and 8; ESI, Sections 4.3, 4.5), we performed our data fitting by employing a 2:1 host/guest binding model which describes two binding events according to Equations 1,2. H = foldamer and G = ligand

$$H + G \rightleftharpoons HG \qquad K_{11} = \frac{[HG]}{[H][G]} \qquad (1)$$

$$H + HG \rightleftharpoons H_2G \quad K_{21} = \frac{[H_2G]}{[H][HG]} \qquad (2)$$

For Zn((*R*)-**4**)·2ClO$_4$, a global fitting was performed with multiple ^{1}H peaks with a high degree of confidence. On the contrary, we could only fit the triazole proton (H$_a$) for titrations with Zn((*R*)-**5**)·2ClO$_4$ as some peaks were split or overlapped with other species (e.g. 2,6-lutidine). We also performed data fitting of the ^{19}F experimental data for Zn((*R*)-**2**)·2ClO$_4$ and Zn((*R*)-**4-6**)·2ClO$_4$,

All fittings confirm that titrations are reproducible within the experimental conditions by using different batches of Zn-complexed foldamers.

4.3 Host: Zn((*R*)-2)·2ClO$_4$

4.3.1 Titration with tetrabutylammonium acetate (^{1}H NMR)

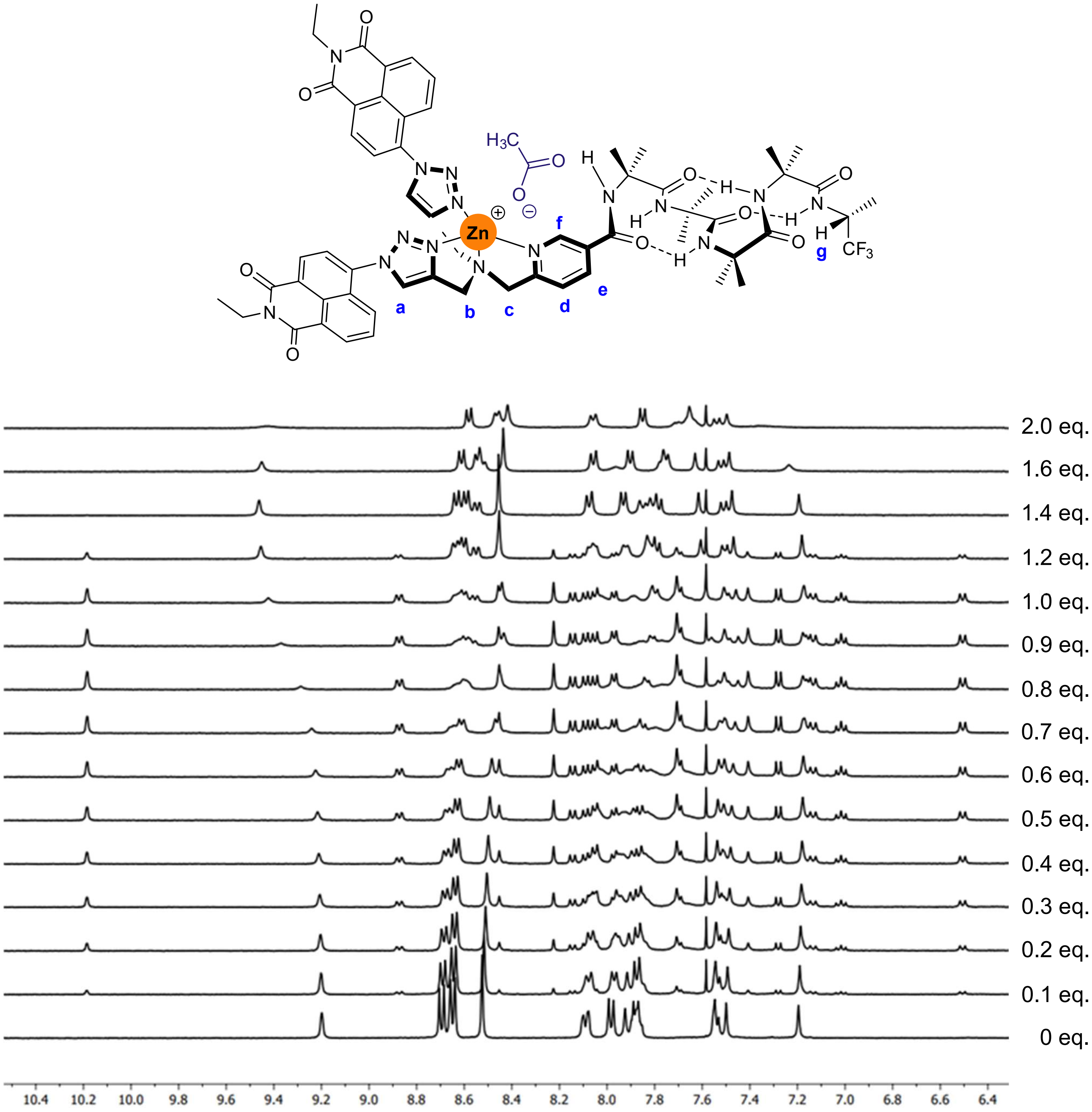

Figure S10 Partial ^{1}H NMR spectra (400 MHz, 298 K) showing the binding site region for the titration of Zn((*R*)-**2**)·2ClO$_4$ (4.25 mM, 550 µL) in CD_3CN with TBA acetate (47 mM, up to 2 eq.).

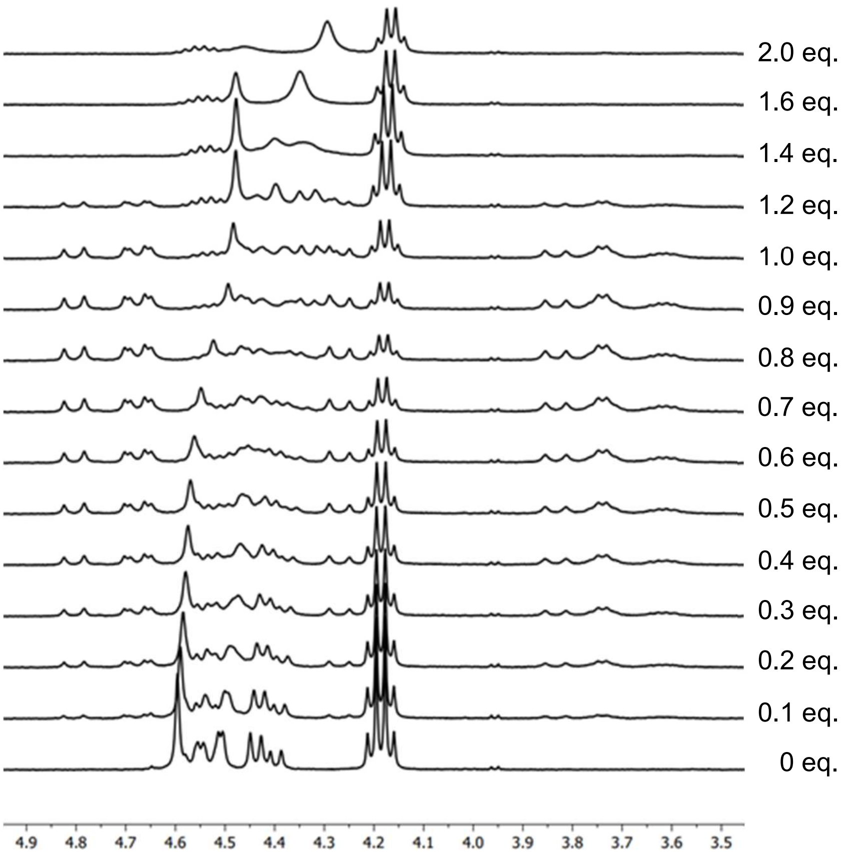

Figure S11 Partial ^1H NMR spectra (400 MHz, 298 K) showing the binding site region for the titration of Zn((*R*)-**2**)·2ClO$_4$ (4.25 mM, 550 μL) in CD_3CN with TBA acetate (47 mM, up to 2 eq.).

4.3.2 Titration with tetrabutylammonium acetate (^{1}H NMR)

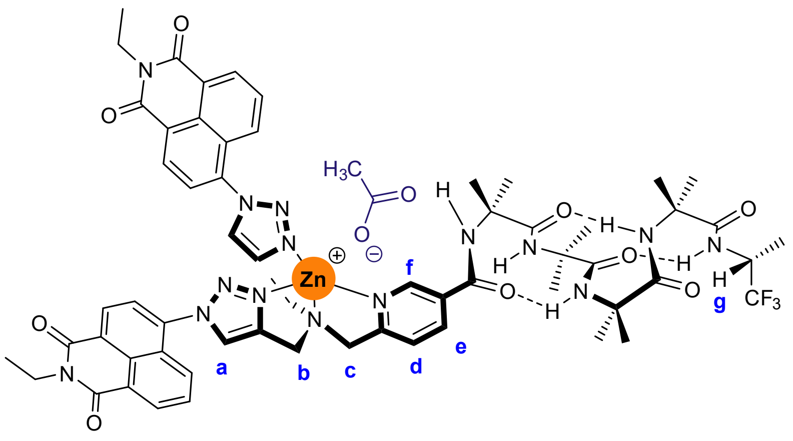

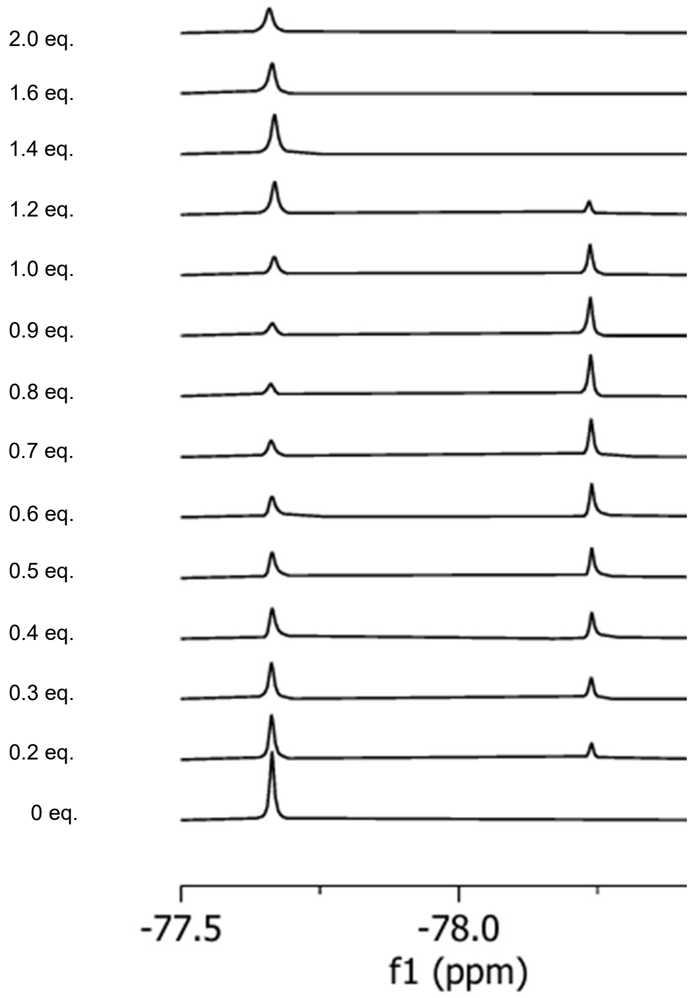

Figure S12 Partial ^{19}F NMR spectra (376 MHz, 298 K) showing the TFEA reporter region for the titration of Zn((*R*)-**2**)·2ClO$_4$ (4.25 mM, 550 μL) in CD_3CN with TBA acetate (47 mM, up to 2 eq.).

4.3.3 Diffusion ordered spectroscopy (DOSY) with TBA acetate

^{1}H DOSY spectra of uncomplexed Zn((*R*)-**2**)·2ClO$_4$ and upon addition of 0.7 eq. of TBA acetate were acquired (**Figure S13**) revealed new peaks that belong to a single separate species.

a)

b)

c)

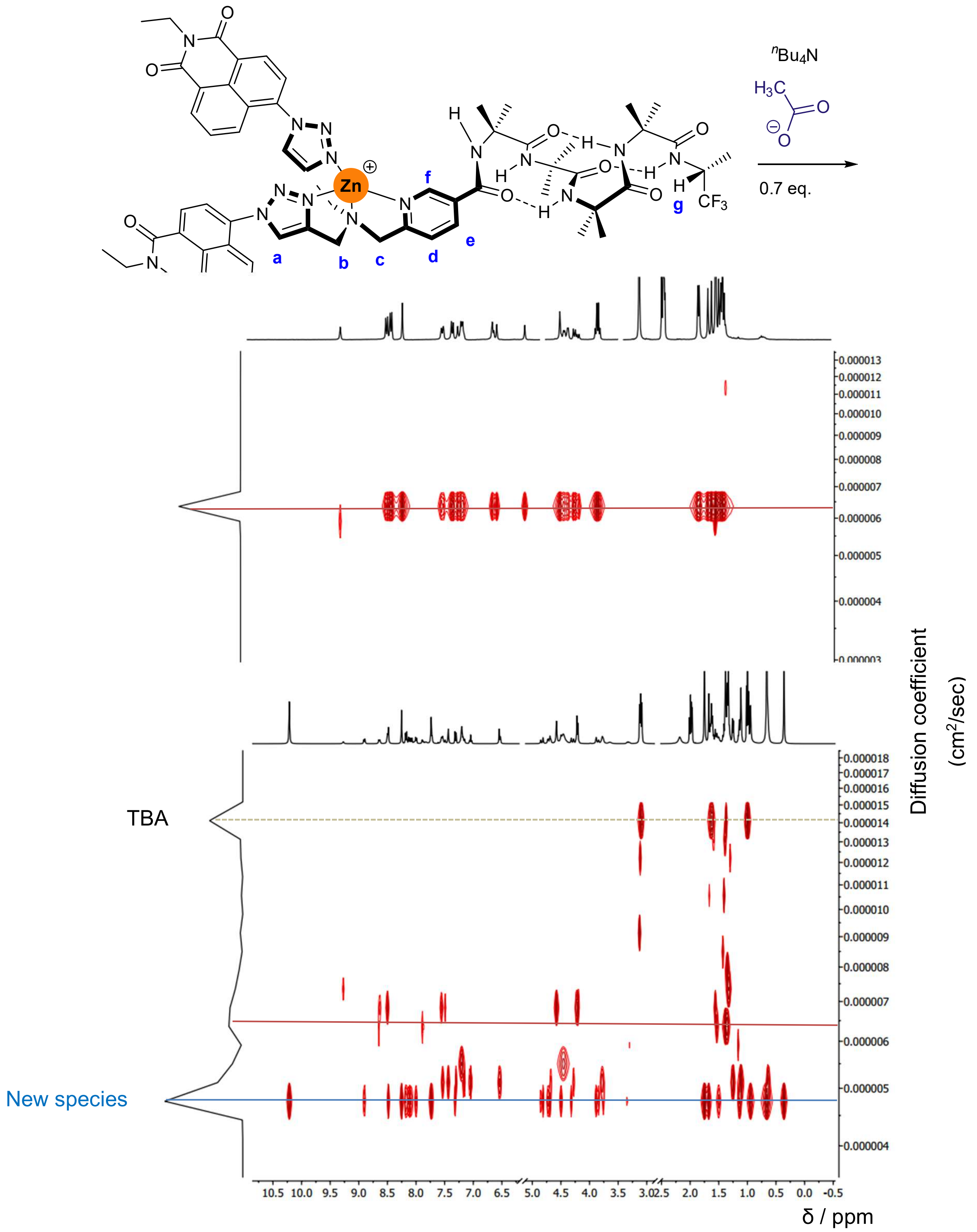

Figure S13 Partial ^{1}H DOSY NMR spectra (400 MHz, 298 K) of uncomplexed Zn((*R*)-**2**)·2ClO$_4$ (4.25 mM, 550 µL), above, and upon the addition of 0.7 eq. TBA acetate, below, in CD$_3$CN.

The Stokes-Einstein equation describes the diffusion of a molecule in solvent, defining the diffusion coefficient (D) as:

$$D = \frac{k_b T}{6\pi\eta r_H}$$

Where k_b is the Boltzmann constant, T is the temperature, η is the solvent viscosity and r_H is the hydrodynamic radius of the molecule.[S9] From **Figure S13**, diffusion coefficients of 6.4×10^{-10} m^2/s and 4.8×10^{-10} m^2/s for uncomplexed Zn((*R*)-**2**)·$2ClO_4$ and the 'new species', respectively, were obtained, correlating to hydrodynamic radii of 10.2 Å and 13.6 Å, respectively. The DOSY therefore indicates that these new peaks belong to a larger species. It is possible that the formation of this species is due to the relatively small ligand size of acetate, leaving the binding site more available to complexation with another foldamer compared to the Boc-protected carboxylic acids. Upon addition of 0.7 eq. TBA acetate, the solution contains a mixture of the larger species and another with a similar diffusion coefficient of the uncomplexed foldamer. As the Bayesian DOSY transform is a quantitative method, this allows the relative composition of the mixture to be ascertained by integration of the diffusion trace.[S10] This gave a ratio of 1:3 of the small:large species.

4.3.4 Titration with Boc-Pro/2,6-lutidine (^{1}H NMR)

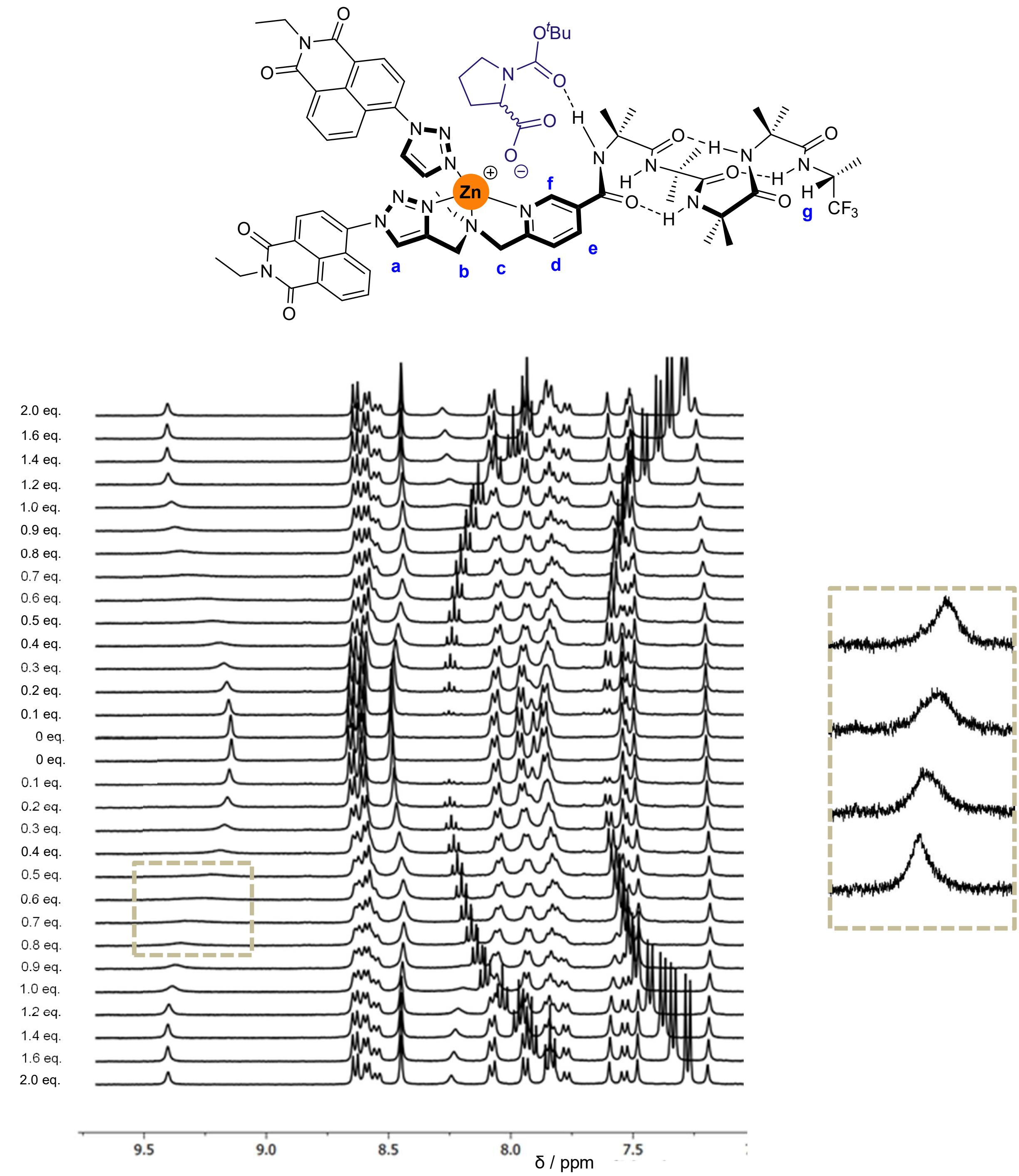

Figure S14 Partial ^{1}H NMR spectra (400 MHz, 298 K) showing the aromatic binding site region for the titration of Zn((*R*)-**2**)·2ClO$_4$ (4.25 mM, 550 µL) in CD$_3$CN with 47 mM Boc-Pro (up to 2 eq. and 2.4 eq. 2,6-lutidine). Brown box expands the *ortho*-pyridyl resonance (position f).

4.3.5 Titration with Boc-Pro/2,6-lutidine (^{19}F NMR)

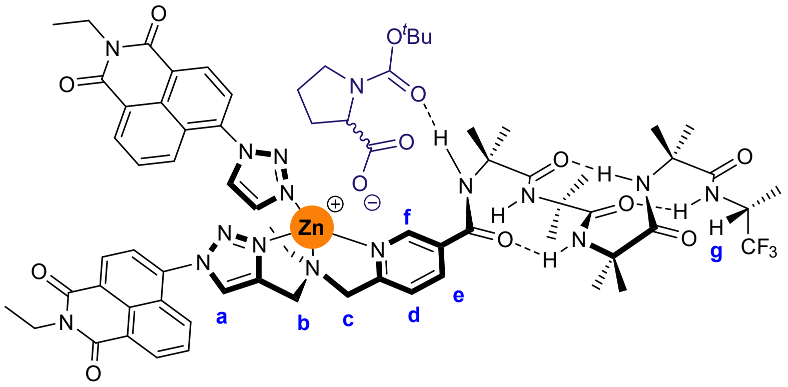

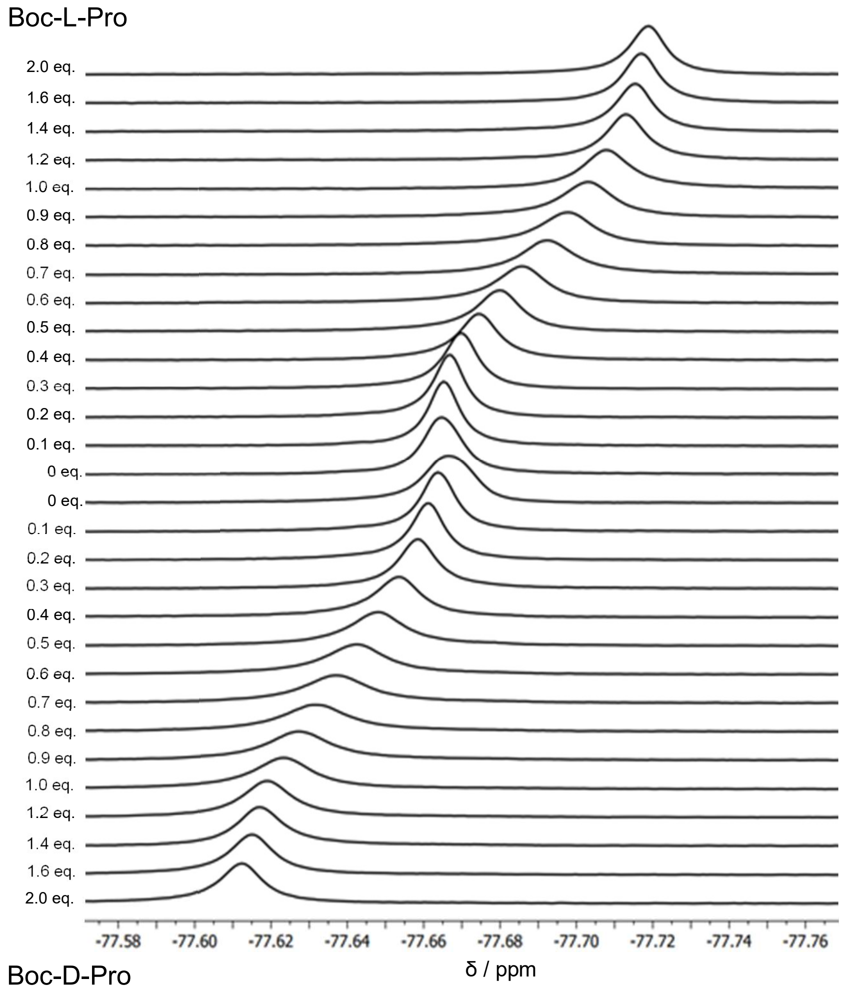

Figure S15 Partial ^{19}F NMR spectra (376 MHz, 298 K) showing TFEA region for the titration of Zn((*R*)-**2**)·2ClO$_4$ (4.25 mM, 550 μL) in CD$_3$CN with 47 mM Boc-Pro (up to 2 eq. and 2.4 eq. 2,6-lutidine). C$_6$F$_6$ as an internal standard, referenced at -164.38 ppm.[S2] Induced screw-sense direction can be determined from the direction of chemical shift movement in the ^{19}F NMR spectra by comparison to covalently controlled (*R*)-TFEA terminated foldamers reported by Wang *et al.*[S5]

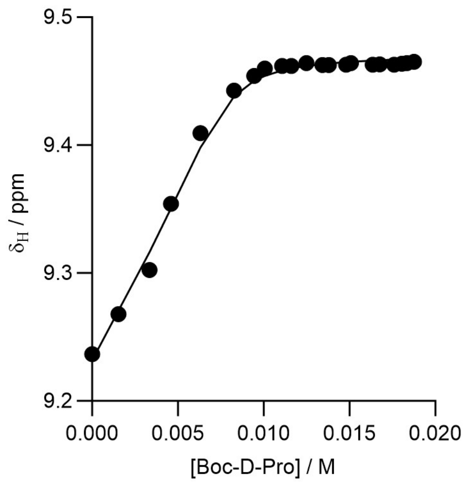

Figure S16 Representative data fitting of $\Delta\delta_H$(ortho-CH/H_f) using Dynafit during the titration of Zn((*S*)-**2**)·2ClO$_4$ in CD$_3$CN with Boc-D-Pro. Binding model: 2:1 [Zn((*S*)-**2**)·2ClO$_4$]/[Boc-D-Pro] with K_{11} = 1 × 10^5 M^{-1} and K_{21} = 2 × 10^3 M^{-1}. Conditions: [Zn((*S*)-**2**)·2ClO$_4$] = 8.36 mM, [Boc-Pro] = 0–18.9 mM, [2,6-lutidine] = 0–22.7 mM. CH$_3$CN as an internal standard, referenced at 1.96 ppm.[S11]

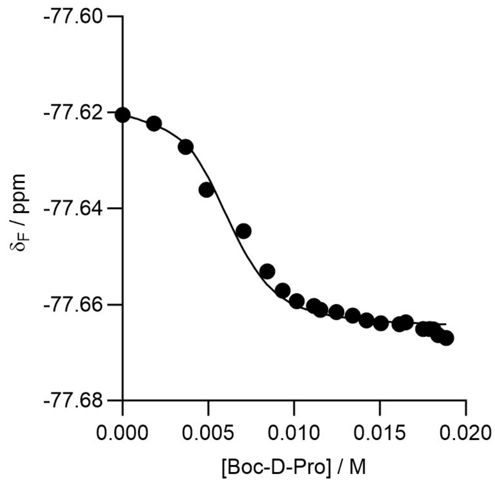

Figure S17 Representative data fitting of $\Delta\delta_F$(CF$_3$) using Dynafit during the titration of Zn((*S*)-**2**)·2ClO$_4$ in CD$_3$CN with Boc-D-Pro. Binding model: 2:1 [Zn((*S*)-**2**)·2ClO$_4$]/[Boc-D-Pro] with K_{11} = 1 × 10^5 M^{-1} and K_{21} = 2 × 10^3 M^{-1}. Conditions: [Zn((*S*)-**2**)·2ClO$_4$] = 8.36 mM, [Boc-Pro] = 0–18.9 mM, [2,6-lutidine] = 0–22.7 mM. CFCl$_3$ as an internal standard, referenced at −1.14 ppm.[S2,S12]

4.3.6 Titration with Boc-Pip/2,6-lutidine (^{1}H NMR)

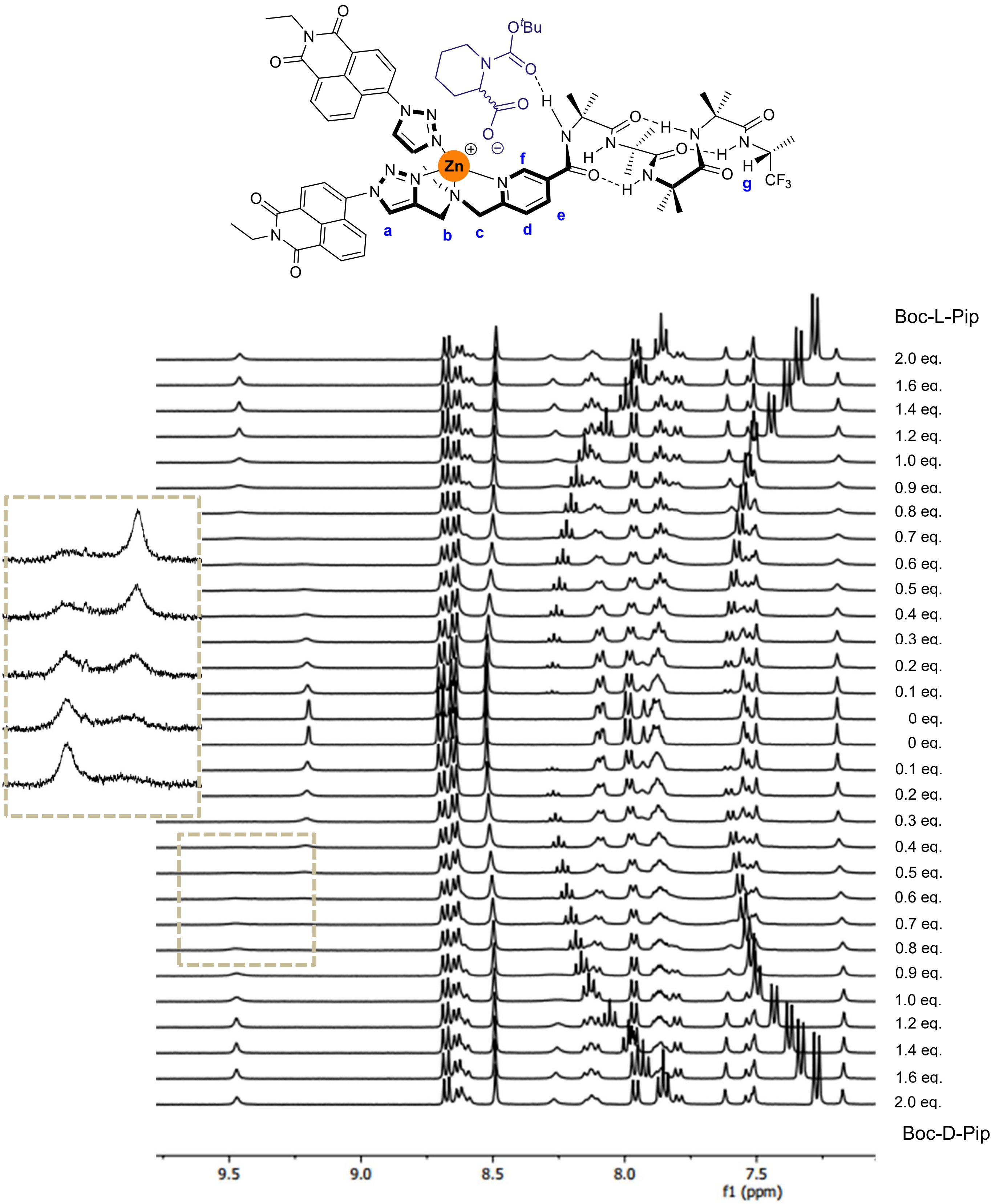

Figure S18 Partial ^{1}H NMR spectra (400 MHz, 298 K) showing the aromatic binding site region for the titration of Zn((*R*)-**2**)·2ClO$_4$ (4.25 mM, 550 µL) in CD$_3$CN with 47 mM Boc-Pip (up to 2 eq. and 2.4 eq. 2,6-lutidine). Brown box expands the *ortho*-pyridyl resonance (position **f**).

4.3.7 Titration with Boc-Pip/2,6-lutidine (^{19}F NMR)

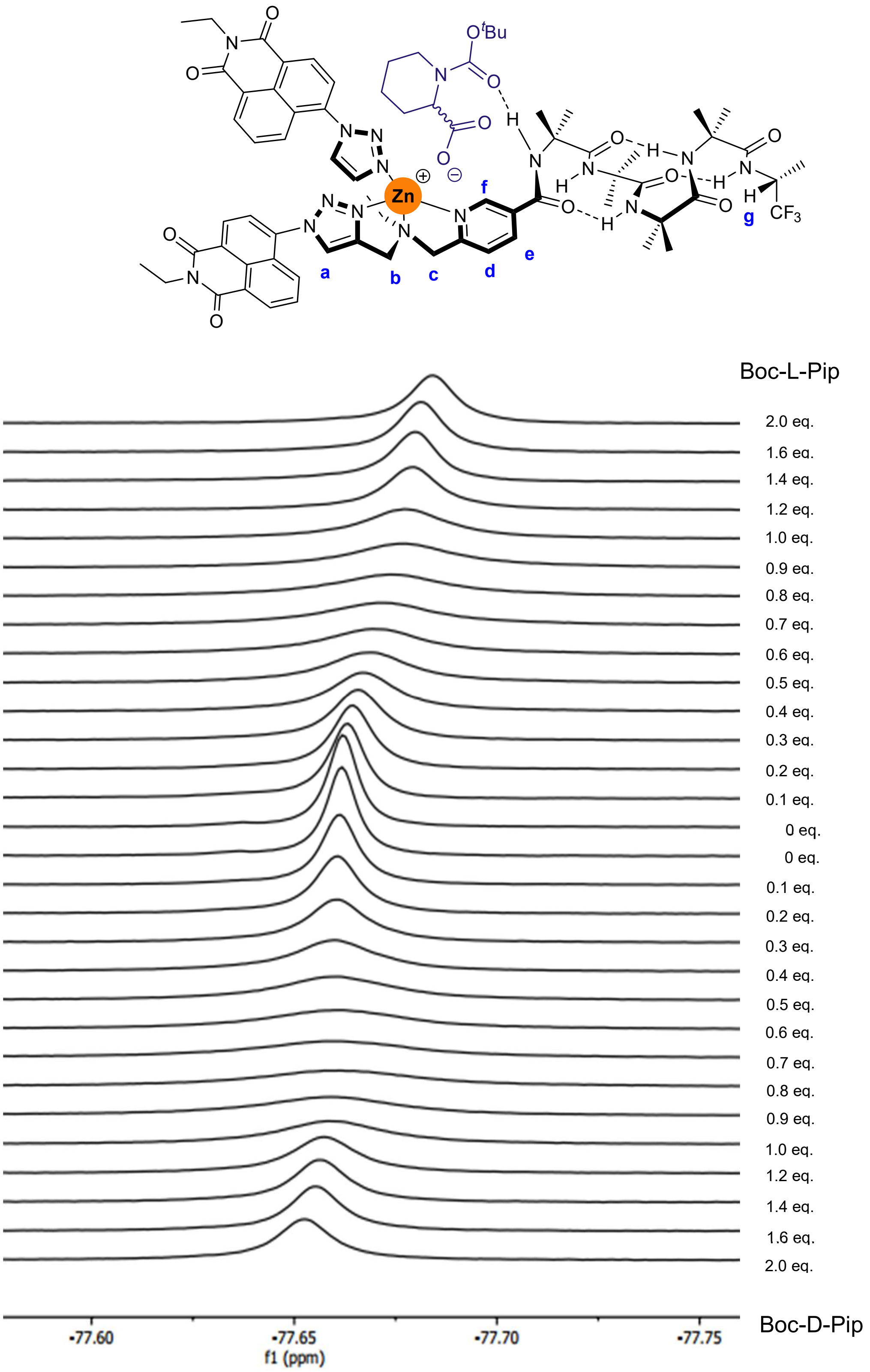

Figure S19 Partial ^{19}F NMR spectra (376 MHz, 298 K) showing TFEA region of Zn((*R*)-**2**)·2ClO$_4$ in CD$_3$CN (4.25 mM, 550 µL) titrated with 47 mM Boc-Pip (up to 2 eq. and 2.4 eq. 2,6-lutidine) with C$_6$F$_6$ as an internal standard, referenced at -164.38 ppm.[S2] Induced screw-sense direction can be determined from the direction of chemical shift movement in the ^{19}F NMR spectra by comparison to covalently controlled (*R*)-TFEA terminated foldamers reported by Wang *et al.*[S5]

4.3.8 Titration with TiPSY/2,6-lutidine (^{1}H NMR)

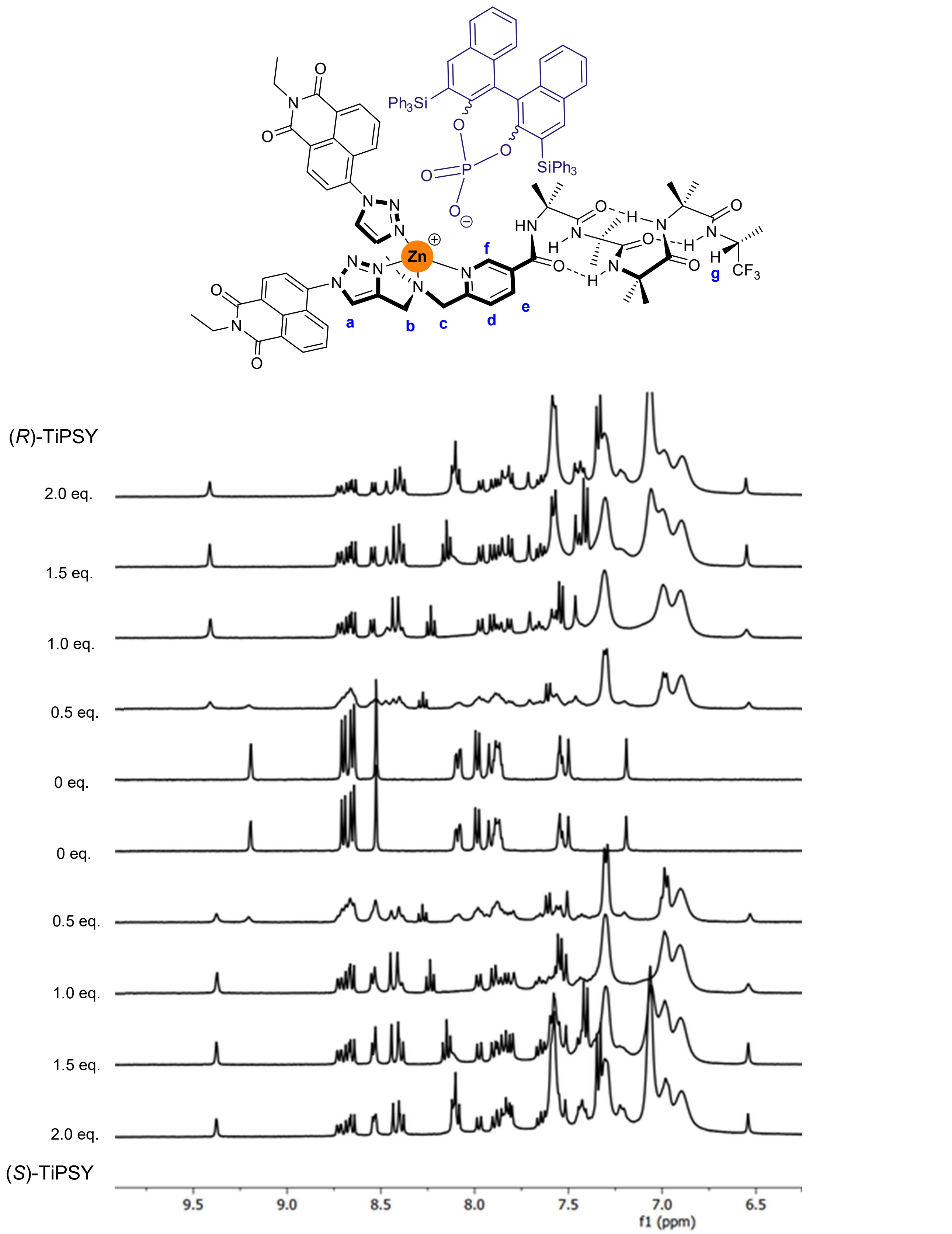

Figure S20 Partial ^{1}H NMR spectra (400 MHz, 298 K) showing the aromatic binding site region for the titration of Zn((*R*)-**2**)·2ClO$_4$ (4.25 mM, 550 µL) in CD$_3$CN with 47 mM TiPSY (up to 2 eq. and 2.4 eq. 2,6-lutidine).

Binding of TiPSY to the BTPA binding site also gave two distinct resonance **a** signals (**Figure S20**), the upfield signal decreasing in magnitude as the downfield peak increases in strength, the latter showing an overall downfield shift of 198 ± 21 ppb. Interestingly, binding of TiPSY did not induce as large a change in resonance position of resonance **a** as the carboxylic acids, possibly indicative of the binding site adopting a different conformation compared to these ligands or some shielding by the aromatic groups of the TiPSY ligand. Another distinction observed upon TiPSY binding compared to the carboxylates is the more apparent difference in induced downfield chemical shift of H**a** between ligand enantiomers. At Zn((*R*)-**2**)·$2ClO_4$ (**Figure S20**), addition of (*S*)-TiPSY results in a greater shift compared to (*R*)-TiPSY, whereas with the enantiomeric receptor (*S*)-**2** the opposite is observed, with addition of (*R*)-TiPSY resulting in a greater shift compared to (*S*)-TiPSY. The equal but opposite responses observed between receptor enantiomers may highlight the influence of C-terminal reporter chirality, and not for example an error in titrant additions. Furthermore, upon binding of TiPSY at the BTPA binding site of (*R*)-**2**, in addition to the downfield shift of the *ortho*-pyridyl proton, the binding site signals appear to be split. Although many are overlapped by the ligand binaphthyl protons, we can observe the splitting of naphthalimide doublets (Δδ = 136 ppb for resonance **i**, 260 ppb for resonance **e**) and triazole singlets (resonance **d**, Δδ = 24 ppb) of the now distinct arms (**Figure S21**). This distinction between the binding site arms upon ligand binding could be attributed to tight binding of the chiral TiPSY ligand.

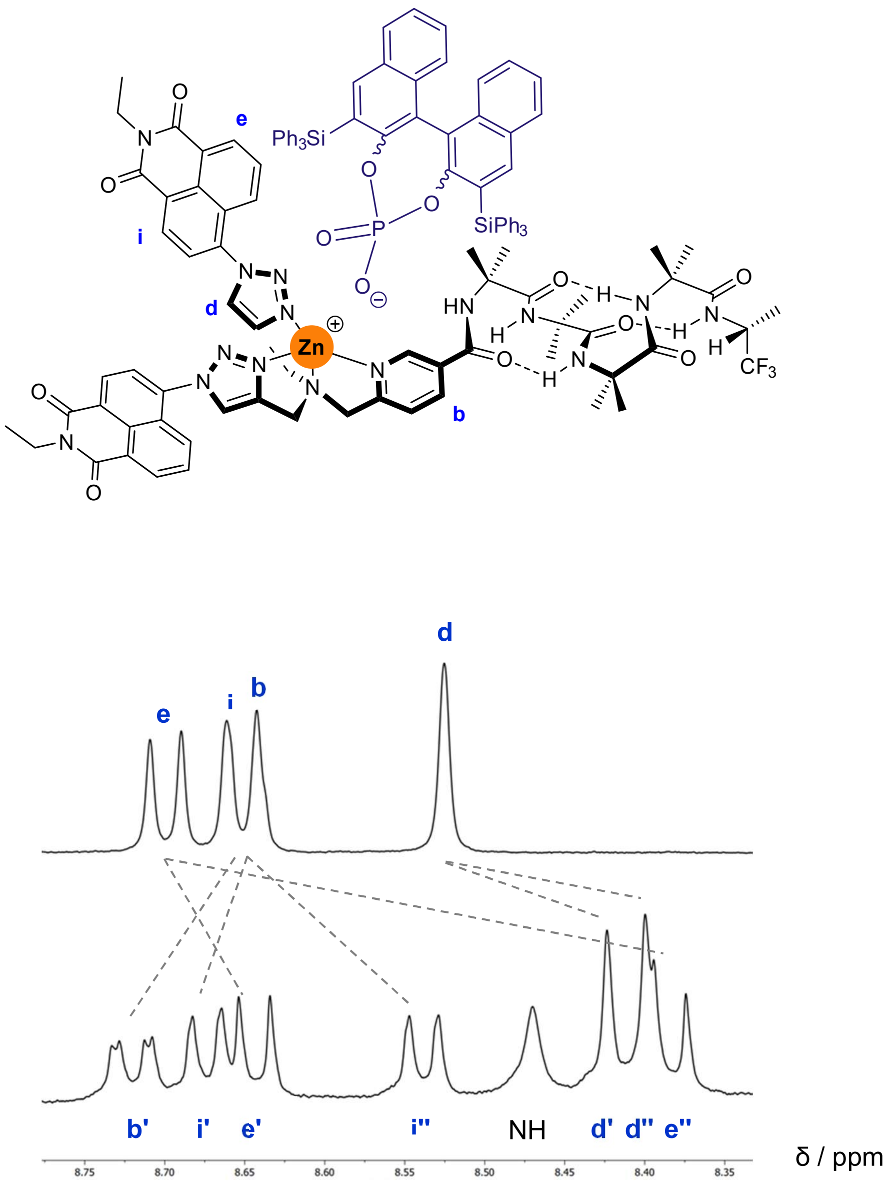

Figure S21 Partial ^{1}H NMR spectra showing a region of the BTPA binding site for the titration of Zn((*R*)-**2**)·2ClO$_4$ (4.25 mM) with 2 eq. of (*S*)-TiPSY in CD$_3$CN. Resonance assignment are made before the addition of (*S*)-TiPSY (top) and after the addition 2 eq. of (*S*)-TiPSY (bottom).

4.3.9 Titration with TiPSY/2,6-lutidine (^{19}F NMR)

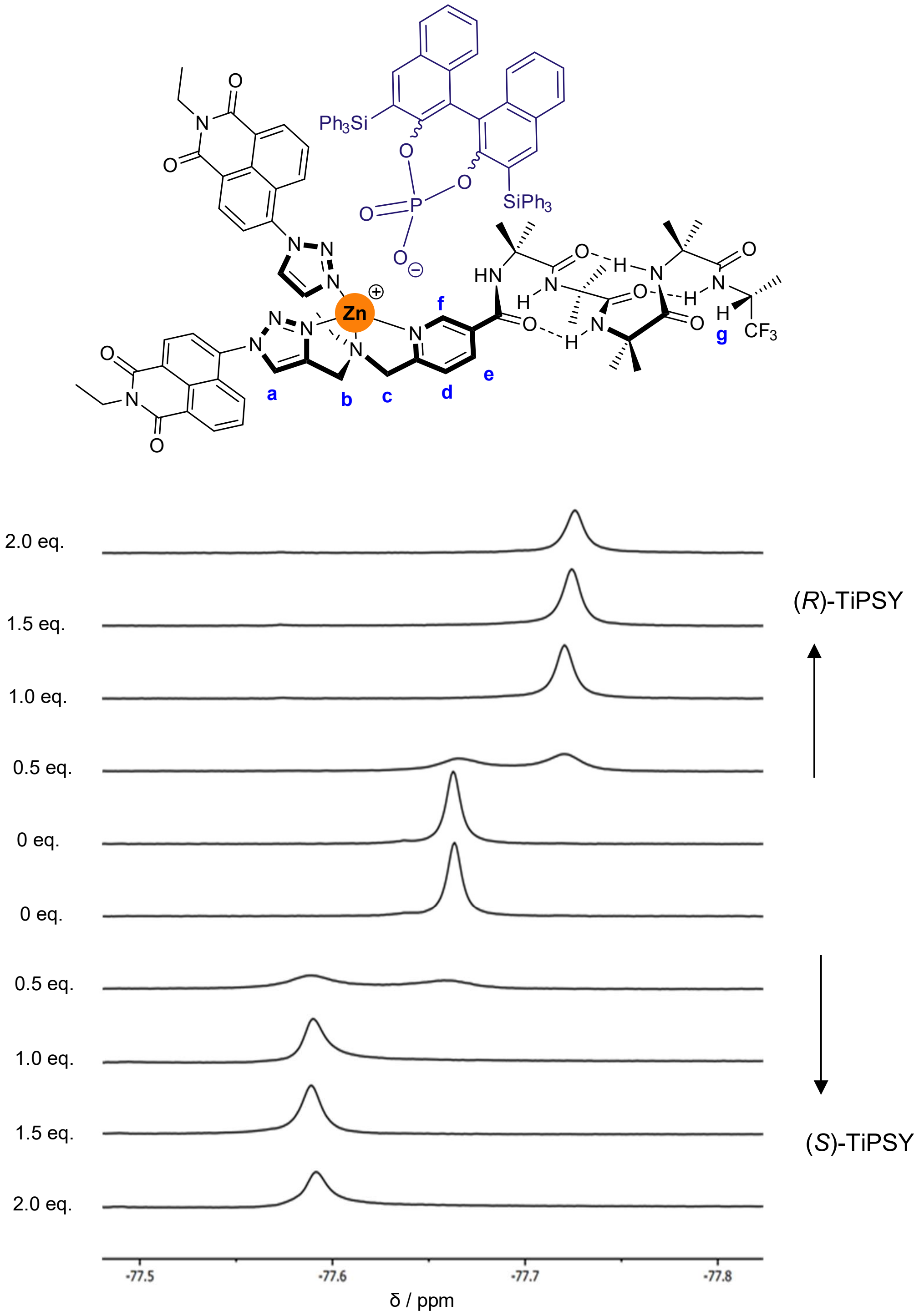

Figure S22 Partial ^{19}F NMR spectra (376 MHz, 298 K) showing TFEA region of Zn((*R*)-**2**)·2ClO$_4$ in CD$_3$CN (4.25 mM, 550 µL) titrated with 47 mM TiPSY (up to 2 eq. and 2.4 eq. 2,6-lutidine) with C$_6$F$_6$ as an internal standard, referenced at -164.38 ppm.[S2] Induced screw-sense direction can be determined from the direction of chemical shift movement in the ^{19}F NMR spectra by comparison to covalently controlled (*R*)-TFEA terminated foldamers reported by Wang *et al.*[S5]

4.4 Host: $Zn((S)\text{-}3)\cdot 2ClO_4$

4.4.1 Titration with Boc-Pro/2,6-lutidine (^1H NMR)

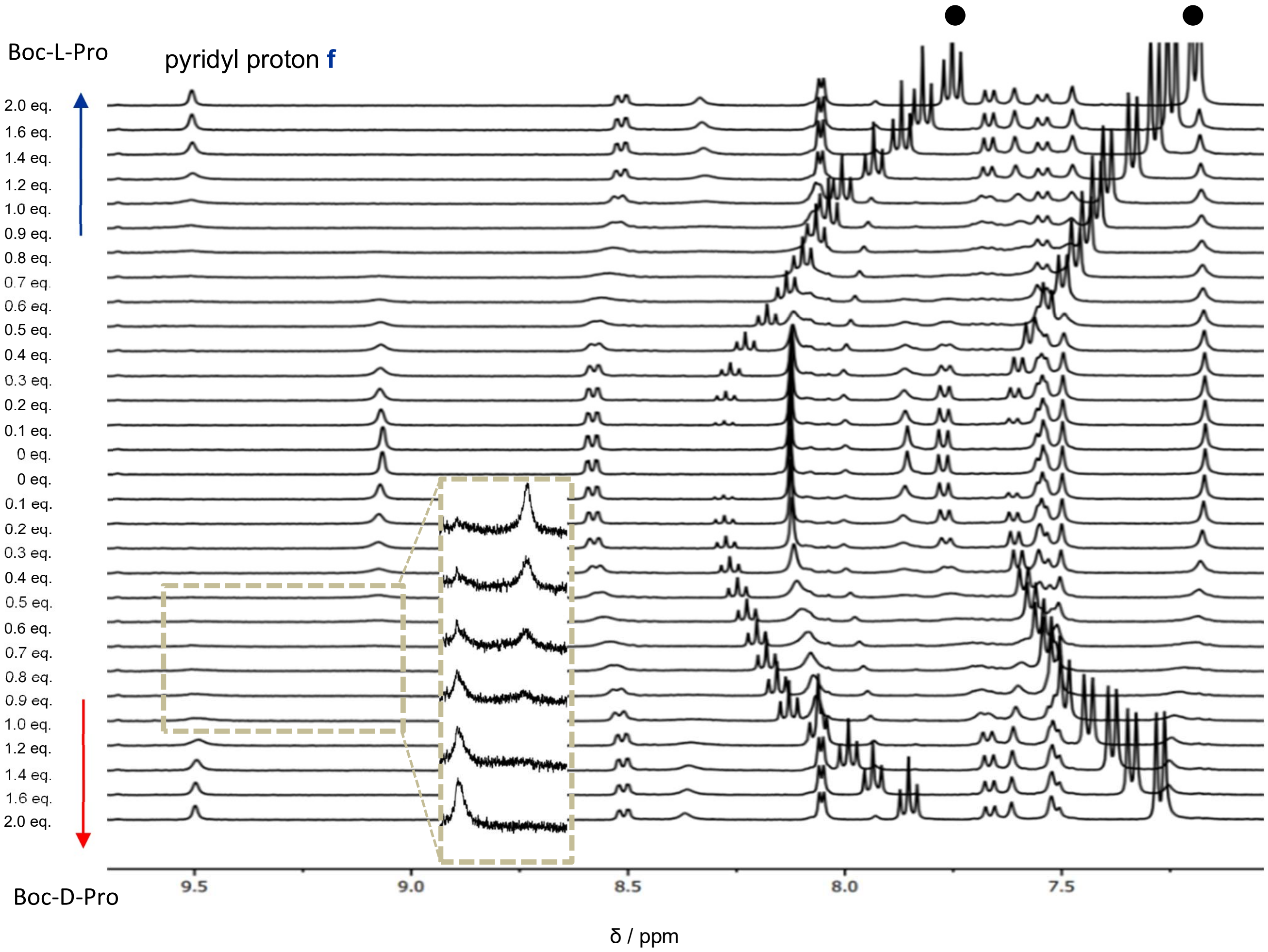

Figure S23 Partial stacked ^1H NMR spectra (CD_3CN, 400 MHz, 298 K) of the titration of $Zn((S)\text{-}\mathbf{3})\cdot 2ClO_4$ with Boc-Pro. Conditions: $[Zn((S)\text{-}\mathbf{3})\cdot 2ClO_4]$ = 4.25 mM, [Boc-D-Pro] or [Boc-L-Pro] = 47 mM, 0–2 equiv, [2,6-lutidine] = 0–2.4 equiv. Brown box expands the *ortho*-pyridyl resonance (position **f**).

4.4.2 Titration with Boc-Pro/2,6-lutidine (^{19}F NMR)

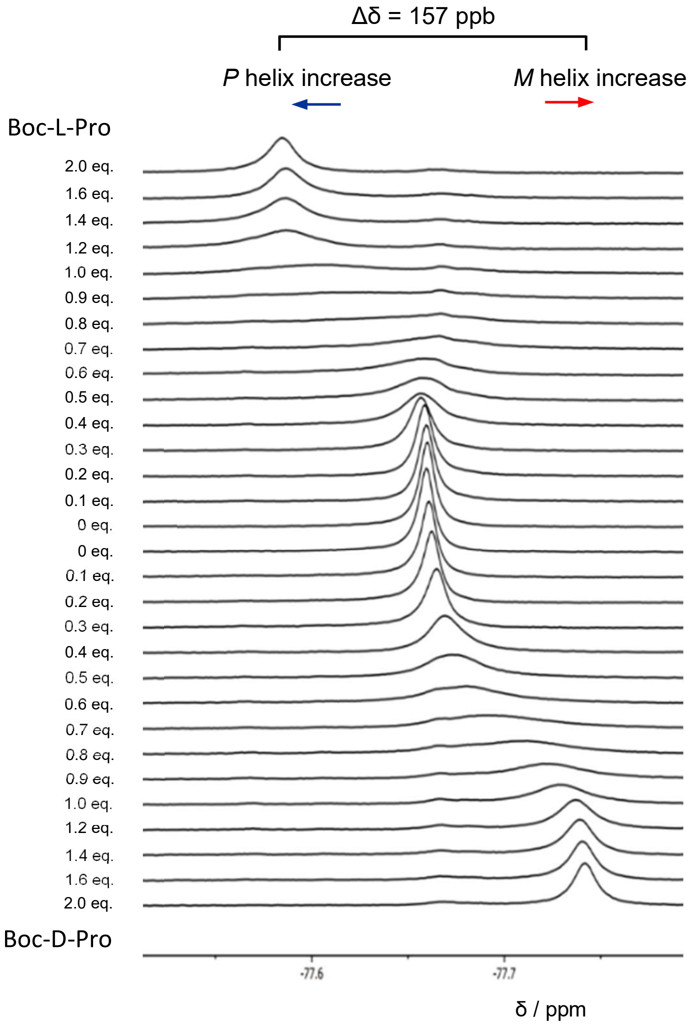

Figure S24 Partial stacked ^{19}F NMR spectra (CD_3CN, 376 MHz, 298 K) of the titration of Zn((*S*)-**3**)·$2ClO_4$ with Boc-Pro. Conditions: [Zn((*S*)-**3**)·$2ClO_4$] = 4.25 mM, [Boc-D-Pro] or [Boc-L-Pro] = 47 mM, 0–2 equiv, [2,6-lutidine] = 0–2.4 equiv.

4.5 Host: Zn((*R*)-4)·2ClO$_4$

4.5.1 Titration with Boc-Pro/2,6-lutidine (^{1}H NMR)

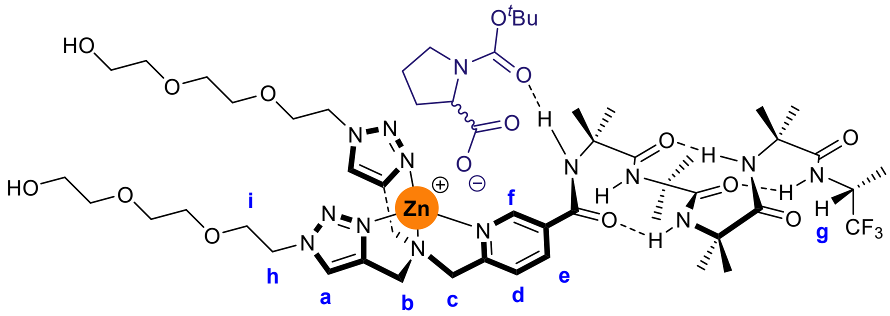

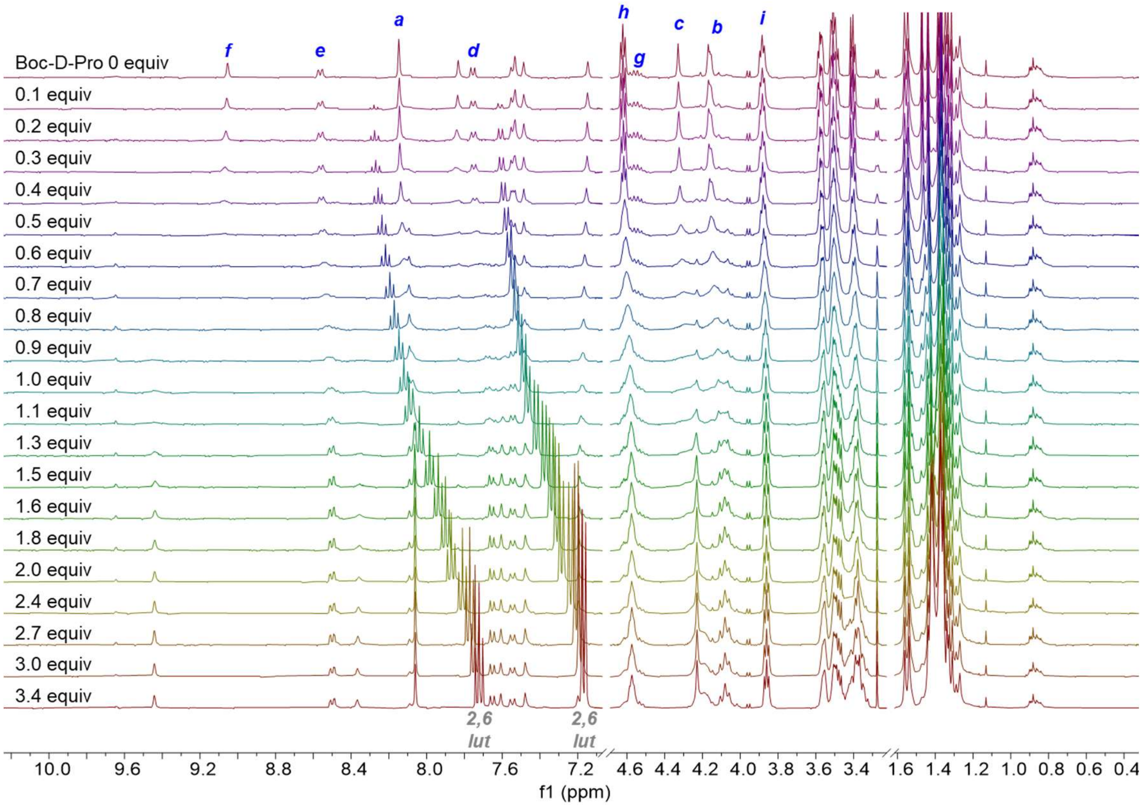

Figure S25 Partial stacked ^{1}H NMR (CD_3CN, 400 MHz, 298 K) of the titration of Zn((*R*)-**4**)·2ClO$_4$ with Boc-D-Pro. Conditions: [Zn((*R*)-**4**)·2ClO$_4$] = 2 mM, [Boc-D-Pro] = 0–3.4 equiv, [2,6-lutidine] = 0–4.2 equiv.

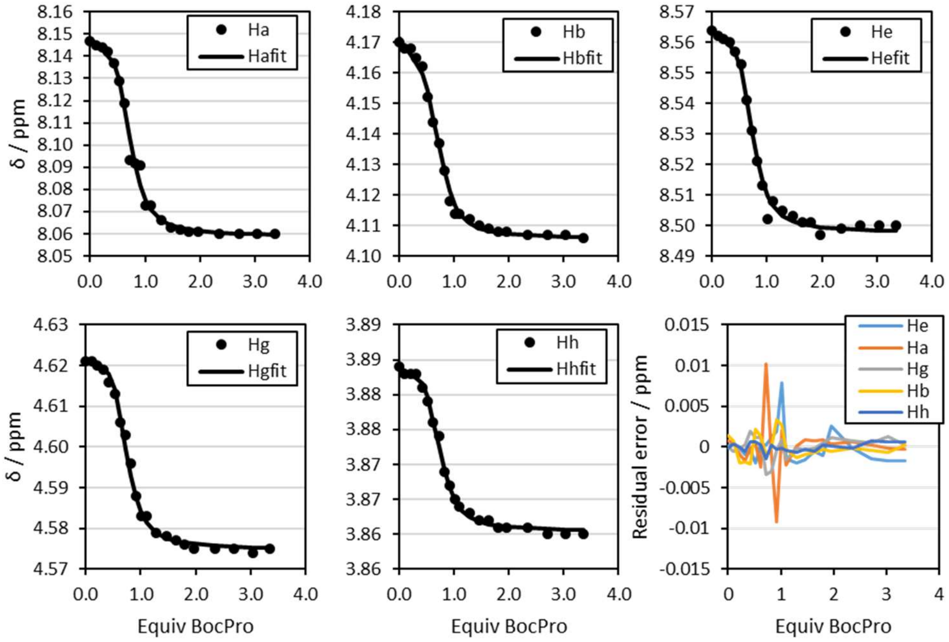

Figure S26 Global fitting of protons H_a, H_b, H_e, H_g, H_h (labelling from Figure S25) and related residual errors with SupraFit. Model: 2:1 host/guest. Conditions. [Zn((*R*)-**4**)·2ClO$_4$] = 2 mM, [Boc-D-Pro] = 0–3.4 equiv, [2,6-lutidine] = 0–4.2 equiv, CD_3CN, 298 K.

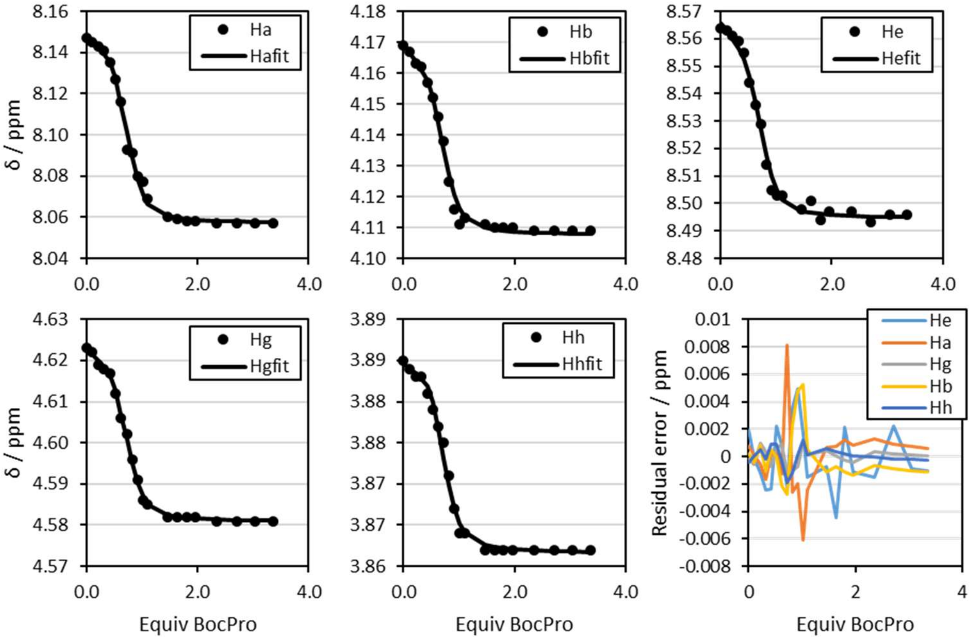

Figure S27 Global fitting of protons H_a, H_b, H_e, H_g, H_h (labelling from Figure S25), and related residual errors with SupraFit. Model: 2:1 host/guest. Conditions. [Zn((*R*)-**4**)·2ClO$_4$] = 2 mM, [Boc-L-Pro] = 0–3.4 equiv, [2,6-lutidine] = 0–4.2 equiv, CD_3CN, 298 K.

Table S1 Summary of K_{11} and K_{21} binding constants calculated from ^{1}H NMR titrations (CD_3CN, 400 MHz, 298 K) of [Zn((*R*)-**4**)·2ClO$_4$] (2 mM) with D or L Boc-Pro (0–3.4 equiv) and 2,6-lutidine (0–4.2 equiv). Results from two independent repetitions from different batches of host.

**Guest**	**K_{11} / M^{-1}**	**K_{21} / M^{-1}**
**+ Boc-D-Pro**	1.5×10^6	3.1×10^4
	9.9×10^5	1.4×10^4
**+ Boc-L-Pro**	7.1×10^7	6.3×10^5
	1.5×10^6	2.1×10^4

4.5.2 Titration with Boc-Pro/2,6-lutidine (^{19}F NMR)

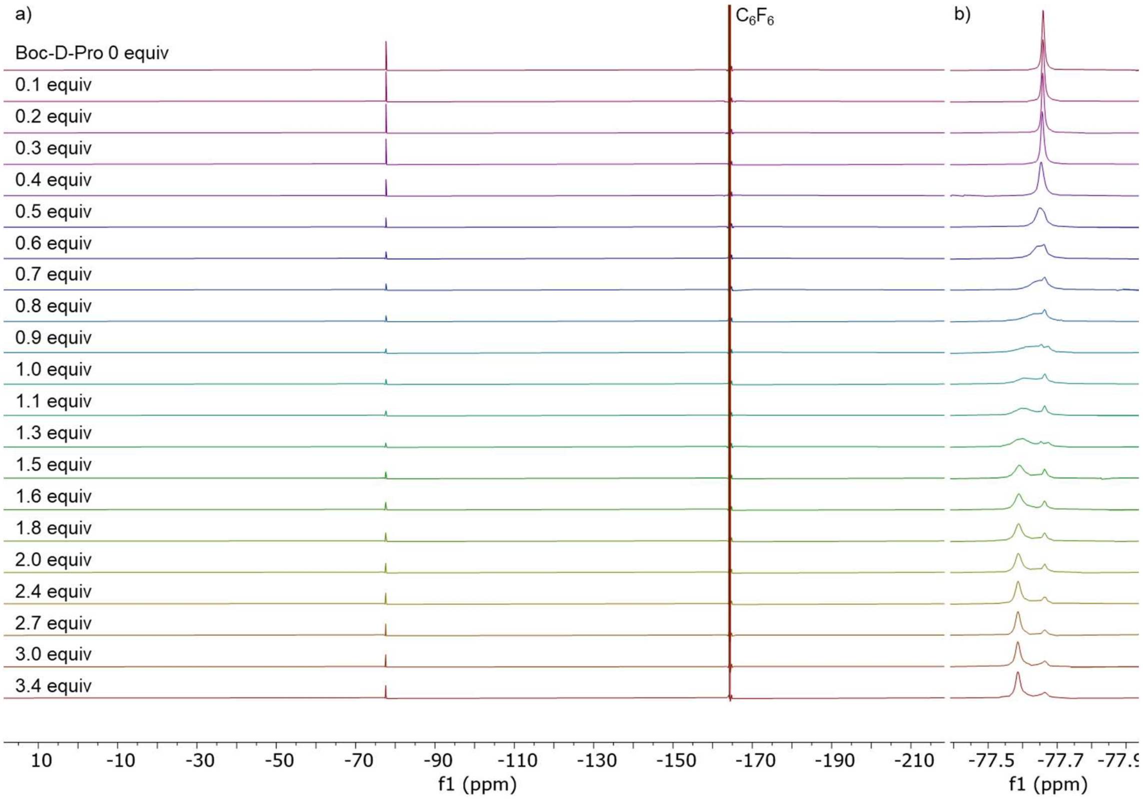

Figure S28 (a) Full sweep width and (b) expanded stacked ^{19}F NMR (CD_3CN, 376 MHz, 298 K) of the titration of Zn((*R*)-**4**)·$2ClO_4$ with Boc-D-Pro. Conditions: [Zn((*R*)-**4**)·$2ClO_4$] = 2 mM, [Boc-D-Pro] = 0–3.4 equiv, [2,6-lutidine] = 0–4.2 equiv. Spectra referenced with C_6F_6 at –164.38 ppm.

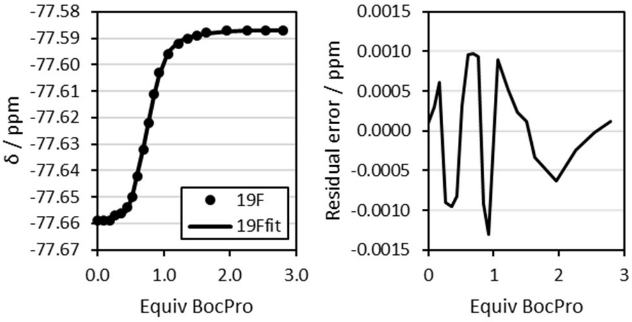

Figure S29 Fitting of the ^{19}F peak and related residual errors with SupraFit. Model: 2:1 host/guest. Conditions. [Zn((*R*)-**4**)·$2ClO_4$] = 2 mM, [Boc-D-Pro] = 0–3.4 equiv, [2,6-lutidine] = 0–4.2 equiv, CD_3CN, 298 K.

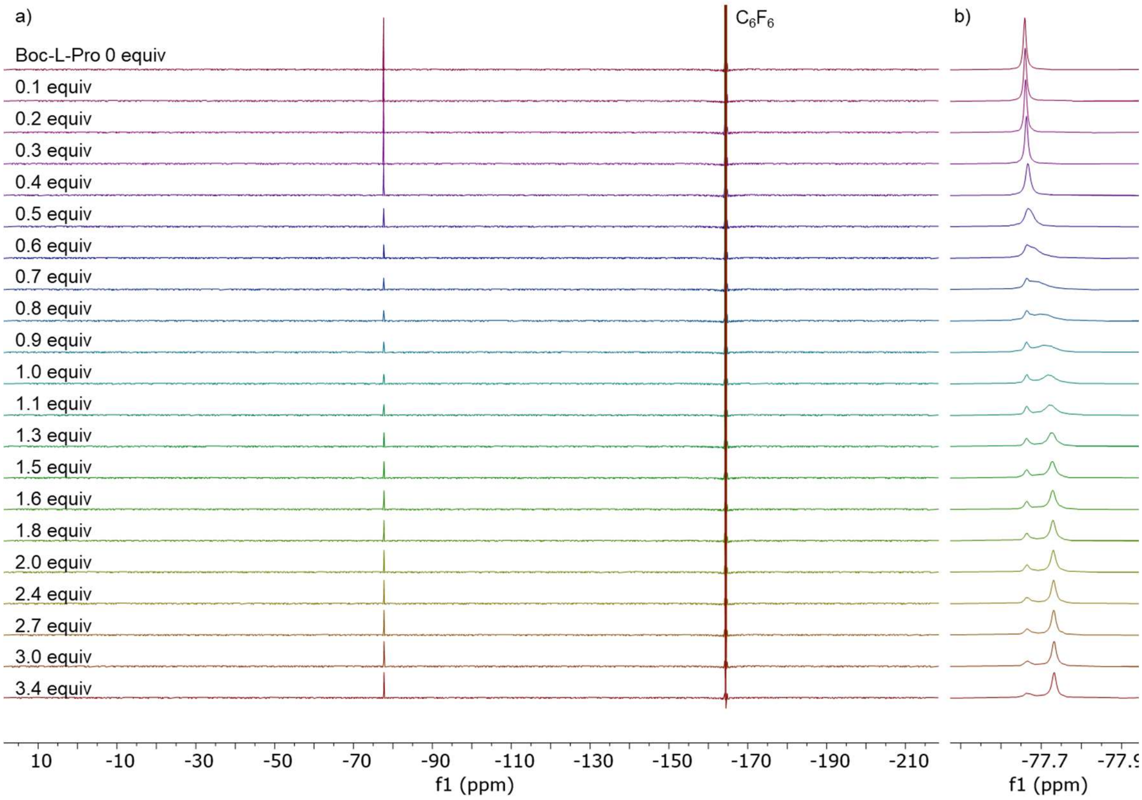

Figure S30 (a) Full sweep width and (b) expanded stacked ^{19}F NMR (CD_3CN, 376 MHz, 298 K) of the titration of Zn((*R*)-**4**)·2ClO$_4$ with Boc-L-Pro. Conditions: [Zn(**1**)·2ClO$_4$] = 2 mM, [Boc-L-Pro] = 0–3.4 equiv, [2,6-lutidine] = 0–4.2 equiv. Spectra referenced with C_6F_6 at –164.38 ppm.

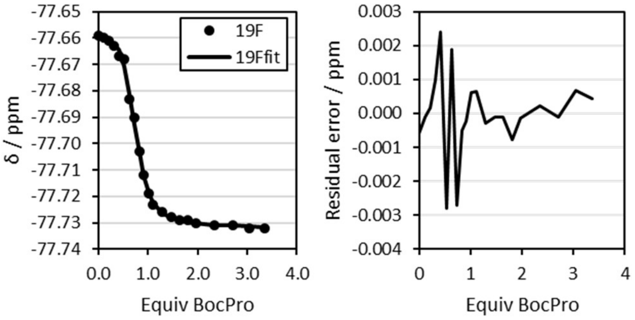

Figure S31 Fitting of the ^{19}F peak and related residual errors with SupraFit. Model: 2:1 host/guest. Conditions. [Zn((*R*)-**4**)·2ClO$_4$] = 2 mM, [Boc-L-Pro] = 0–3.4 equiv, [2,6-lutidine] = 0–4.2 equiv, CD_3CN, 298 K.

Table S2 Summary of K_{11} and K_{21} binding constants calculated from ^{19}F NMR titrations (CD_3CN, 376 MHz, 298 K) of [Zn((*R*)-**4**)·2ClO$_4$] (2 mM) with D or L Boc-Pro (0–3.4 equiv) and 2,6-lutidine (0–4.2 equiv). Results from two independent repetitions from different batches of host.

Guest	K_{11} / M^{-1}	K_{21} / M^{-1}
**+ Boc-D-Pro**	1.5×10^6	1.6×10^4
	5.1×10^6	3.7×10^4
**+ Boc-L-Pro**	8.5×10^5	1.4×10^4
	1.6×10^7	6.4×10^4

4.5.3 Titration with tetrabutylammonium acetate (^{1}H NMR)

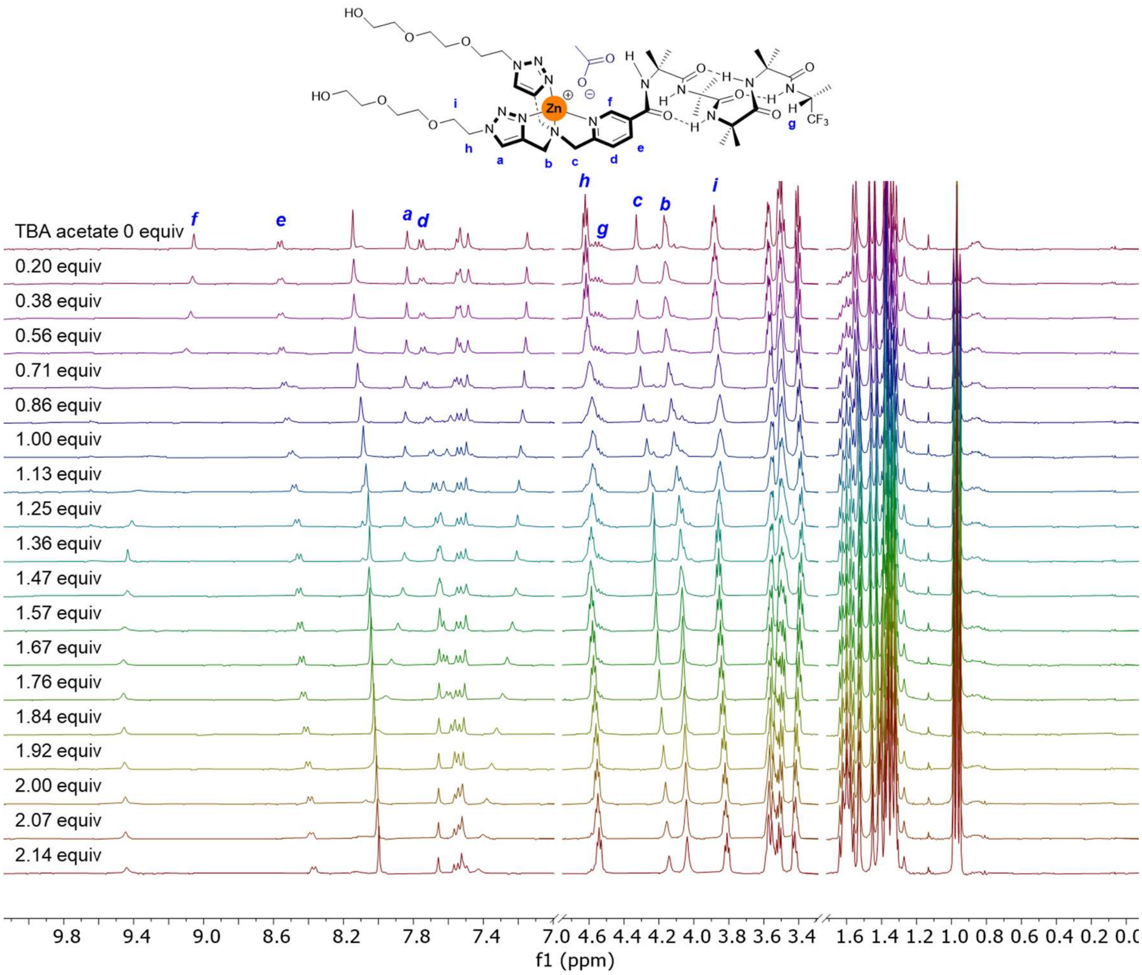

Figure S32 Partial stacked ^{1}H NMR (CD_3CN, 400 MHz, 298 K) of Zn((*R*)-**2**)·$2ClO_4$ with tetrabutylammonium acetate. Conditions: [Zn((*R*)-**4**)·$2ClO_4$] = 2 mM, [Boc-D-Pro] = 0–2.1 equiv.

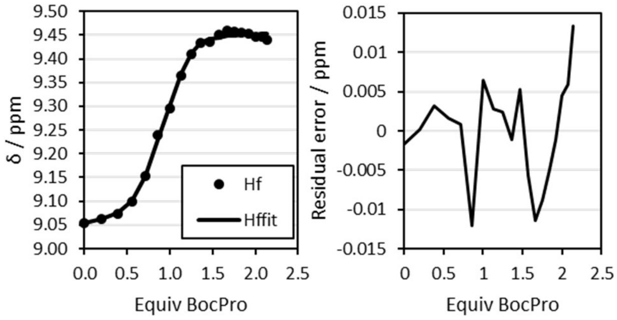

Figure S33 Fitting of protons H_f (labelling from Figure S25) and related residual errors with SupraFit. Model: 2:1 host/guest. Conditions. [Zn((*R*)-**4**)·$2ClO_4$] = 2 mM, [Boc-D-Pro] = 0–2.1 equiv, CD_3CN, 298 K. $K_{11} = 2.2 \times 10^6$ M^{-1}, $K_{21} = 9.3 \times 10^3$ M^{-1}.

4.5.4 Titration with tetrabutylammonium acetate (^{19}F NMR)

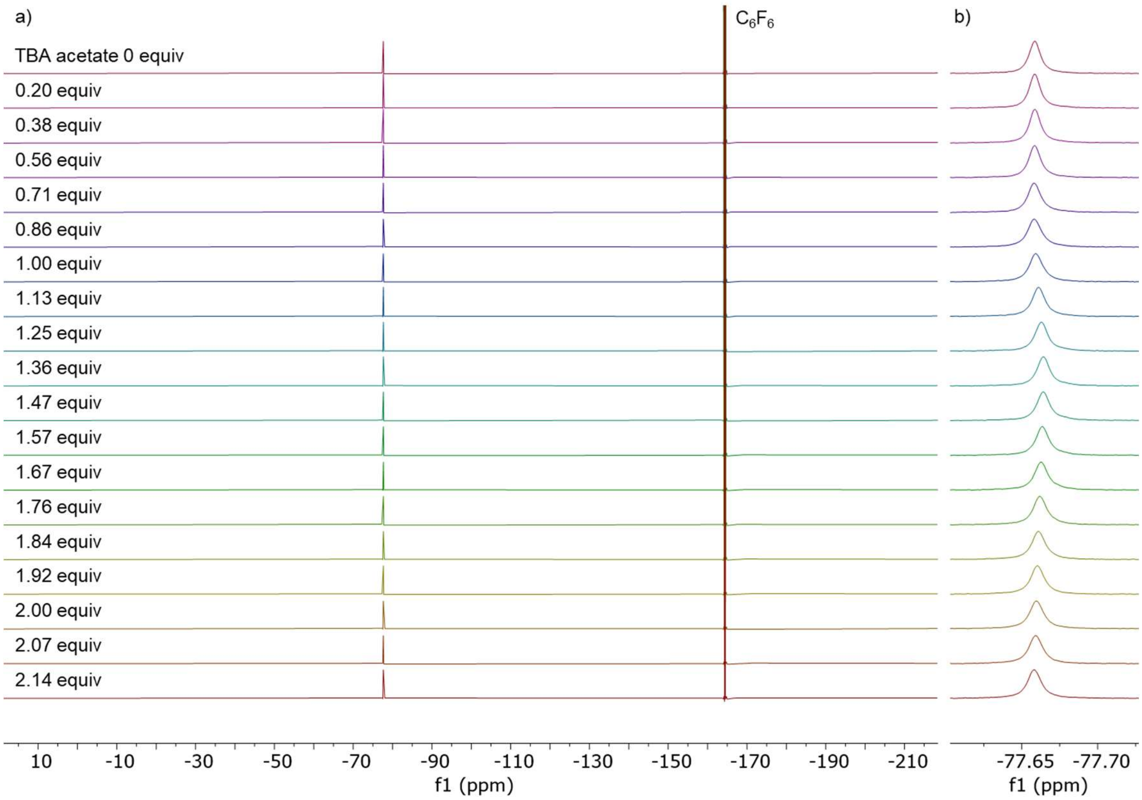

Figure S34 (a) Full sweep width and (b) expanded stacked ^{19}F NMR (CD_3CN, 376 MHz, 298 K) of Zn((*R*)-**4**)·2ClO$_4$ with tetrabutylammonium acetate. Conditions: [Zn((*R*)-**4**)·2ClO$_4$] = 2 mM, [acetate] = 0–2.1 equiv. Spectra referenced with C_6F_6 at –164.38 ppm.

4.5.5 Control: titration with 2,6-lutidine (^{1}H NMR)

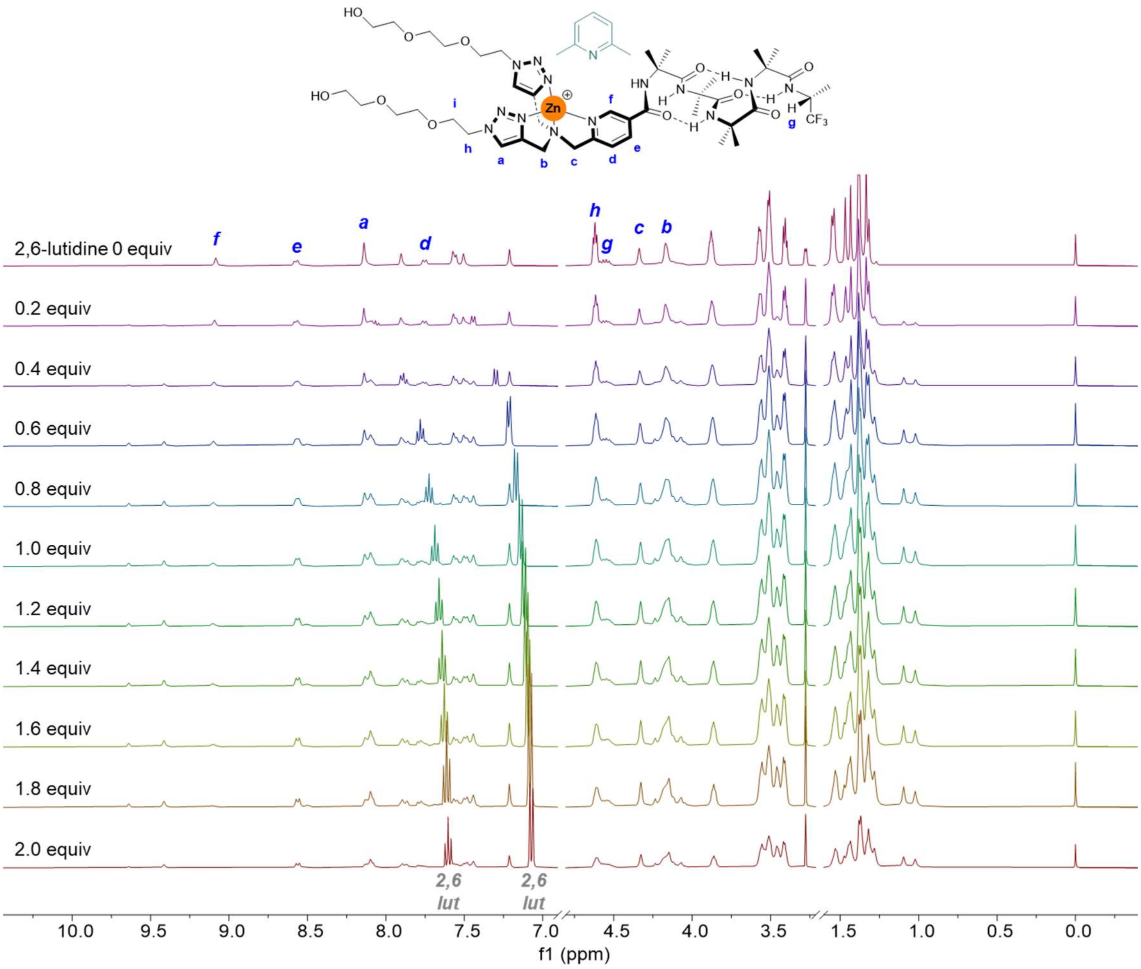

Figure S35 Partial stacked ^{1}H NMR (CD_3CN, 400 MHz, 298 K) of the titration of Zn((*R*)-**4**)·2ClO$_4$ with 2,6-lutidine. Conditions: [Zn((*R*)-**4**)·2ClO$_4$] = 2 mM, [2,6-lutidine] = 0–2.0 equiv.

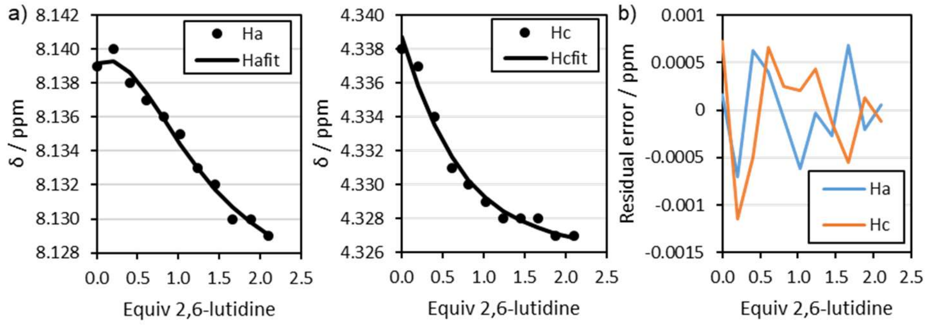

Figure S36 Global fitting of protons H$_a$ and H$_c$ (labelling from Figure S35) (a) with software SupraFit and related residual errors (c). Model: 2:1 host/guest. K_{11} = 2.2 × 10^2 M^{-1}, K_{21} = 2.0 × 10^0 M^{-1}.

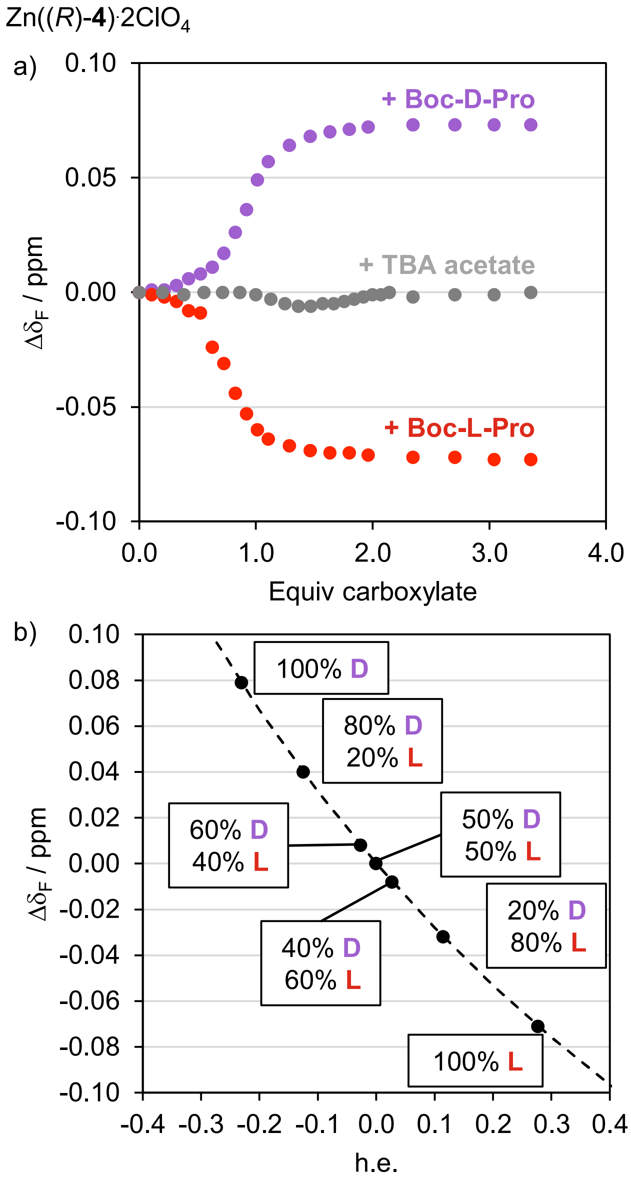

Figure S37 a) ^{19}F shift of Zn((*R*)-**4**)·2ClO$_4$ in the presence of increasing concentrations of Boc-D-Pro (purple), tetrabutylammonium (TBA) acetate (grey) and Boc-L-Pro (red). b) Nonlinear correlation between the ^{19}F shift of Zn((*R*)-**4**)·2ClO$_4$ and the helical excess (h.e.) in the presence of scalemic mixtures of the two enantiomers of Boc-Pro. Conditions: [Zn((*R*)-**4**)·2ClO$_4$] = 2 mM, [Boc-Pro] = 0–6.7 mM, [2,6-lutidine] = 0–7.2 mM. Spectra referenced with C_6F_6 at –164.38 ppm.[S2,S12]

4.6 Host: Zn((*R*)-5)·2ClO$_4$

4.6.1 Titration with Boc-Pro/2,6-lutidine (^{1}H NMR)

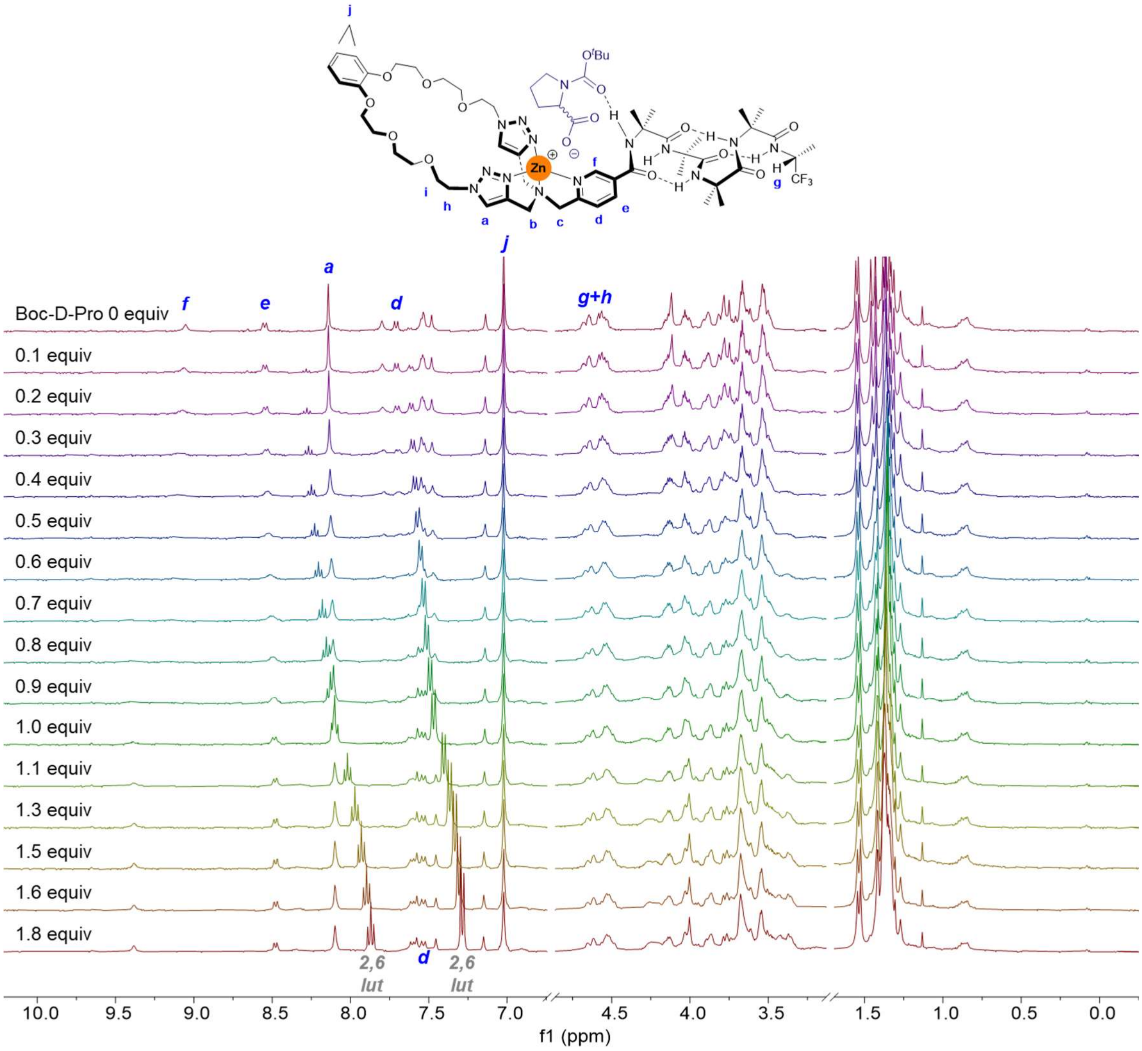

Figure S38 Partial stacked ^{1}H NMR (CD_3CN, 400 MHz, 298 K) of Zn((*R*)-**5**)·2ClO$_4$ with Boc-D-Pro. Conditions: [Zn((*R*)-**5**)·2ClO$_4$] = 2 mM, [Boc-D-Pro] = 0–1.8 equiv, [2,6-lutidine] = 0–2.2 equiv.

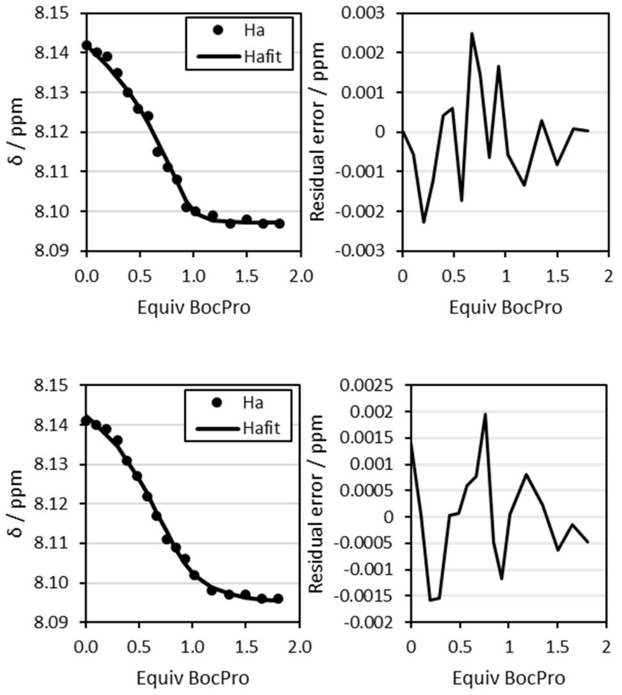

Figure S39 Fitting of proton H_a (labelling from Figure S38) and related residual errors with SupraFit for the titration with Boc-D-Pro (top row) and Boc-L-Pro (bottom row). Model: 2:1 host/guest. Conditions. [Zn((*R*)-**5**)·2ClO$_4$] = 2 mM, [Boc-D-Pro] = 0–1.8 equiv, [2,6-lutidine] = 0–2.2 equiv.

Table S3 Summary of K_{11} and K_{21} binding constants calculated from ^{1}H NMR titrations (CD_3CN, 400 MHz, 298 K) of [Zn((*R*)-**5**)·2ClO$_4$] (2 mM) with D or L Boc-Pro (0–1.8 equiv) and 2,6-lutidine (0–2.2 equiv). Results from two independent repetitions from different batches of host.

Guest	K_{11} / M^{-1}	K_{21} / M^{-1}
+ Boc-D-Pro	$2.0×10^6$	$1.7×10^4$
	$1.0×10^6$	$2.1×10^3$
+ Boc-L-Pro	$4.1×10^6$	$5.9×10^4$
	$1.1×10^5$	$1.9×10^3$

4.6.2 Titration with Boc-Pro/2,6-lutidine (^{19}F NMR)

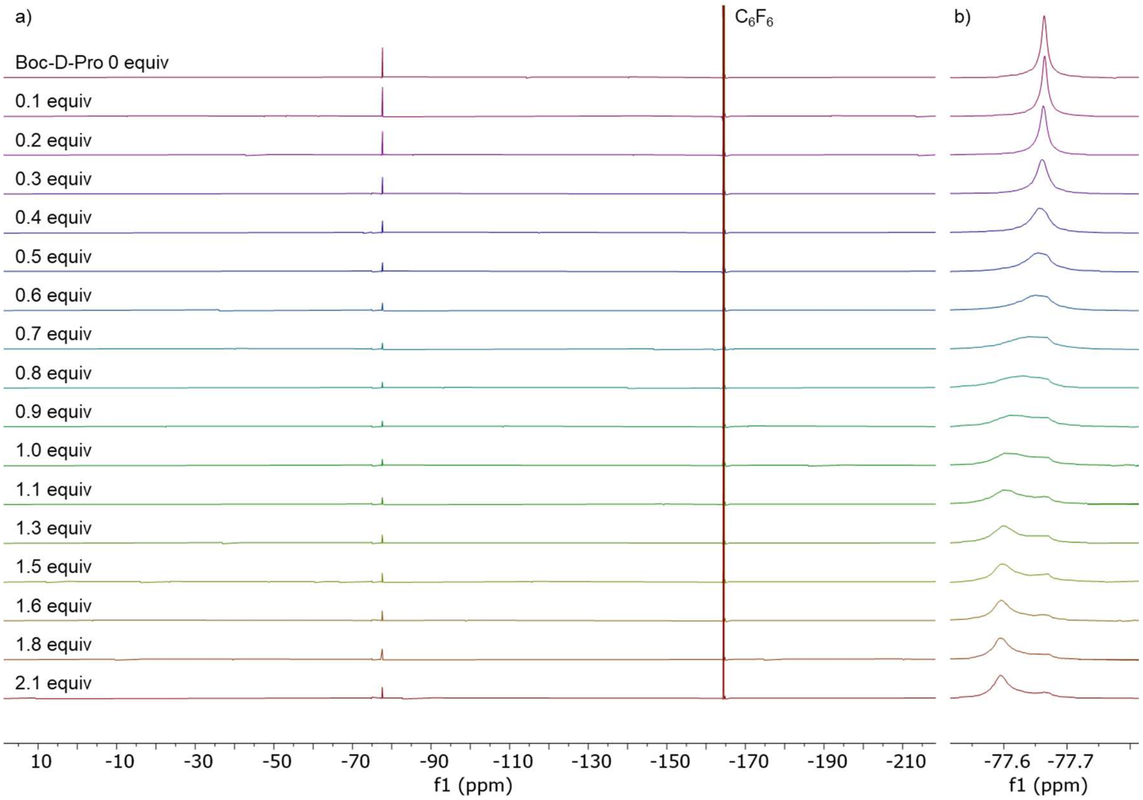

Figure S40 (a) Full sweep width and (b) expanded stacked ^{19}F NMR (CD_3CN, 376 MHz, 298 K) for the titration of Zn((*R*)-**5**)·$2ClO_4$ with Boc-D-Pro. Conditions: [Zn((*R*)-**5**)·$2ClO_4$] = 2 mM, [Boc-L-Pro] = 0–1.8 equiv, [2,6-lutidine] = 0–2.2 equiv. Spectra referenced with C_6F_6 at –164.38 ppm.

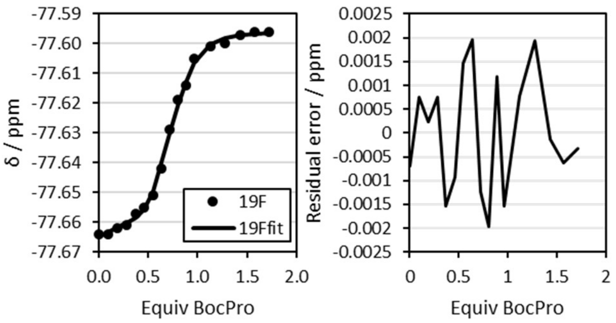

Figure S41 Fitting of the ^{19}F peak and related residual errors with SupraFit. Model: 2:1 host/guest. Conditions. [Zn((*R*)-**5**)·$2ClO_4$] = 2 mM, [Boc-D-Pro] = 0–1.8 equiv, [2,6-lutidine] = 0–2.2 equiv, CD_3CN, 298 K.

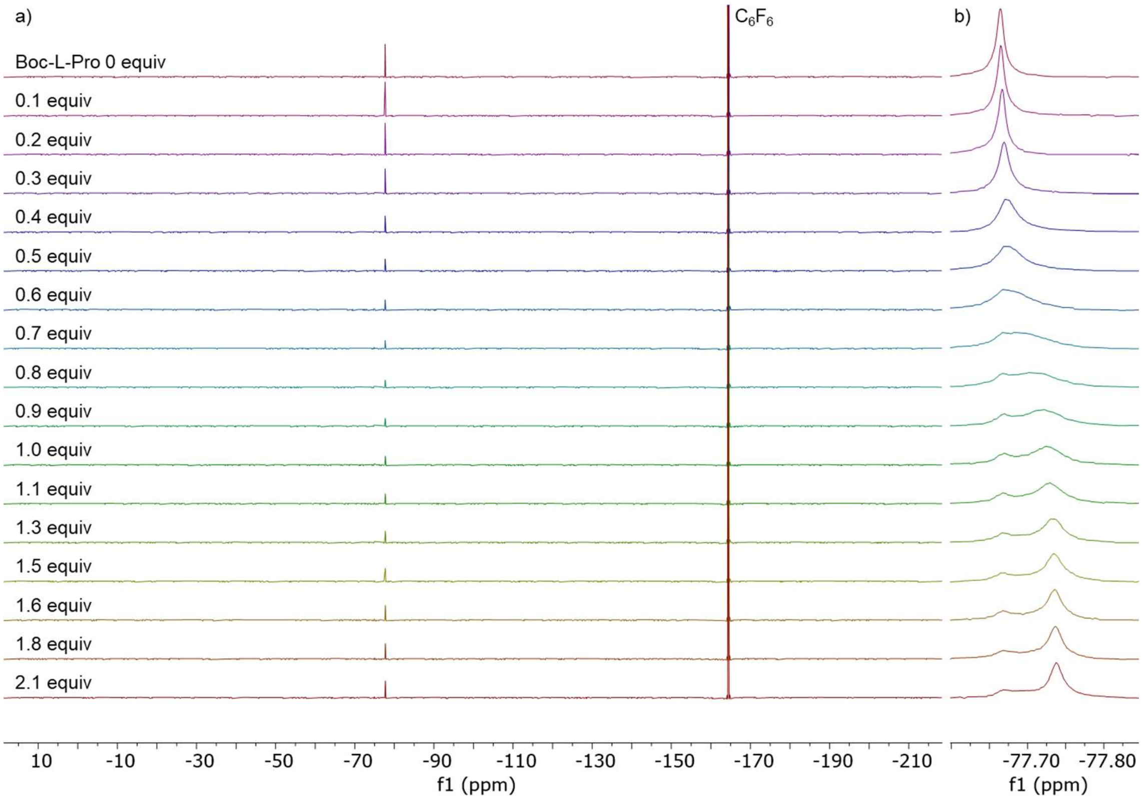

Figure S42 (a) Full sweep width and (b) expanded stacked ^{19}F NMR (CD_3CN, 376 MHz, 298 K) of the titration of Zn((*R*)-**5**)·$2ClO_4$ with Boc-L-Pro. Conditions: [Zn((*R*)-**5**)·$2ClO_4$] = 2 mM, [Boc-L-Pro] = 0–1.8 equiv, [2,6-lutidine] = 0–2.2 equiv. Spectra referenced with C_6F_6 at –164.38 ppm.

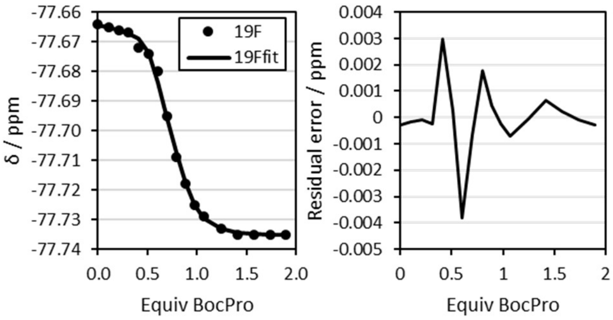

Figure S43 Fitting of the ^{19}F peak and related residual errors with SupraFit. Model: 2:1 host/guest. Conditions. [Zn((*R*)-**5**)·$2ClO_4$] = 2 mM, [Boc-L-Pro] = 0–1.8 equiv, [2,6-lutidine] = 0–2.2 equiv, CD_3CN, 298 K.

Table S4 Summary of K_{11} and K_{21} binding constants calculated from ^{19}F NMR titrations (CD_3CN, 376 MHz, 298 K) of [Zn((*R*)-**5**)·$2ClO_4$] (2 mM) with D or L Boc-Pro (0–1.8 equiv) and 2,6-lutidine (0–2.2 equiv). Results from two independent repetitions from different batches of host.

**Guest**	**K_{11} / M^{-1}**	**K_{21} / M^{-1}**
**+ Boc-D-Pro**	3.3×10^6	3.8×10^5
	5.5×10^6	4.7×10^4
**+ Boc-L-Pro**	2.4×10^7	3.1×10^5
	4.5×10^6	3.4×10^4

4.7 Host: Zn((*R*)-6)·2ClO$_4$

4.7.1 Titration with Boc-Pro/2,6-lutidine (^{1}H NMR)

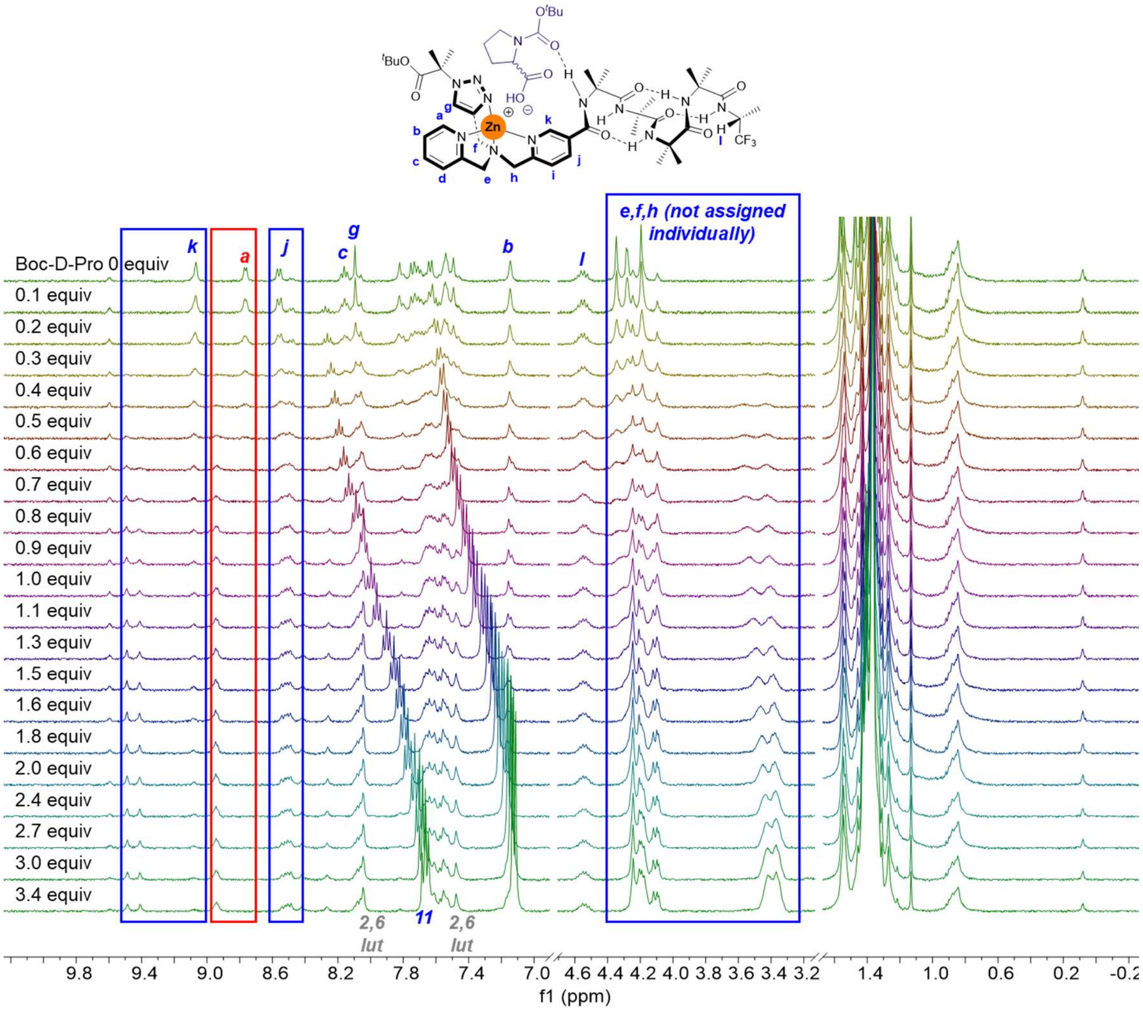

Figure S44 Partial stacked ^{1}H NMR (CD$_3$CN, 400 MHz, 298 K) of the titration of Zn((*R*)-**6**)·2ClO$_4$ with Boc-D-Pro. Conditions: [Zn((*R*)-**6**)·2ClO$_4$] = 2 mM, [Boc-D-Pro] = 0–3.4 equiv, [2,6-lutidine] = 0–4.2 equiv.

4.7.2 Titration with Boc-Pro/2,6-lutidine (^{19}F NMR)

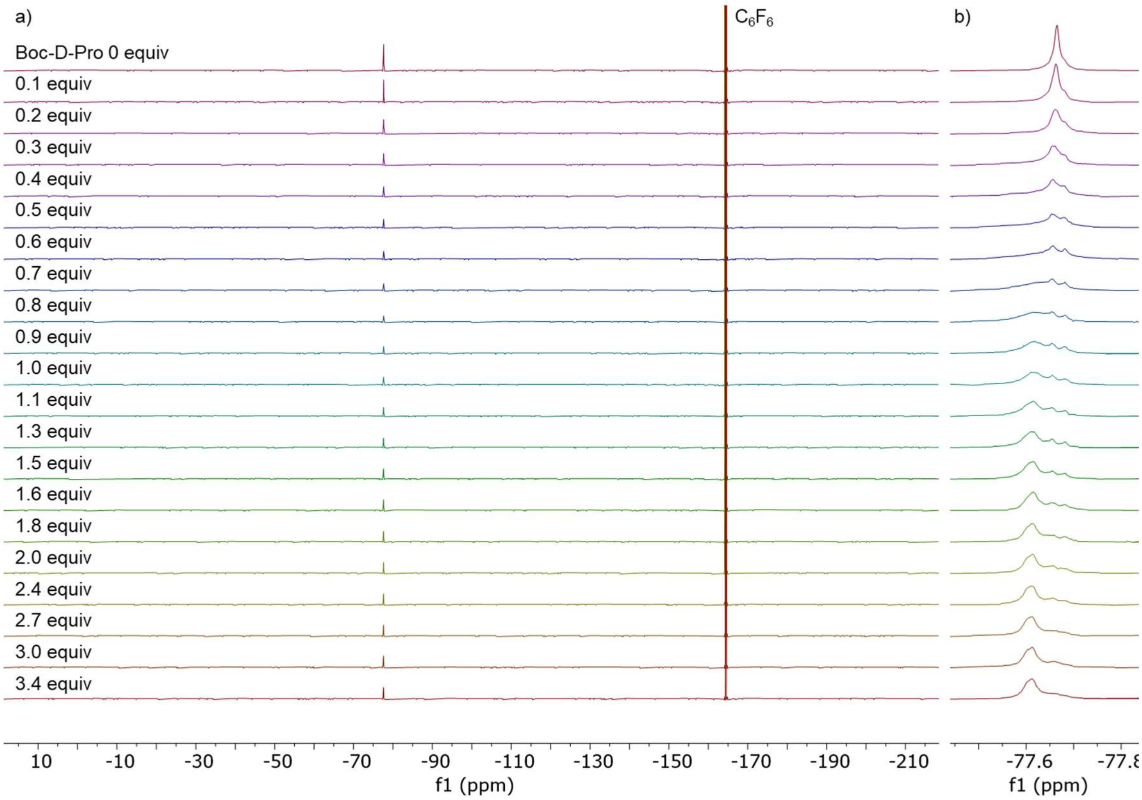

Figure S45 (a) Full sweep width and (b) expanded stacked ^{19}F NMR (CD_3CN, 376 MHz, 298 K) of the titration of Zn((*R*)-**6**)·2ClO$_4$ with Boc-D-Pro. Conditions: [Zn((*R*)-**6**)·2ClO$_4$] = 2 mM, [Boc-D-Pro] = 0–3.4 equiv, [2,6-lutidine] = 0–4.2 equiv. Spectra referenced with C_6F_6 at –164.38 ppm.

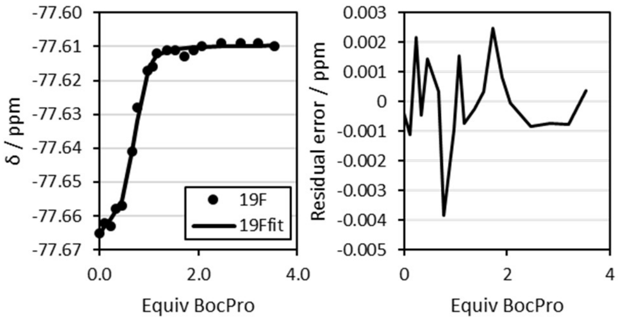

Figure S46 Fitting of the ^{19}F peak and related residual errors with SupraFit. Model: 2:1 host/guest. Conditions. [Zn((*R*)-**6**)·2ClO$_4$] = 2 mM, [Boc-D-Pro] = 0–3.4 equiv, [2,6-lutidine] = 0–4.2 equiv, CD_3CN, 298 K.

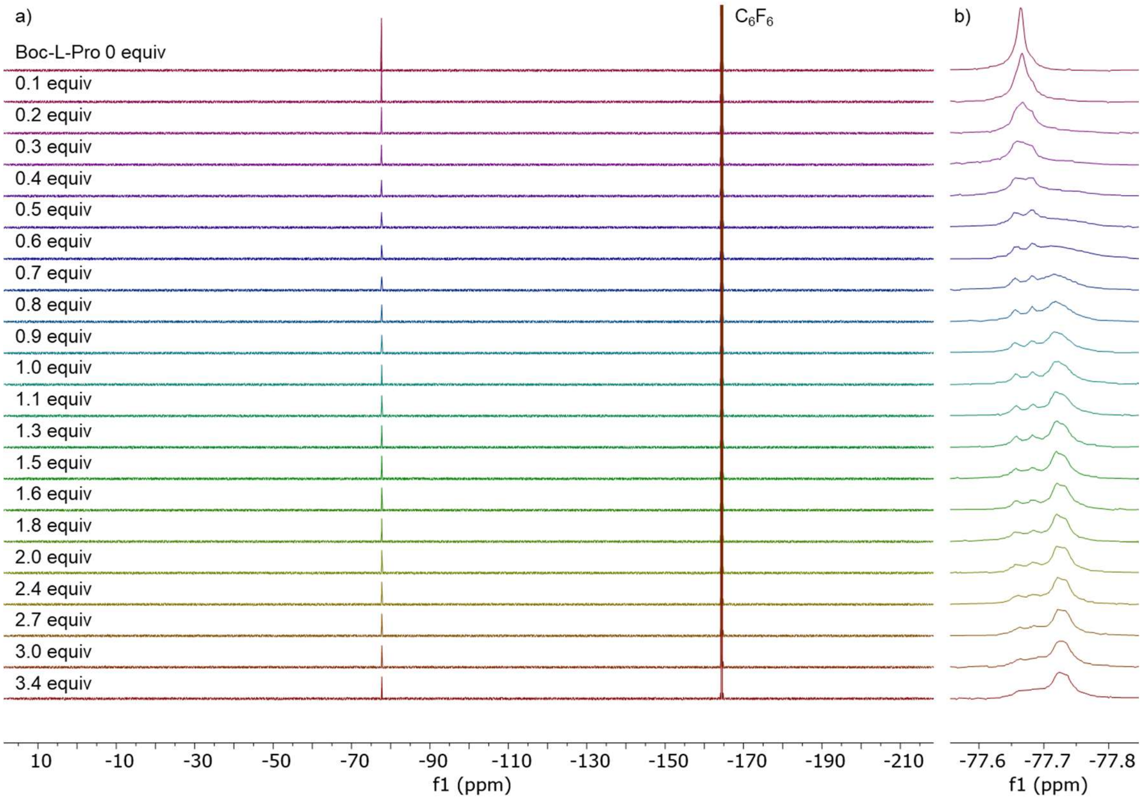

Figure S47 (a) Full sweep width and (b) expanded stacked ^{19}F NMR (CD_3CN, 376 MHz, 298 K) of the titration of Zn((*R*)-**6**)·$2ClO_4$ with Boc-L-Pro. Conditions: [Zn((*R*)-**6**)·$2ClO_4$] = 2 mM, [Boc-D-Pro] = 0–3.4 equiv, [2,6-lutidine] = 0–4.2 equiv. Spectra referenced with C_6F_6 at –164.38 ppm.

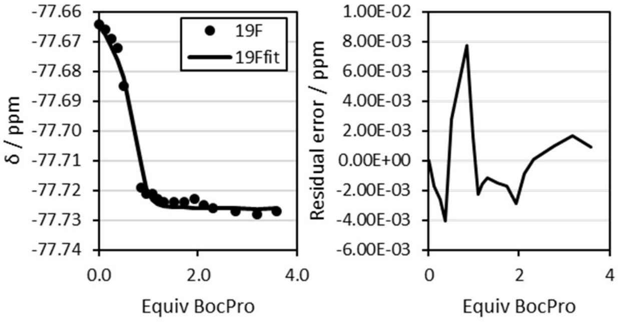

Figure S48 Fitting of the ^{19}F peak and related residual errors with SupraFit. Model: 2:1 host/guest. Conditions. [Zn((*R*)-**6**)·$2ClO_4$] = 2 mM, [Boc-L-Pro] = 0–3.4 equiv, [2,6-lutidine] = 0–4.2 equiv, CD_3CN, 298 K.

Table S5 Summary of K_{11} and K_{21} binding constants calculated from ^{19}F NMR titrations (CD_3CN, 376 MHz, 298 K) of [Zn((*R*)-**6**)·$2ClO_4$] (2 mM) with D or L Boc-Pro (0–3.4 equiv) and 2,6-lutidine (0–4.2 equiv). Results from two independent repetitions from different batches of host.

Guest	K_{11} / M^{-1}	K_{21} / M^{-1}
+ Boc-D-Pro	7.6×10^6	4.9×10^4
	5.8×10^6	5.0×10^4
+ Boc-L-Pro	3.1×10^6	1.2×10^4
	2.3×10^6	1.3×10^4

4.7.3 Control: Zn((*R*)-6)·2ClO$_4$ with an excess of Boc-L-Pro and 2,6-lutidine

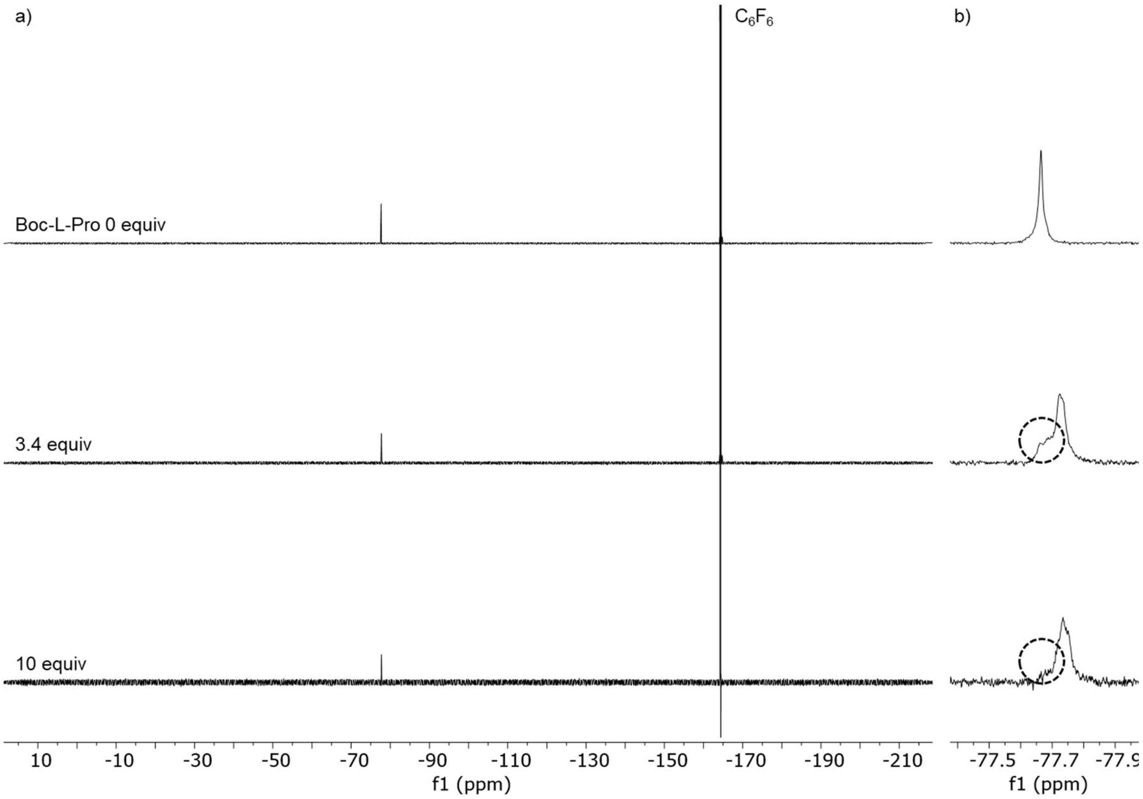

Figure S49 (a) Full sweep width and (b) expanded stacked ^{19}F NMR (CD_3CN, 376 MHz, 298 K) of Zn((*R*)-**6**)·2ClO$_4$ in the presence of a moderate (middle) and a large (bottom) excess of Boc-L-Pro. Conditions: [Zn((*R*)-**6**)·2ClO$_4$] = 2 mM, [Boc-L-Pro] = 0, 3.4, and 10 equiv, [2,6-lutidine] = 0, 4.2, and 12 equiv. Spectra referenced with C_6F_6 at –164.38 ppm.

5. Modelling of the ^{19}F chemical shift in CD_3CN

The effects of ligand binding on the TFEA reporter CF_3 signal for both Zn((*R*)-**2**)·$2ClO_4$ and Zn((*S*)-**2**)·$2ClO_4$ are plotted in **Figure S50**. With binding of both Boc-Pro and Boc-Pip, rapid exchange between the *P*- and *M*-helix states is observed, with the CF_3 signal gradually moving with increased helical bias induced with increasing ligand titrated. Binding of TiPSY appears to result in slow exchange of the two conformers on the ^{19}F NMR spectroscopy timescale, with distinct peaks of the reporter in two environments visible, one decreasing in intensity as the other increases. It is apparent that the effects of ligands bound at receptor Zn((*R*)-**2**)·$2ClO_4$ are mirrored in Zn((*S*)-**2**)·$2ClO_4$. Comparing the direction of induced ^{19}F NMR chemical shift upon ligand binding at Zn((*R*)-**2**)·$2ClO_4$ to the covalently controlled (*R*)-TFEA terminated foldamers reported by Wang *et al.*, allows the enriched screw-sense to be determined.[S5] *P*-helix enrichment, observed with binding of Boc-L-Pro, Boc-L-Pip and (*R*)-TiPSY, results in an upfield shift in (*R*)-TFEA terminated receptor and a downfield shift for (*S*)-TFEA receptor. Conversely, *M*-enrichment is observed upon binding of Boc-D-Pro, Boc-D-Pip and (*S*)-TiPSY, resulting in a downfield shift in Zn((*R*)-**2**)·$2ClO_4$ and an upfield shift for Zn((*S*)-**2**)·$2ClO_4$.

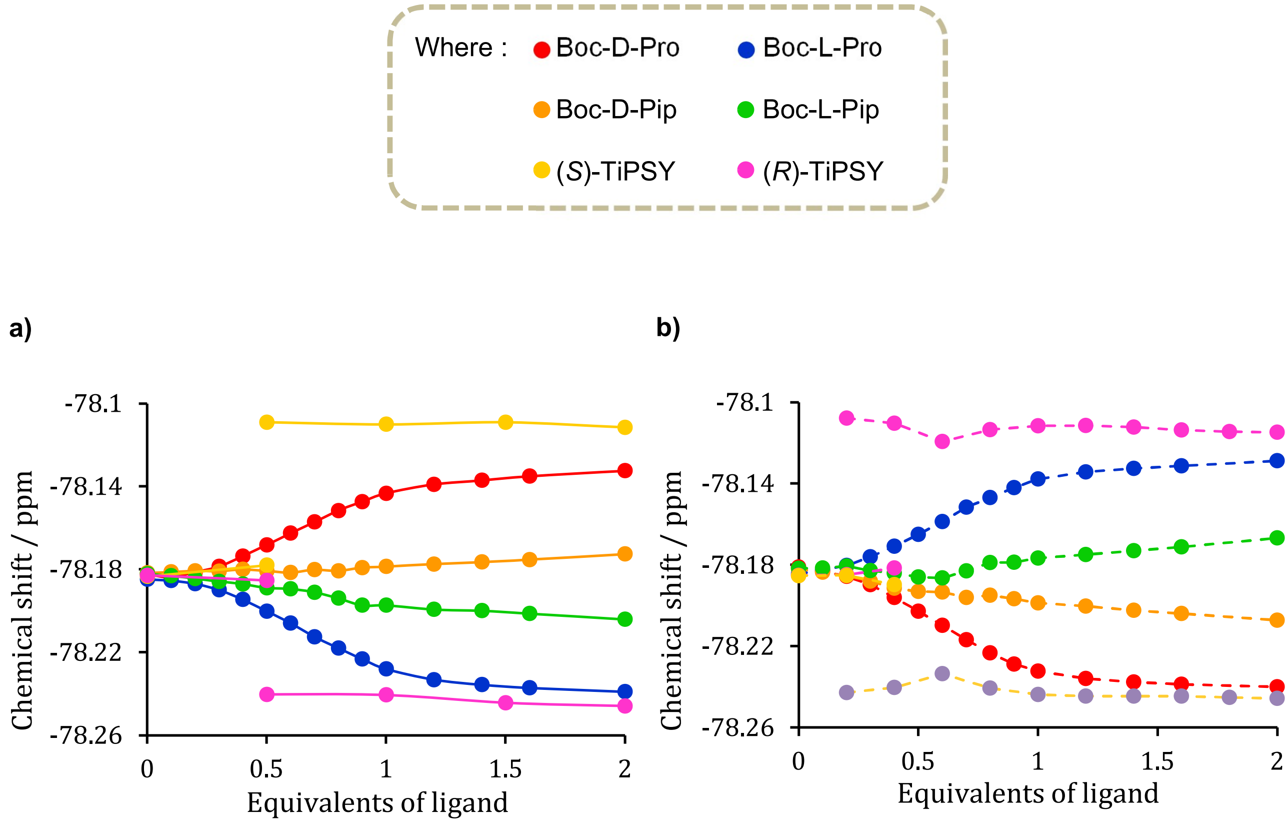

Figure S50 Titrations of enantiomeric pairs of ligands BocPro, BocPip or TiPSY (47 mM) with Zn((*R*)-**2**)·$2ClO_4$ and Zn((*S*)-**2**)·$2ClO_4$ (4.25 mM) in CD_3CN. Plots of TFEA CF_3 chemical shift with increasing equivalents of ligands in a) Zn((*R*)-**2**)·$2ClO_4$ (solid line) and b) Zn((*S*)-**2**)·$2ClO_4$ (dotted line).

Table S6 ^{19}F NMR chemical shift differences between ligand-bound diastereomers at 2 eq., extracted from Figure S50.

Reporter	Ligand	Δδ between diastereoisomers / ppb
Zn((*R*)-**2**)·2ClO$_4$	Boc-Pro	107
	Boc-Pip	32
	TiPSY	134
Zn((*S*)-**2**)·2ClO$_4$	Boc-Pro	111
	Boc-Pip	40
	TiPSY	131

The magnitude of the bias in helix populations (*P/M*) induced by a controller, be that covalent or a ligand, can be quantified and presented as an inferred helical excess (h.e.$_0$), giving a value for the induced enrichment, adjacent to the N-terminal controller, on a scale from 100% *P*-helix to 100% *M*-helix.[S5,S13,S14] The final chemical shift values of Zn((*R*)-**2**)·2ClO$_4$ with 2 eq. of ligand were interpolated into the published curve of the h.e.$_0$ induced by covalently controlled Aib tetramers equipped with the same (*R*)-TFEA C-terminal reporter (**Figure S51**).[S5] Due to a difference of around 0.5 ppm between the chemical shifts of the uncontrolled covalent foldamer reported by Wang *et al.*[S5] and Zn((*R*)-**2**)·2ClO$_4$ with no ligand bound, chemical shift values were converted to changes in chemical shift (Δδ). The occurrence of this difference is possibly due to differences in concentration or the zinc(II) of the binding site encouraging increased aggregation. Aib tetramers with N-terminal covalent controllers of Phe, αMeVal and (αMeVal)$_2$ were found to induce an h.e.$_0$ of ±52, 68 and 95%, respectively, adjacent to the controller.[S5,S13] From this graph, it appears Boc-Pro is able to induce an h.e.$_0$ of ±21%. N. Eccles *et al*. reported the ellipticity induced at an N-terminal BQPA binding site was greater with Boc-Pip than Boc-Pro.[S6] However, from the ^{19}F NMR spectroscopy titrations performed with Zn((*R*)-**2**)·2ClO$_4$ and Zn((*S*)-**2**)·2ClO$_4$, it appears that Boc-Pip induces a considerably smaller bias on foldamers with the BTPA binding site. The average Δδ between diasteroisomeric pairs induced by 2 eq. Boc-Pip was of 36 ppb, corresponding to an h.e.$_0$ of ±7%. Binding of TiPSY resulted in an average Δδ between diasteroisomeric pairs of 133 ppb, corresponding to an h.e.$_0$ of ±23%.

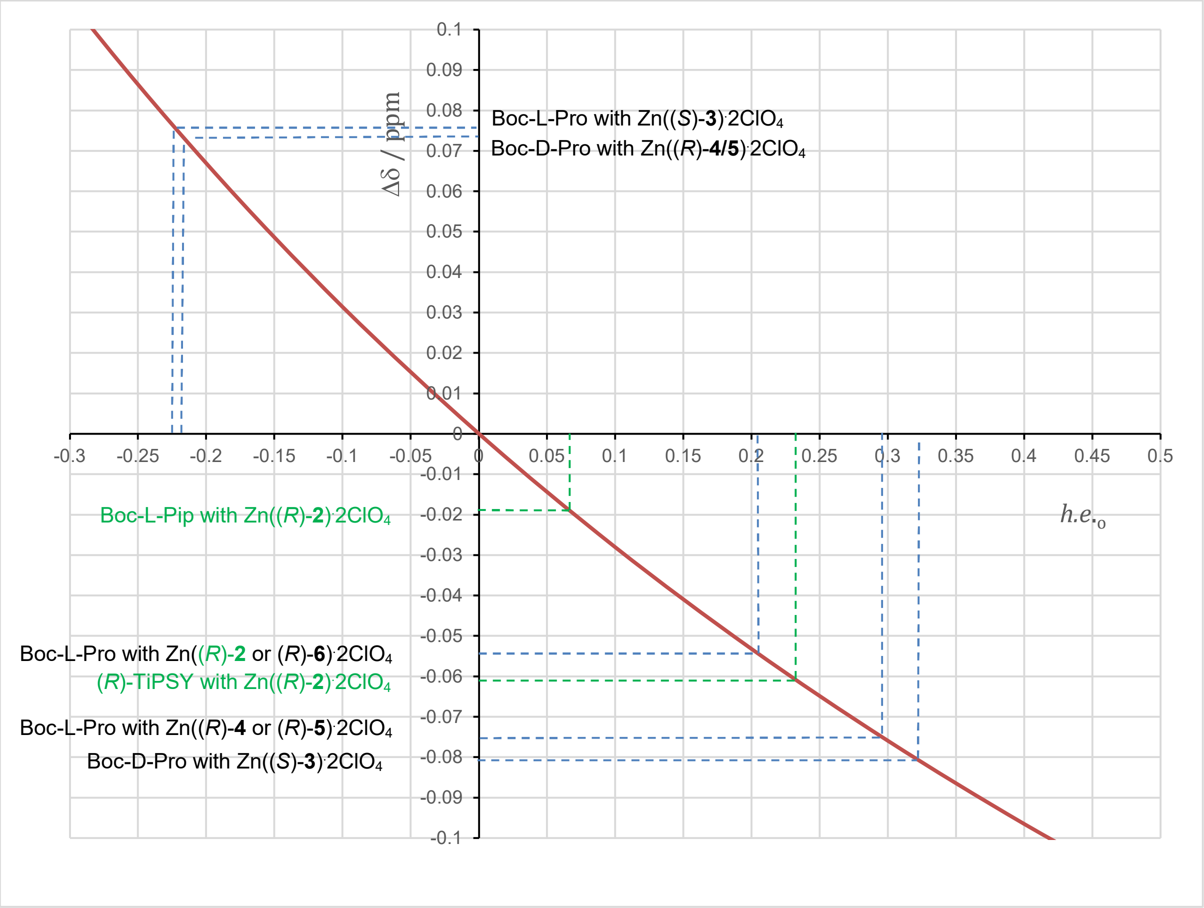

Figure S51 ^{19}F NMR chemical shifts in CD_3CN (ppm) for Aib tetramers N_3Aib((*R*)-TFEA) and Cbz(Xxx)Aib((*R*)-TFEA), Xxx = D-(αMeVal)$_2$, D-(αMeVal), L-Phe, Gly, D-Phe, L-(αMeVal), D-(αMeVal)$_2$. Chemical shifts are correlated with reported ability of each chiral N-terminal group to induce a local helical excess. Curve fits assume $\Delta G_R = -3.22$ kJ mol^{-1}.[S5]

6. NMR spectra of new compounds

6.1 Compound (*S*)-3

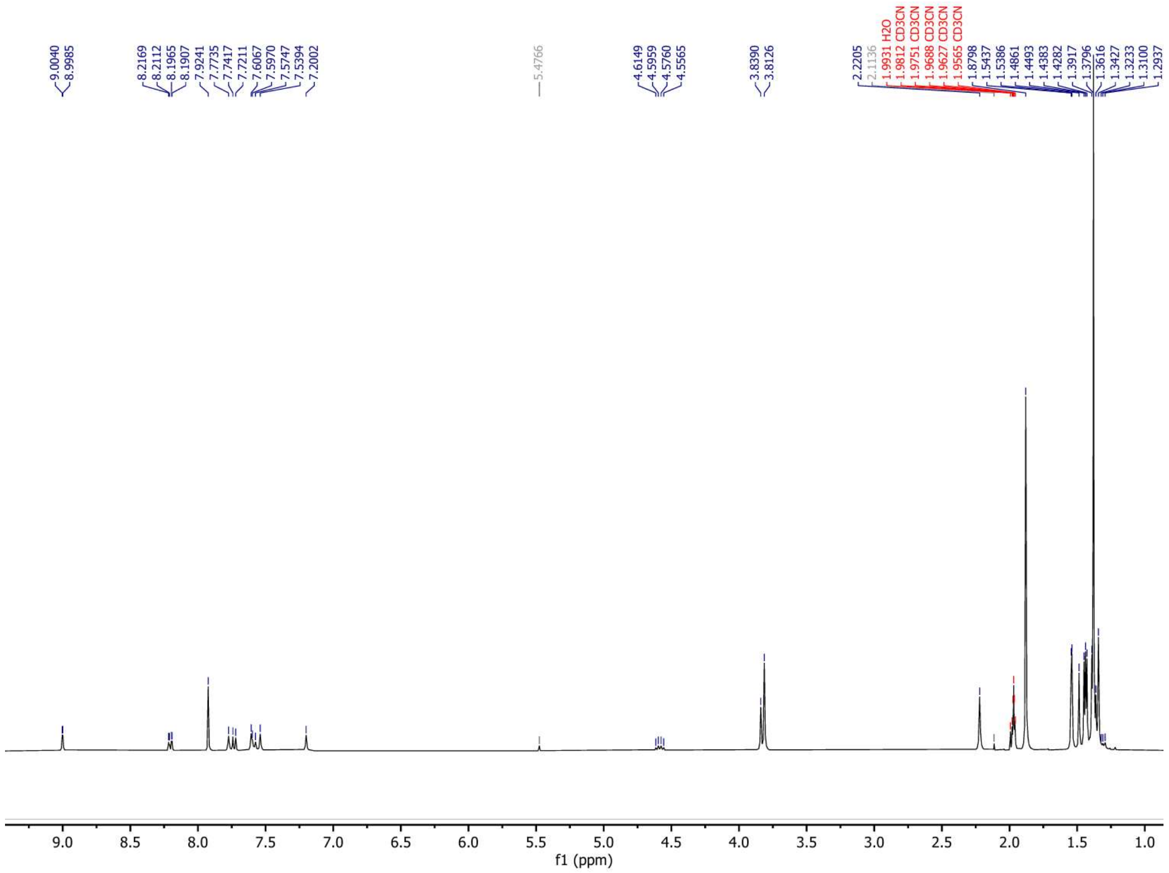

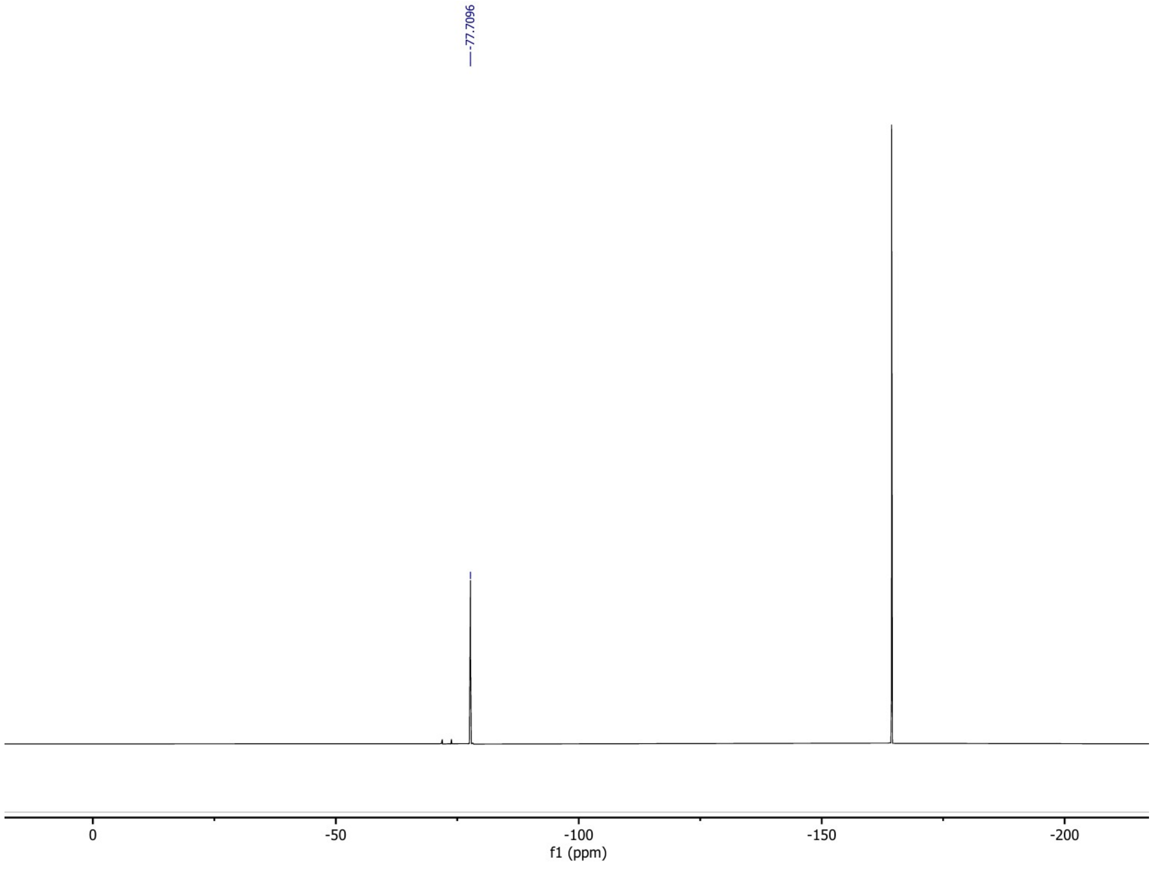

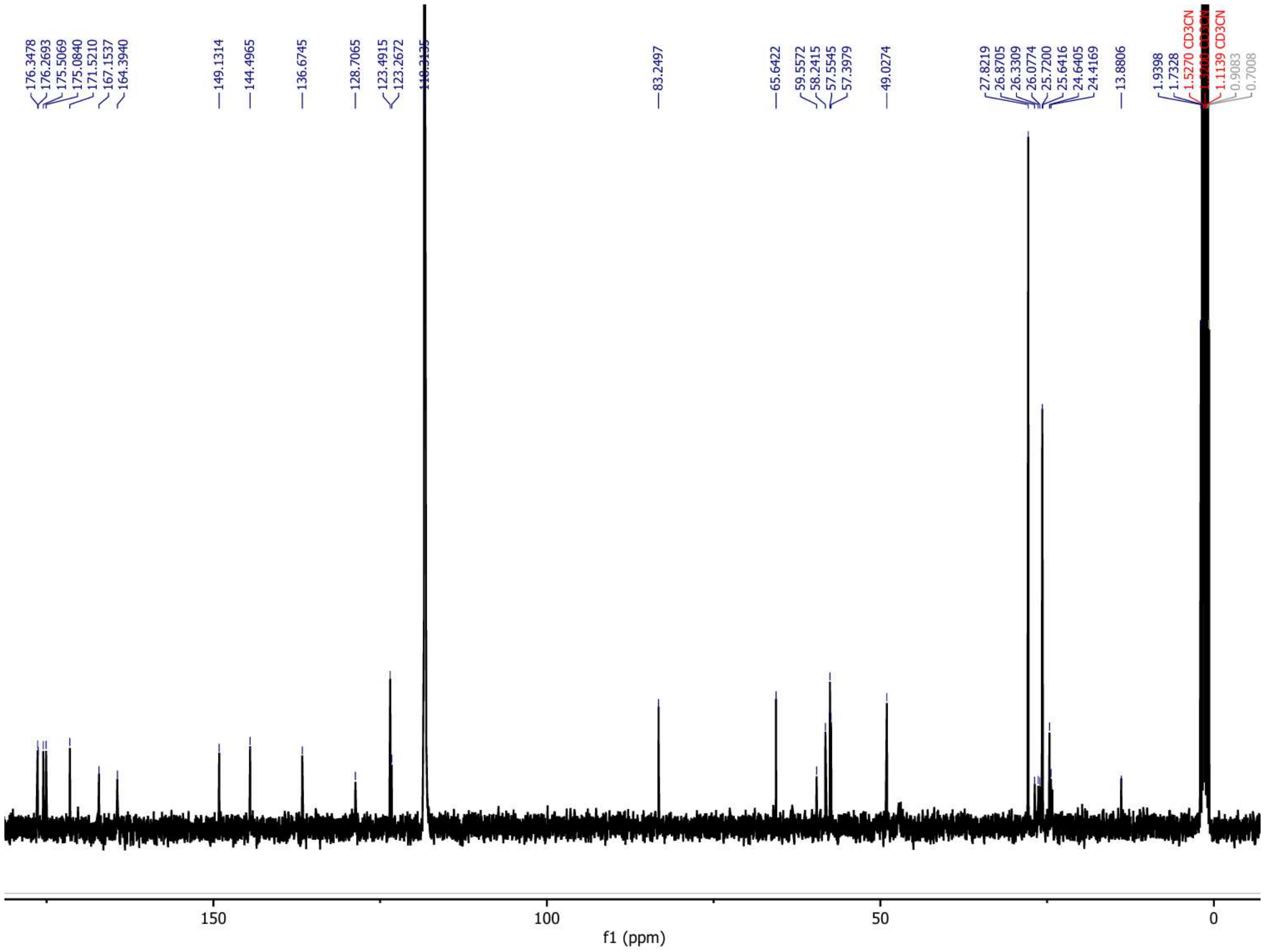
176.3478
176.2693
175.5069
175.0840
171.5210
167.1537
164.3940
149.1314
144.4965
136.6745
128.7065
123.4915
123.2672
83.2497
65.6422
59.5572
58.2415
57.5545
57.3979
49.0274
27.8219
26.8705
26.3309
26.0774
25.7200
25.6416
24.6405
24.4169
13.8806
1.9398
1.7328
1.5270 CD3CN
1.1139 CD3CN
0.9083
0.7008
150
100
50
0
f1 (ppm)

6.2 Compound (*R*)-4

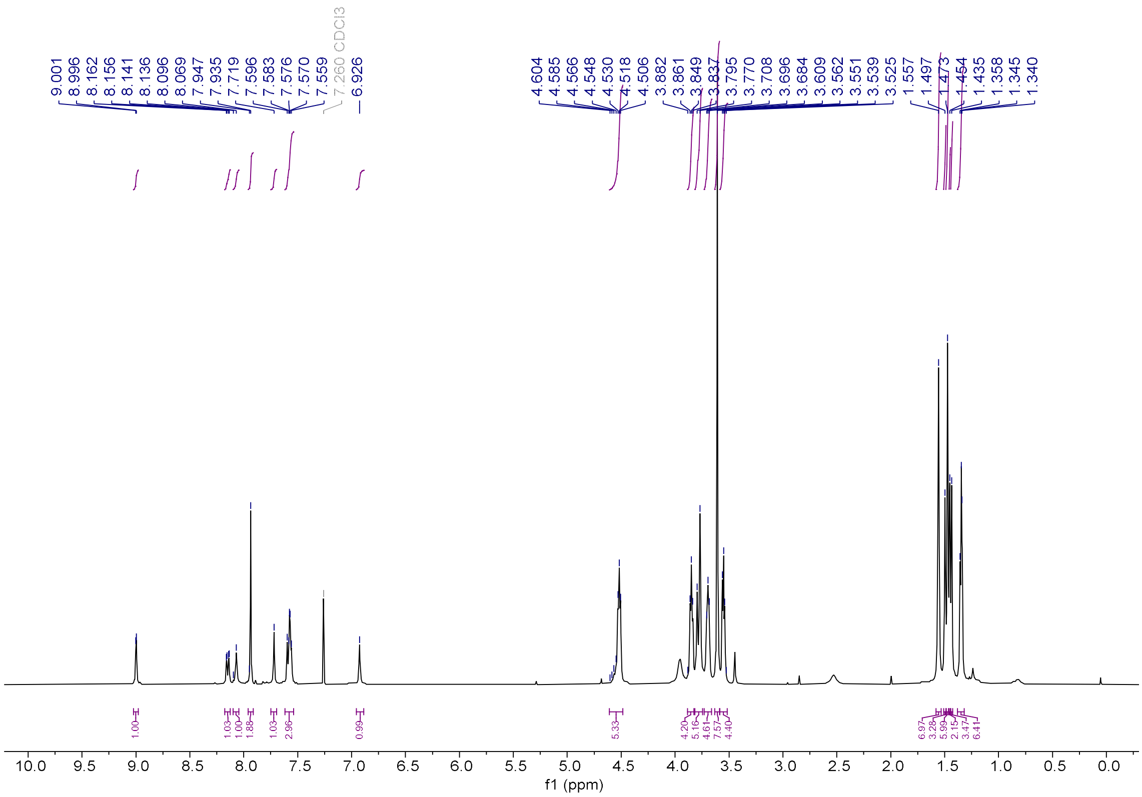

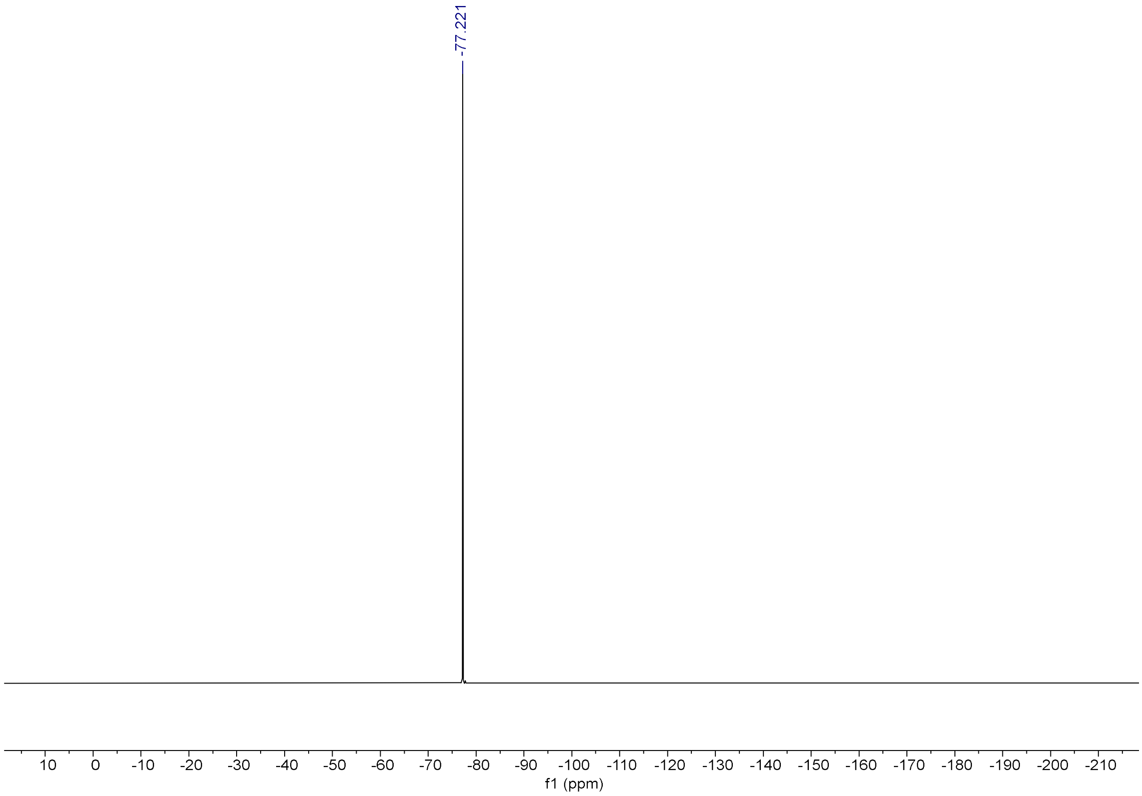

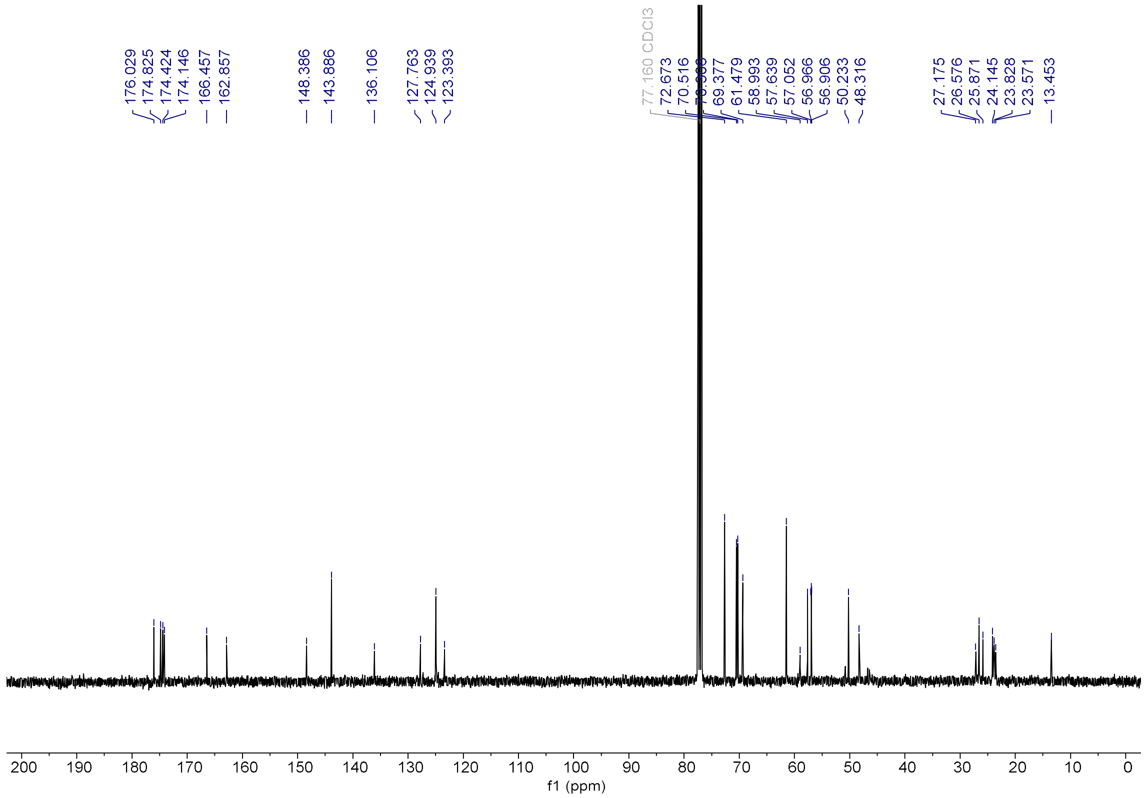
176.029
174.825
174.424
174.146
166.457
162.857
148.386
143.886
136.106
127.763
124.939
123.393
77.160 CDCl3
72.673
70.516
69.377
61.479
58.993
57.639
57.052
56.966
56.906
50.233
48.316
27.175
26.576
25.871
24.145
23.828
23.571
13.453
200 190 180 170 160 150 140 130 120 110 100 90 80 70 60 50 40 30 20 10 0
f1 (ppm)

6.3 Compound (*R*)-5

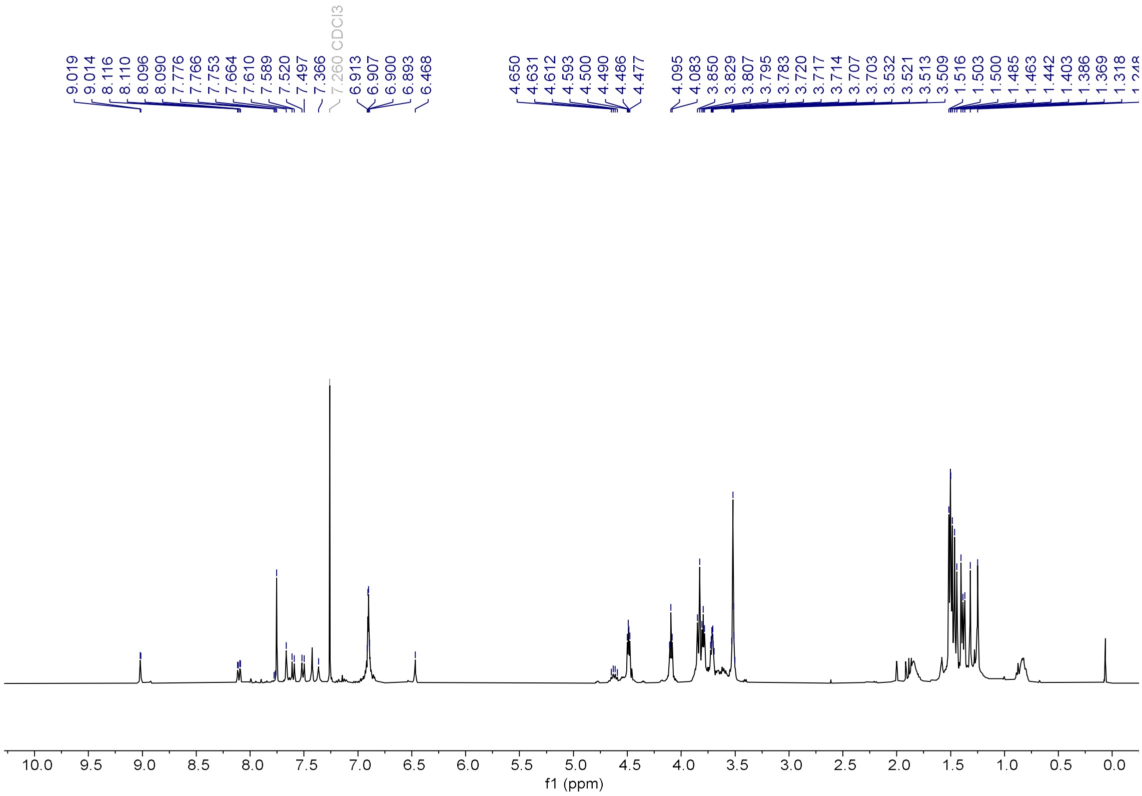

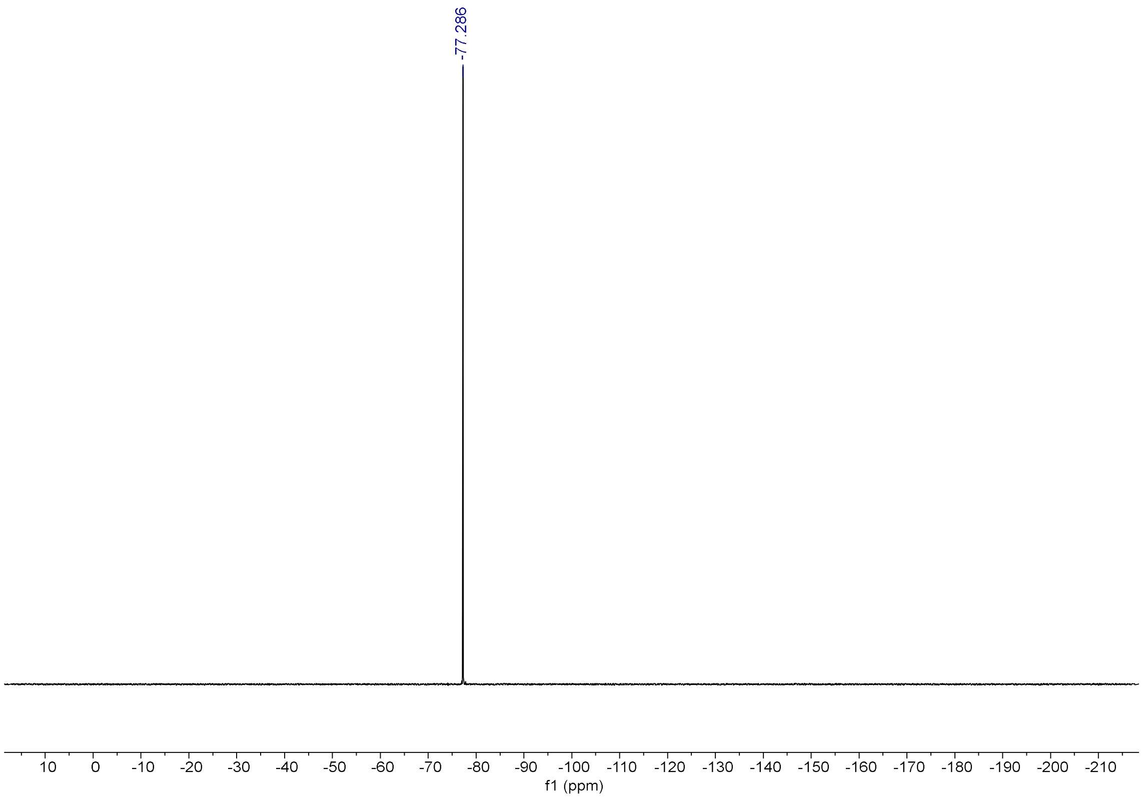

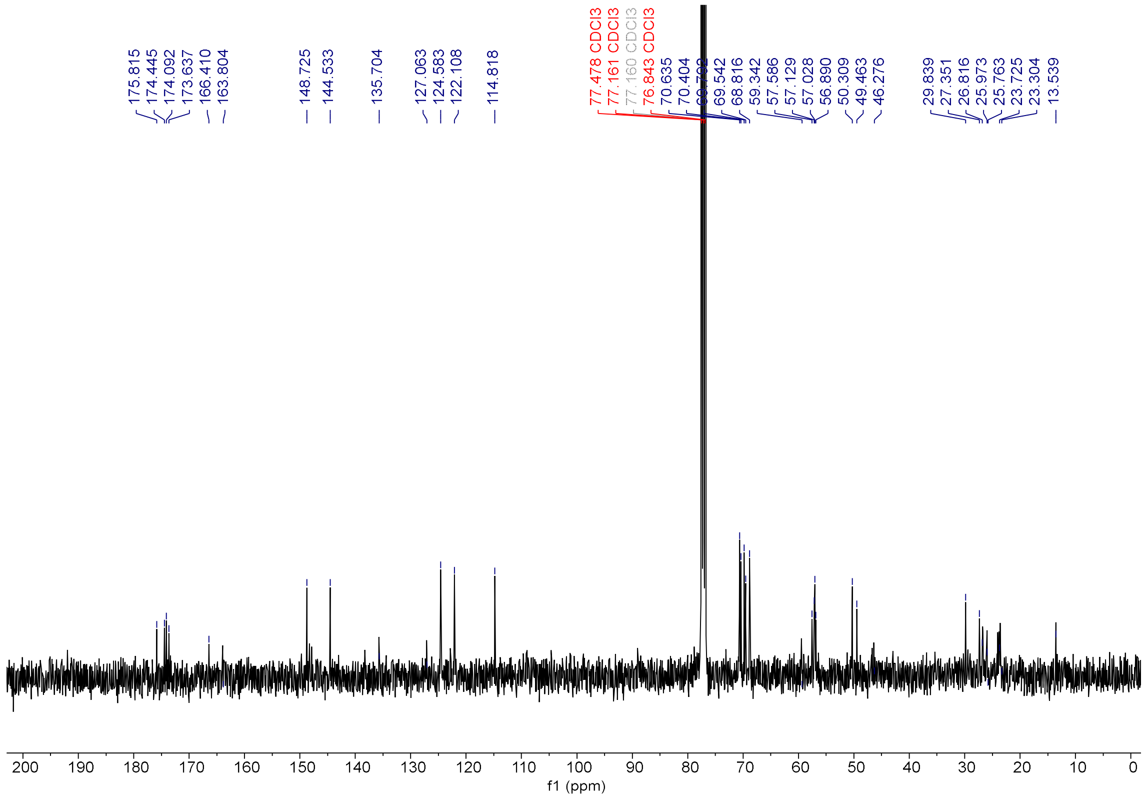

175.815
174.445
174.092
173.637
166.410
163.804
148.725
144.533
135.704
127.063
124.583
122.108
114.818
77.478 CDCl3
77.161 CDCl3
77.160 CDCl3
76.843 CDCl3
70.635
70.404
69.542
68.816
59.342
57.586
57.129
57.028
56.890
50.309
49.463
46.276
29.839
27.351
26.816
25.973
25.763
23.725
23.304
13.539
f1 (ppm)

6.4 Compound (*R*)-6

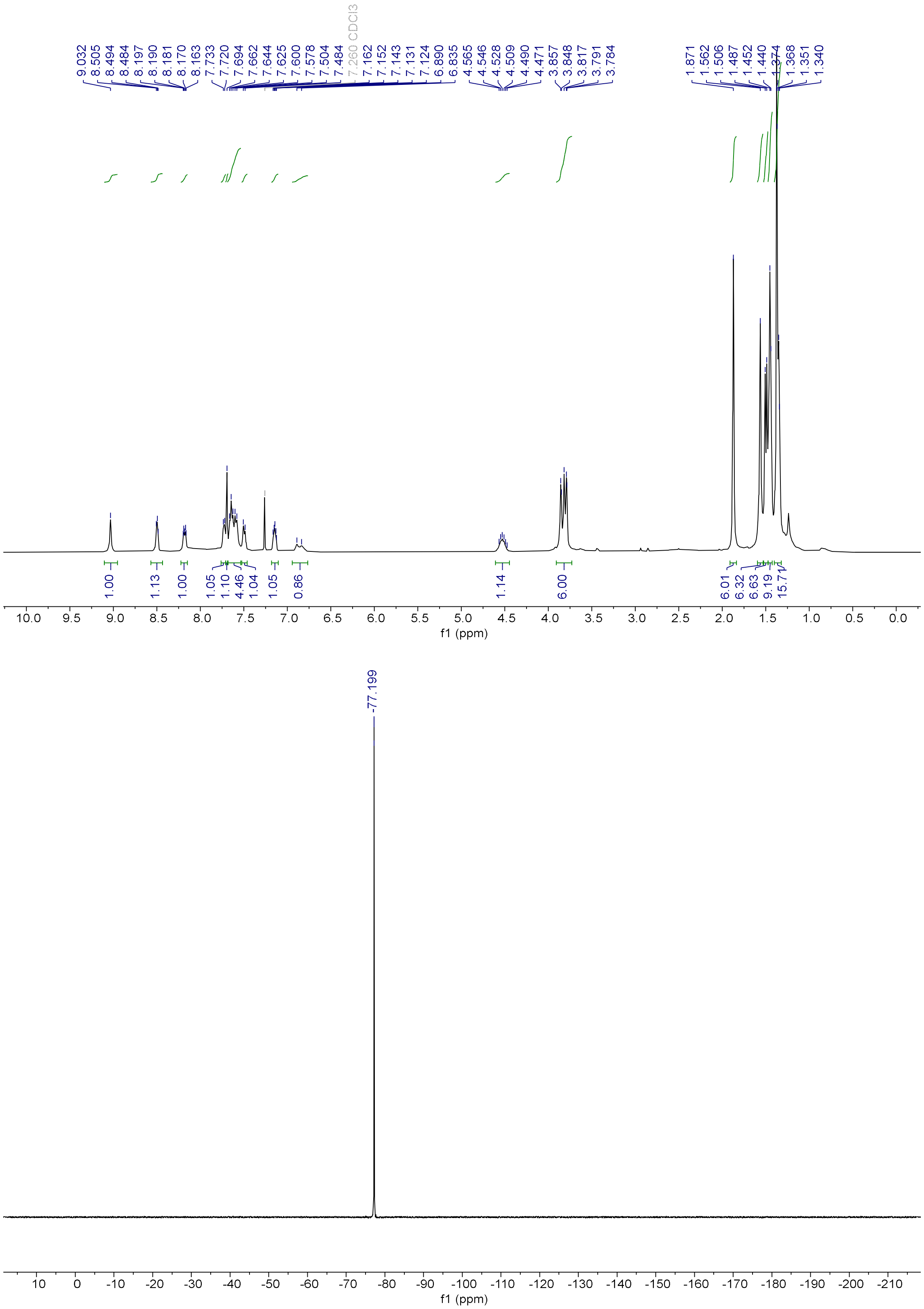

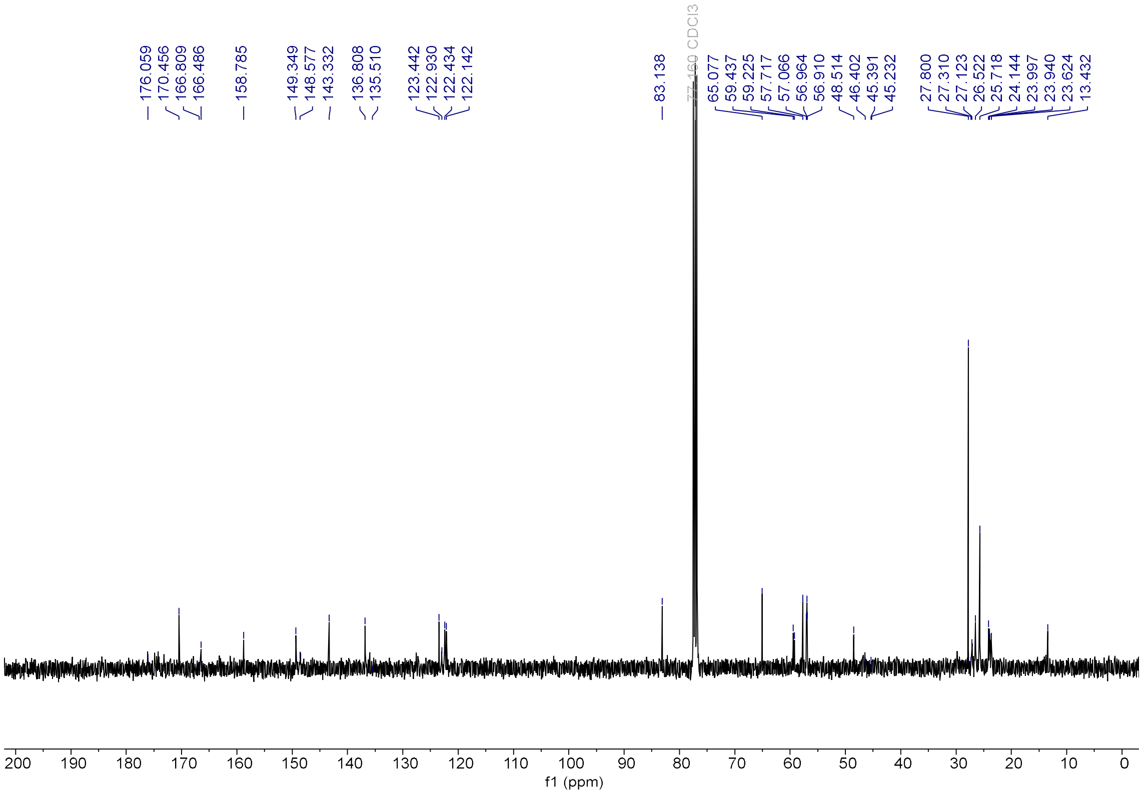
176.059
170.456
166.809
166.486
158.785
149.349
148.577
143.332
136.808
135.510
123.442
122.930
122.434
122.142
83.138
77.160 CDCl3
65.077
59.437
59.225
57.717
57.066
56.964
56.910
48.514
46.402
45.391
45.232
27.800
27.310
27.123
26.522
25.718
24.144
23.997
23.940
23.624
13.432
200 190 180 170 160 150 140 130 120 110 100 90 80 70 60 50 40 30 20 10 0
f1 (ppm)

6.5 Compound S13

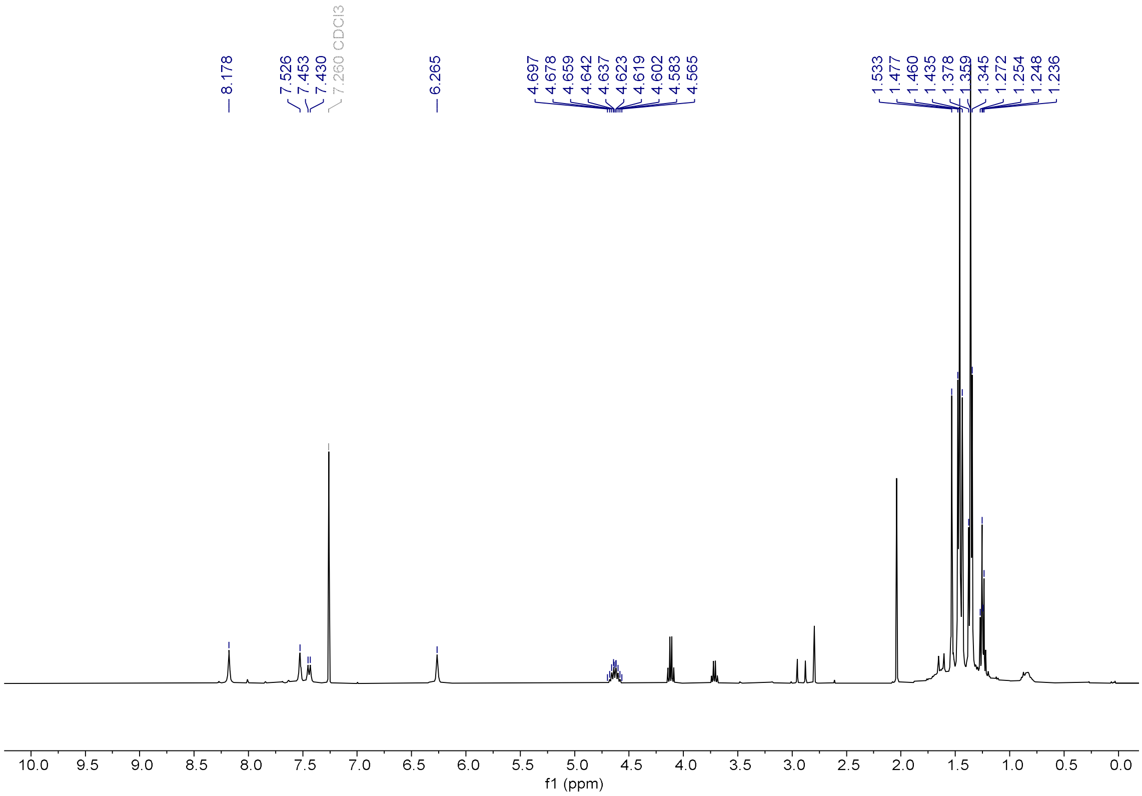

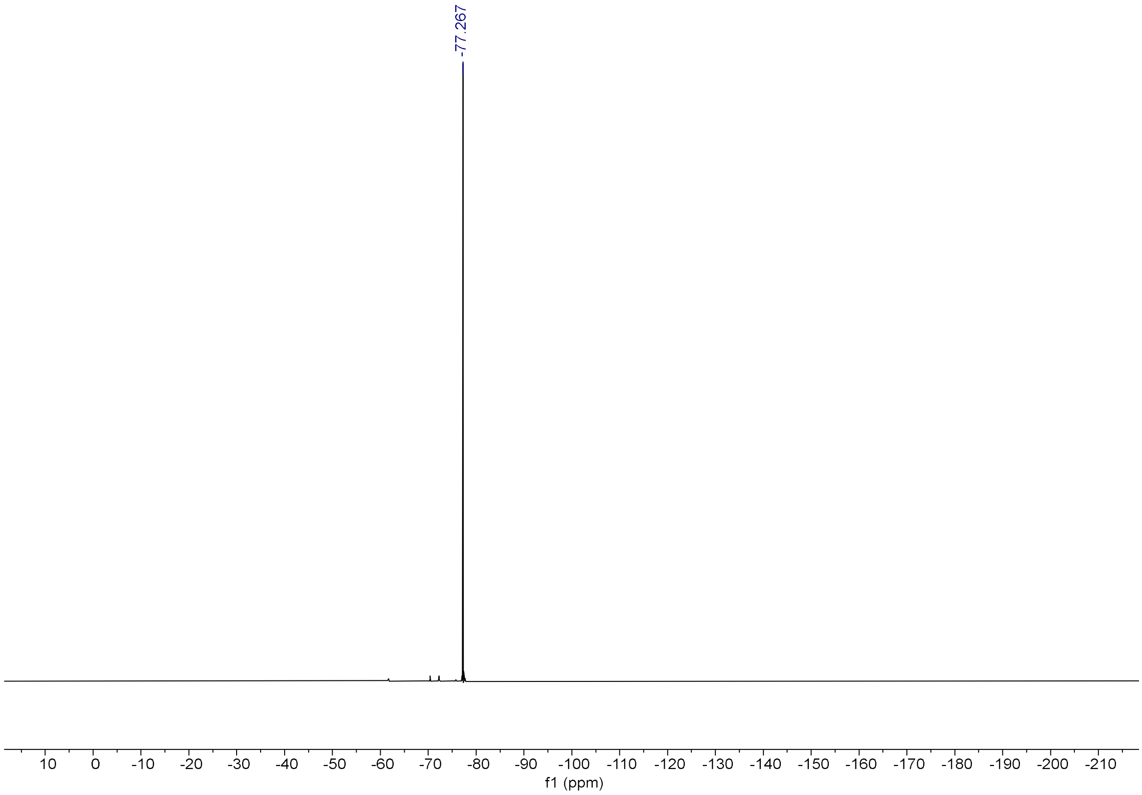

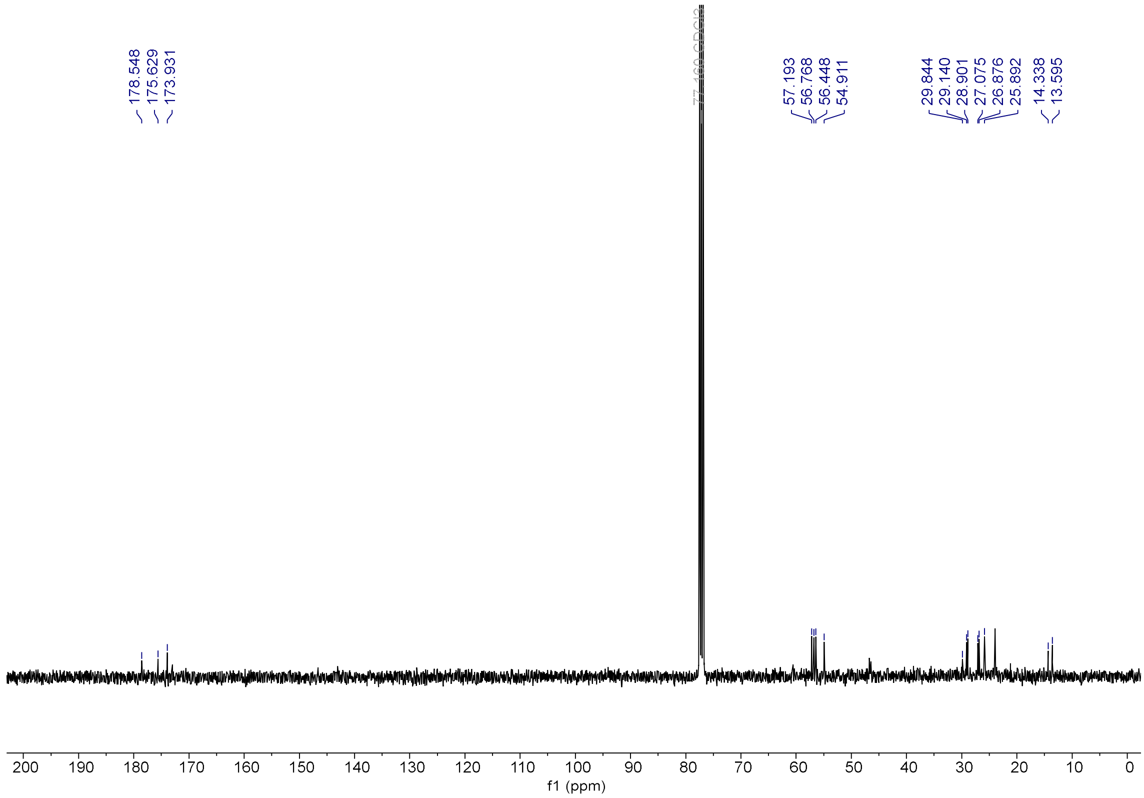
178.548
175.629
173.931
77.160 CDCl3
57.193
56.768
56.448
54.911
29.844
29.140
28.901
27.075
26.876
25.892
14.338
13.595
200 190 180 170 160 150 140 130 120 110 100 90 80 70 60 50 40 30 20 10 0
f1 (ppm)

6.6 Compound S16

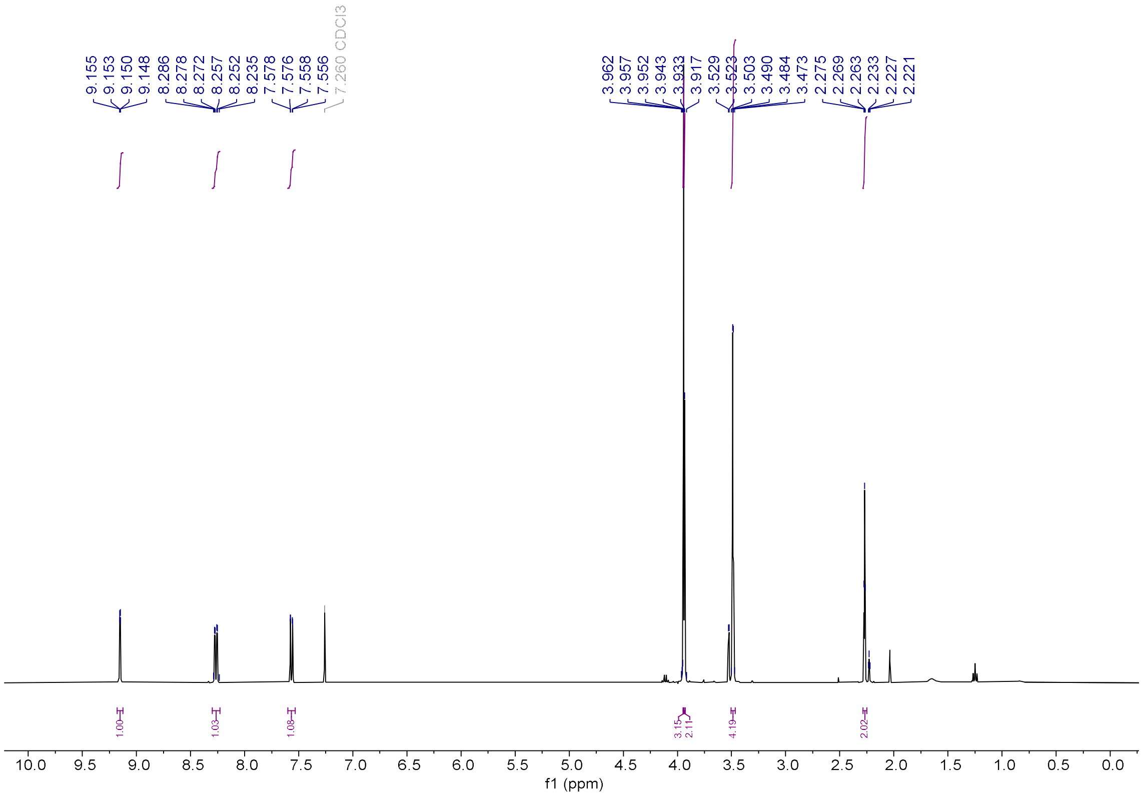

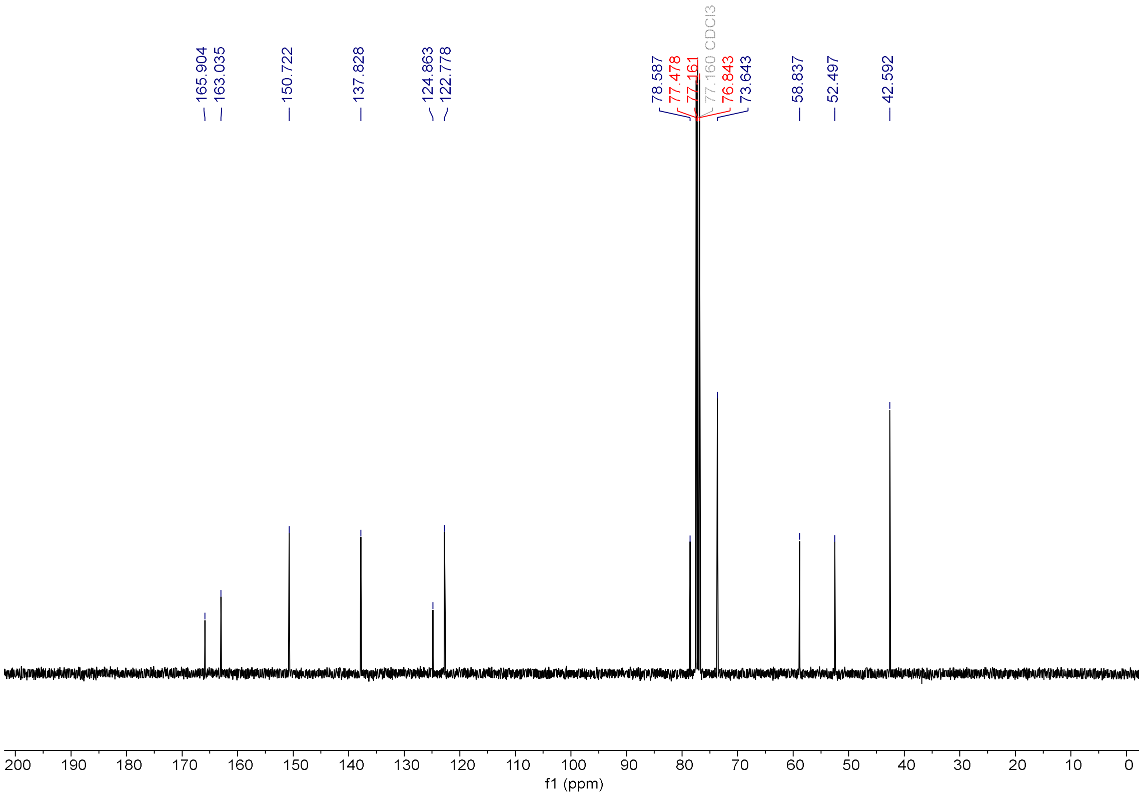

6.7 Compound S17

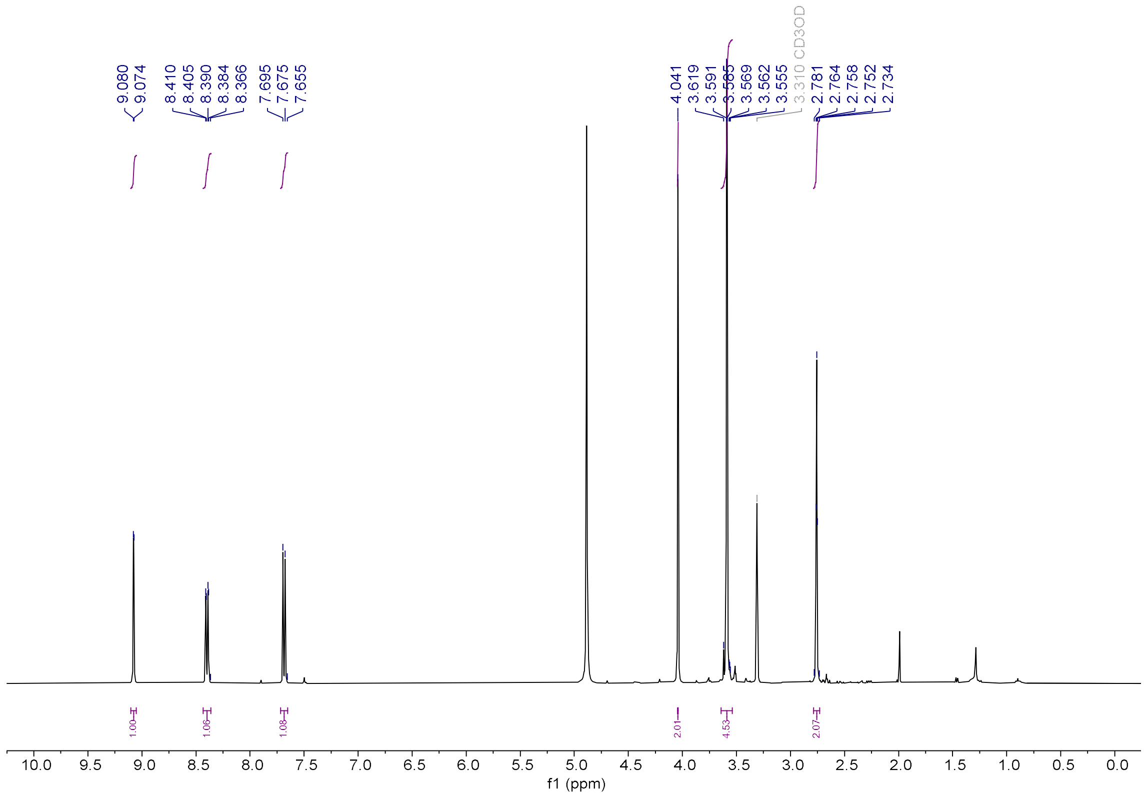

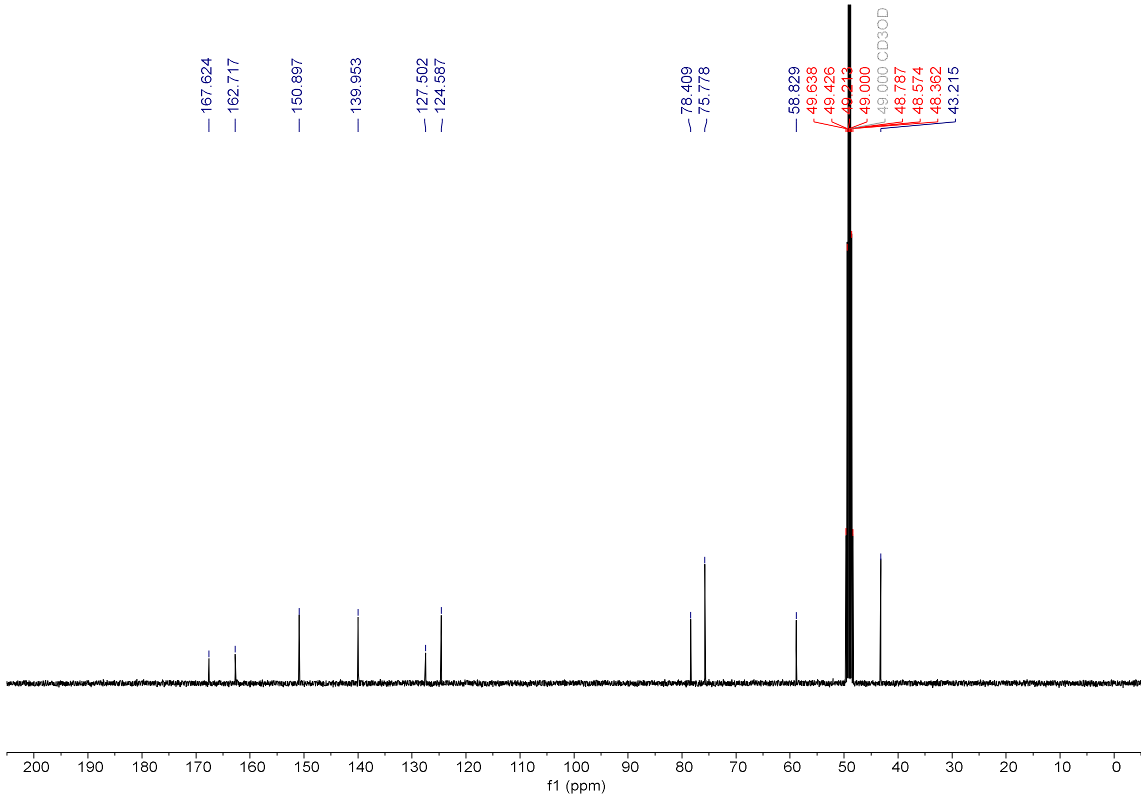

6.8 Compound (*R*)-7

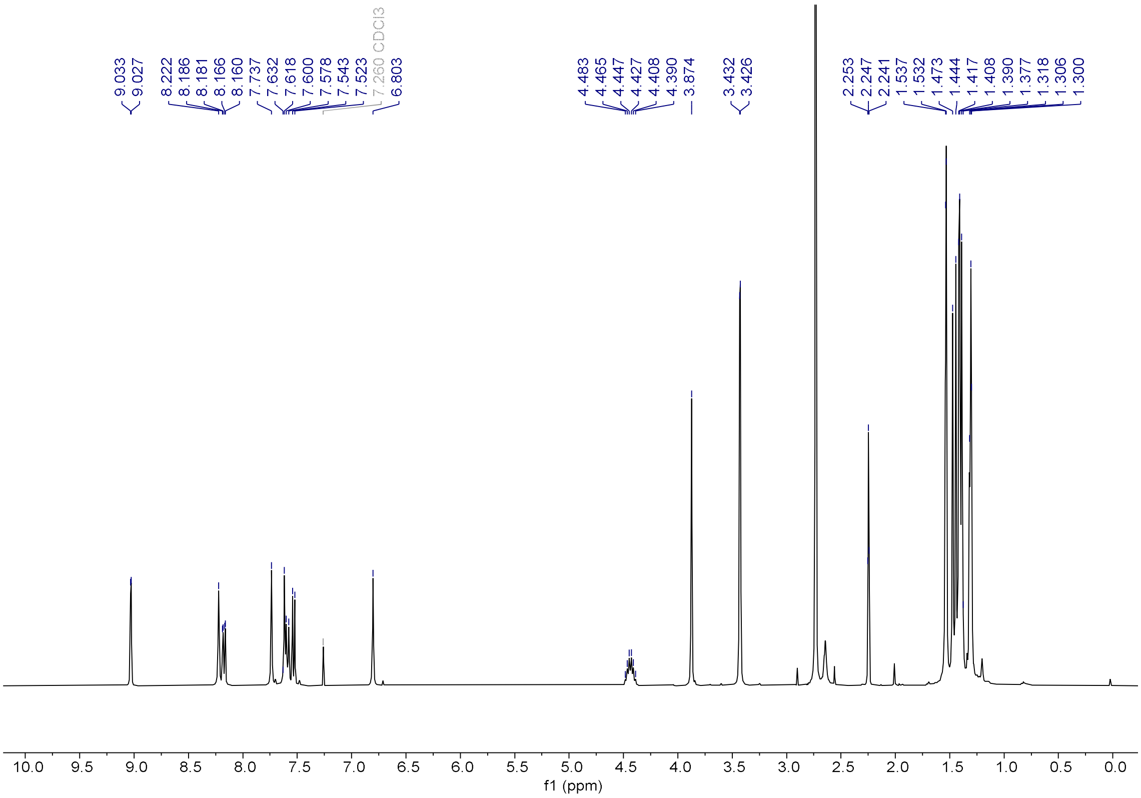

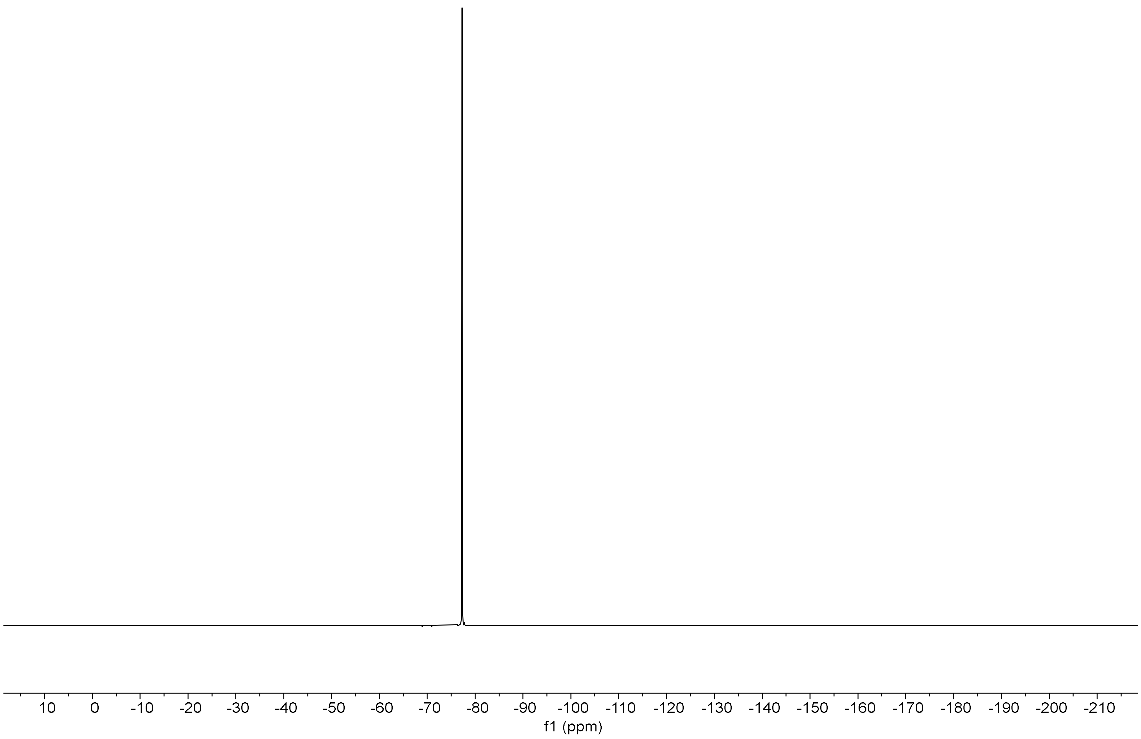

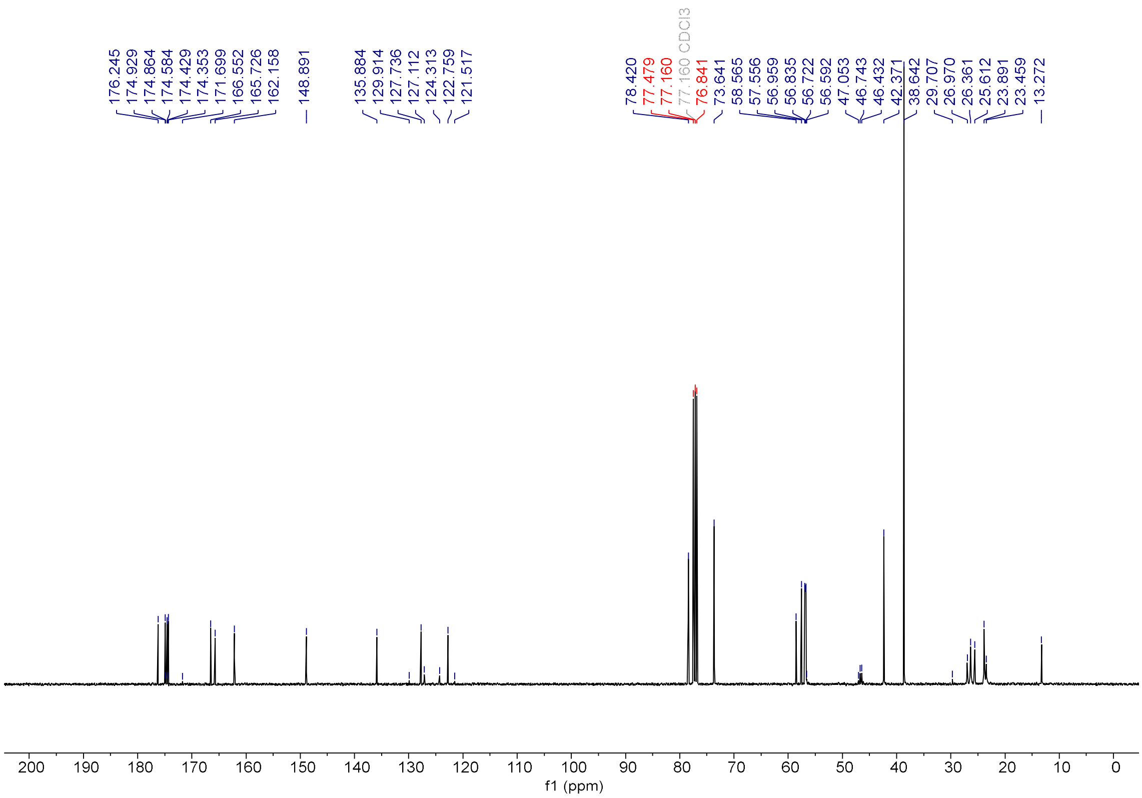
176.245
174.929
174.864
174.584
174.429
174.353
171.699
166.552
165.726
162.158
148.891
135.884
129.914
127.736
127.112
124.313
122.759
121.517
78.420
77.479
77.160
77.160 CDCl3
76.841
73.641
58.565
57.556
56.959
56.835
56.722
56.592
47.053
46.743
46.432
42.371
38.642
29.707
26.970
26.361
25.612
23.891
23.459
13.272
f1 (ppm)

6.9 Compound S18

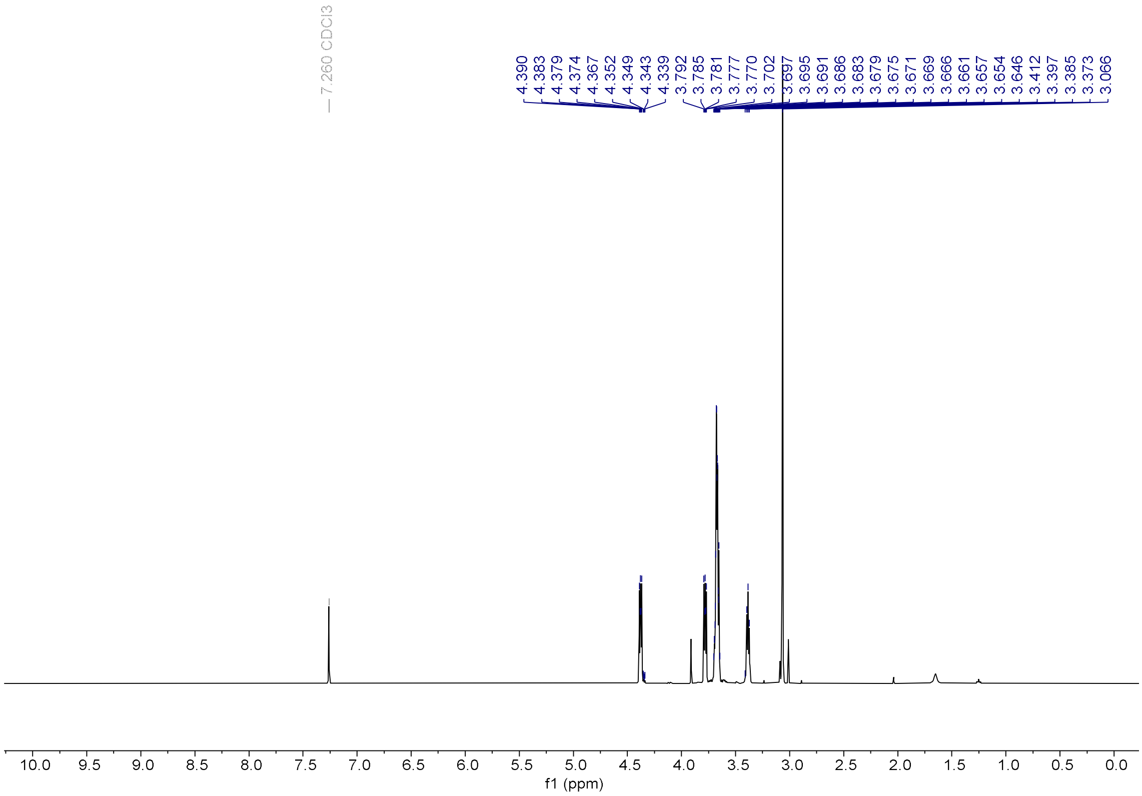

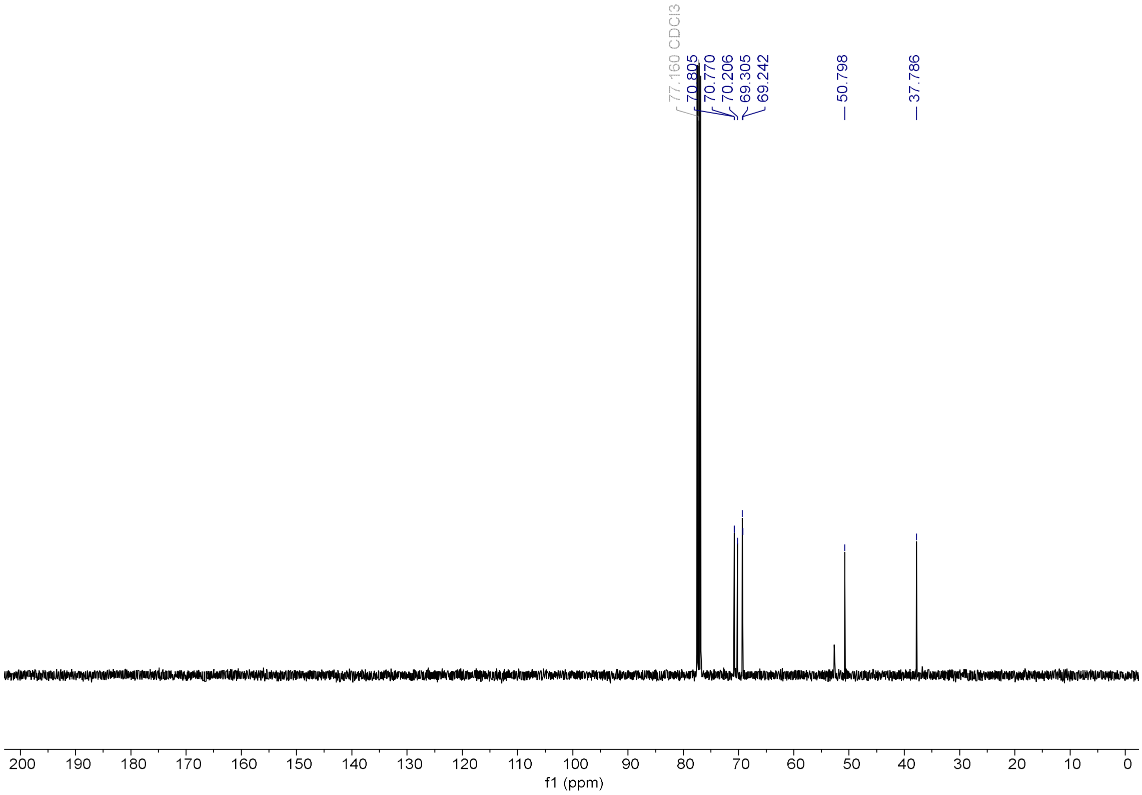

6.10 Compound (*R*)-8

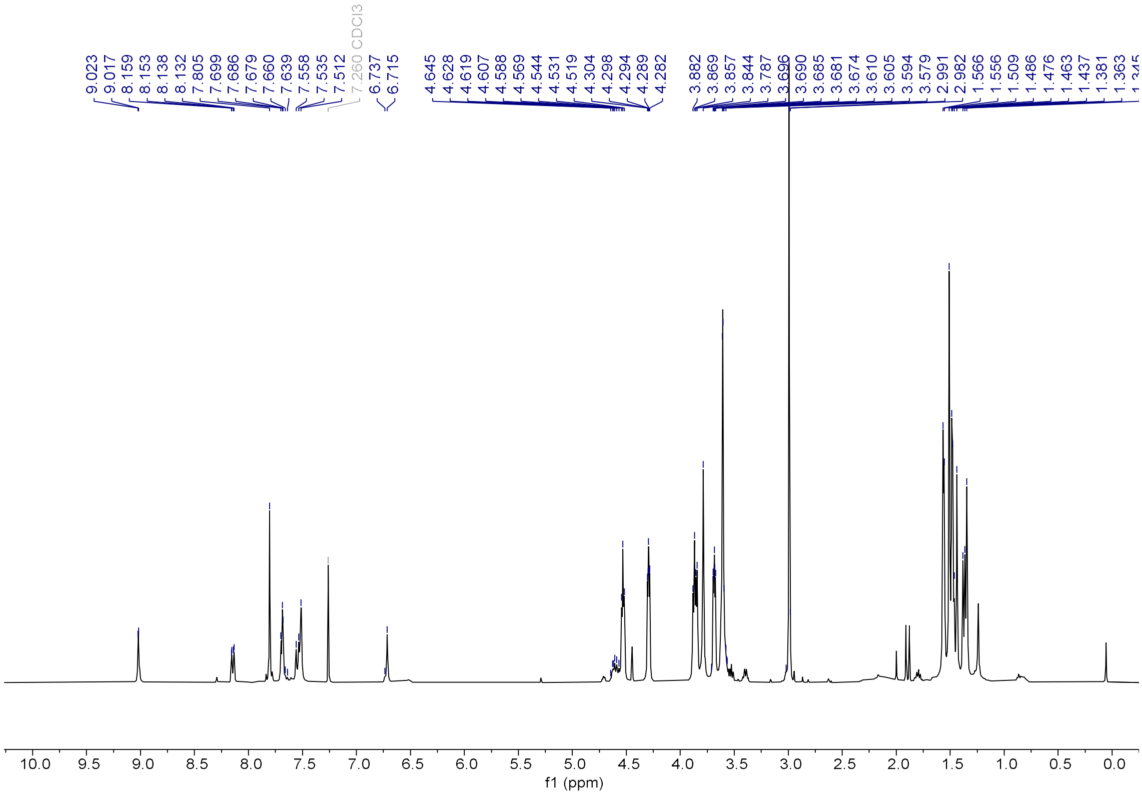

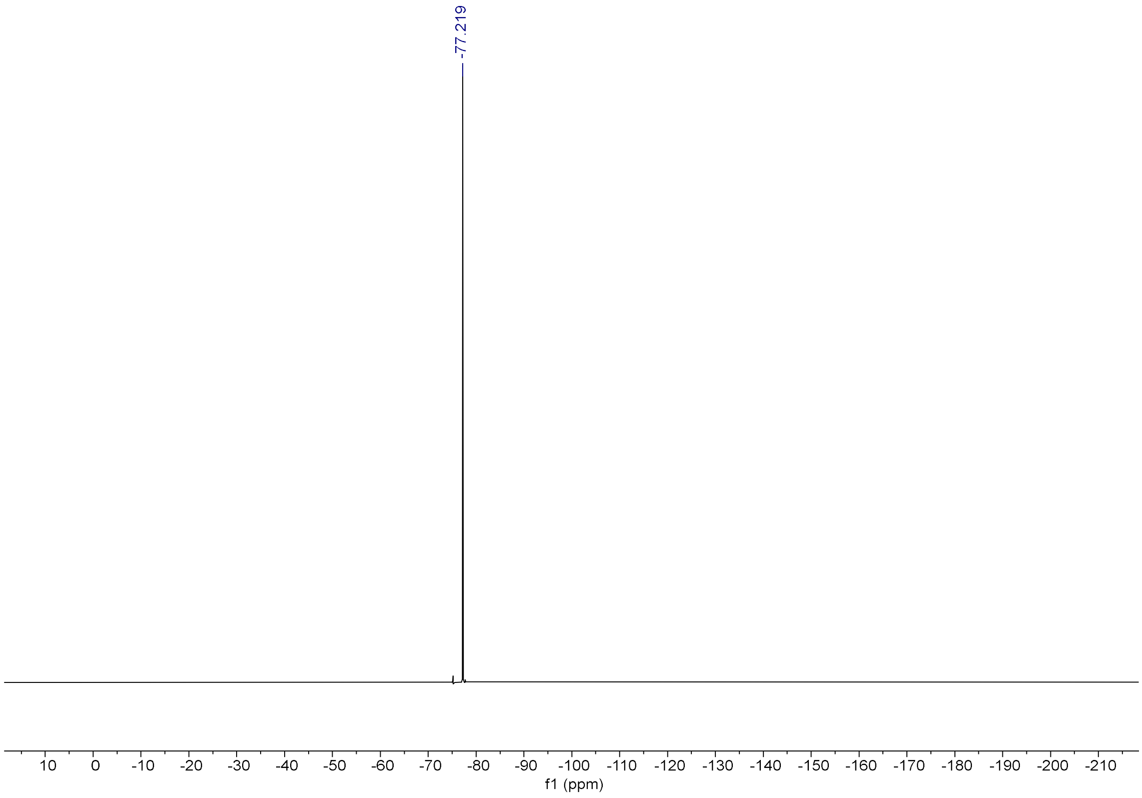

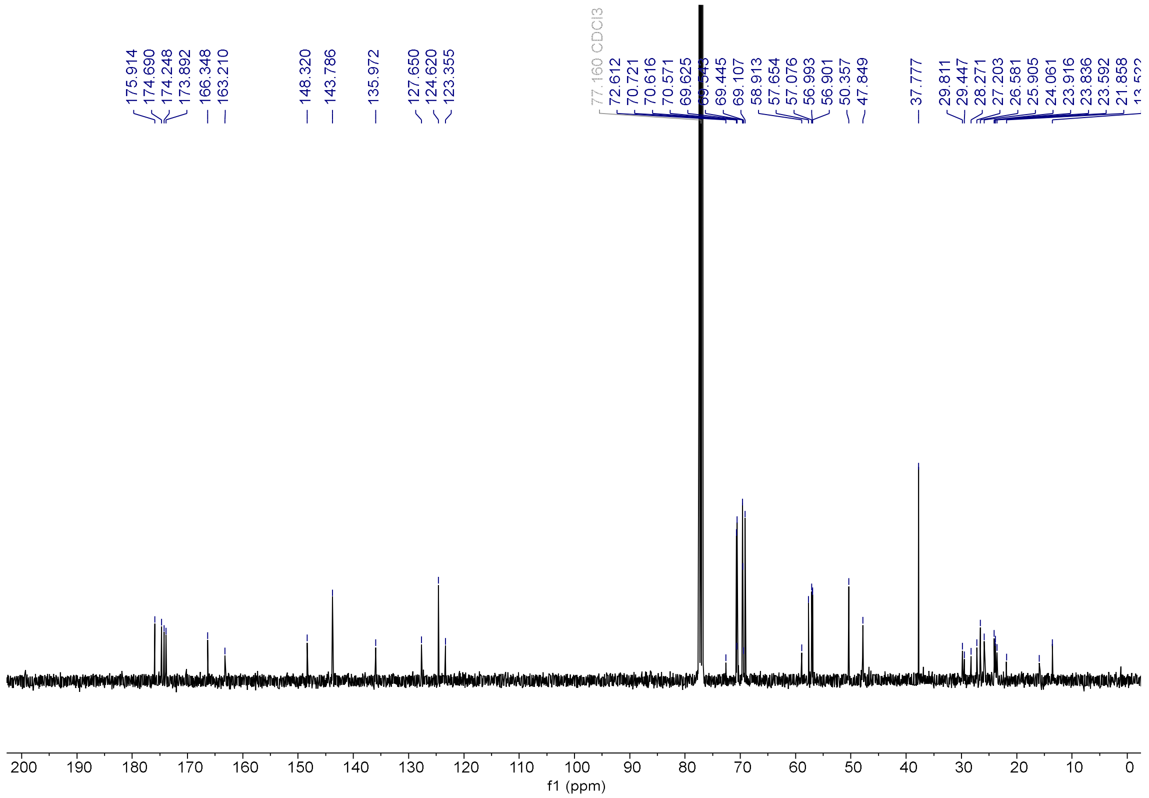

6.11 Compound 10

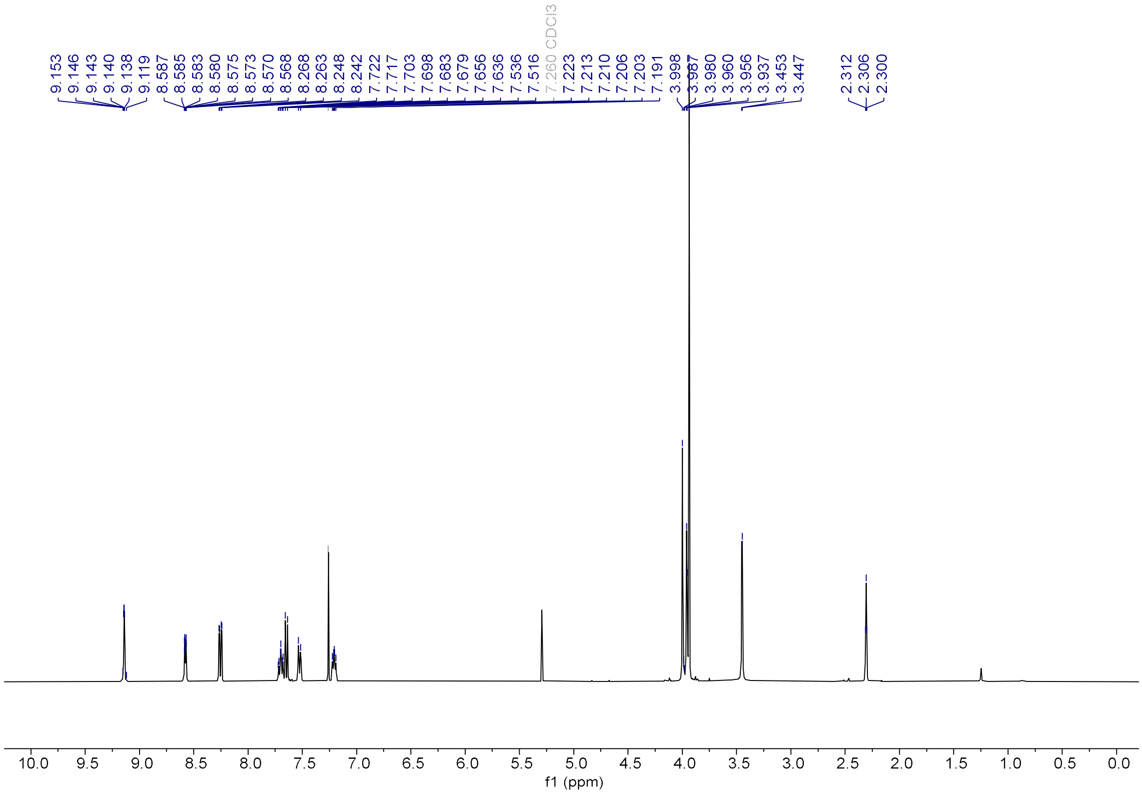

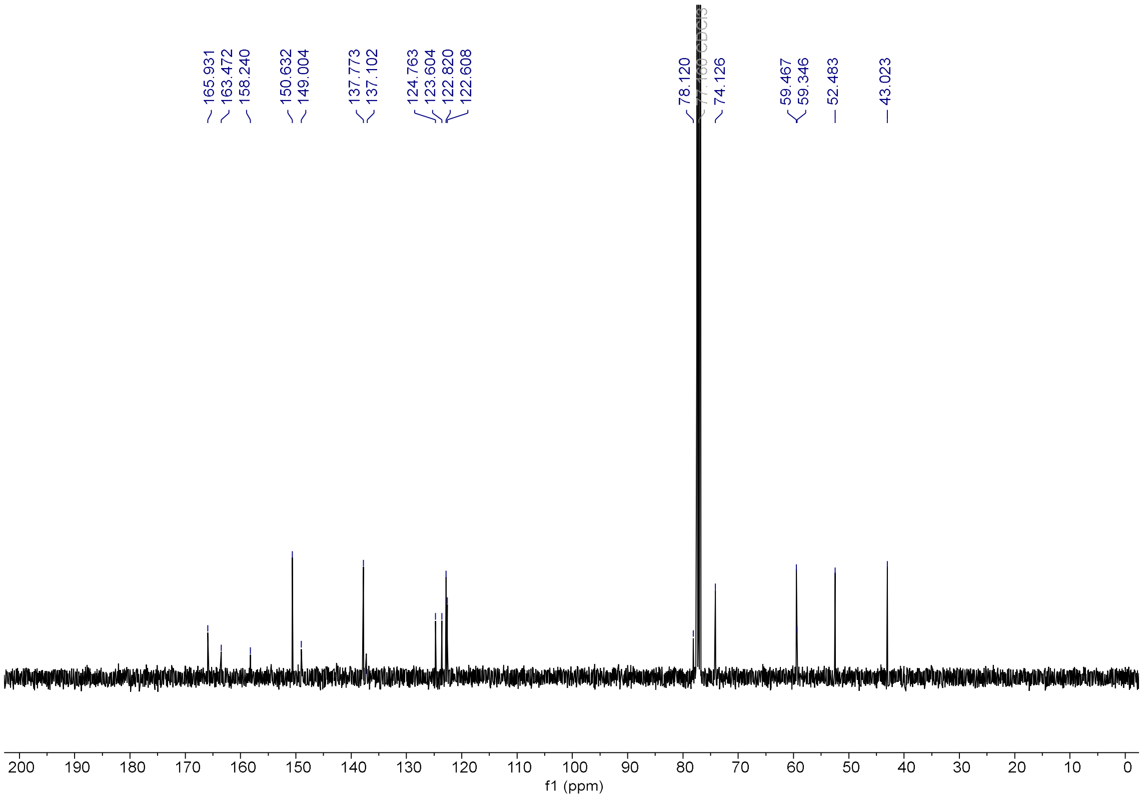

6.12 Compound S19

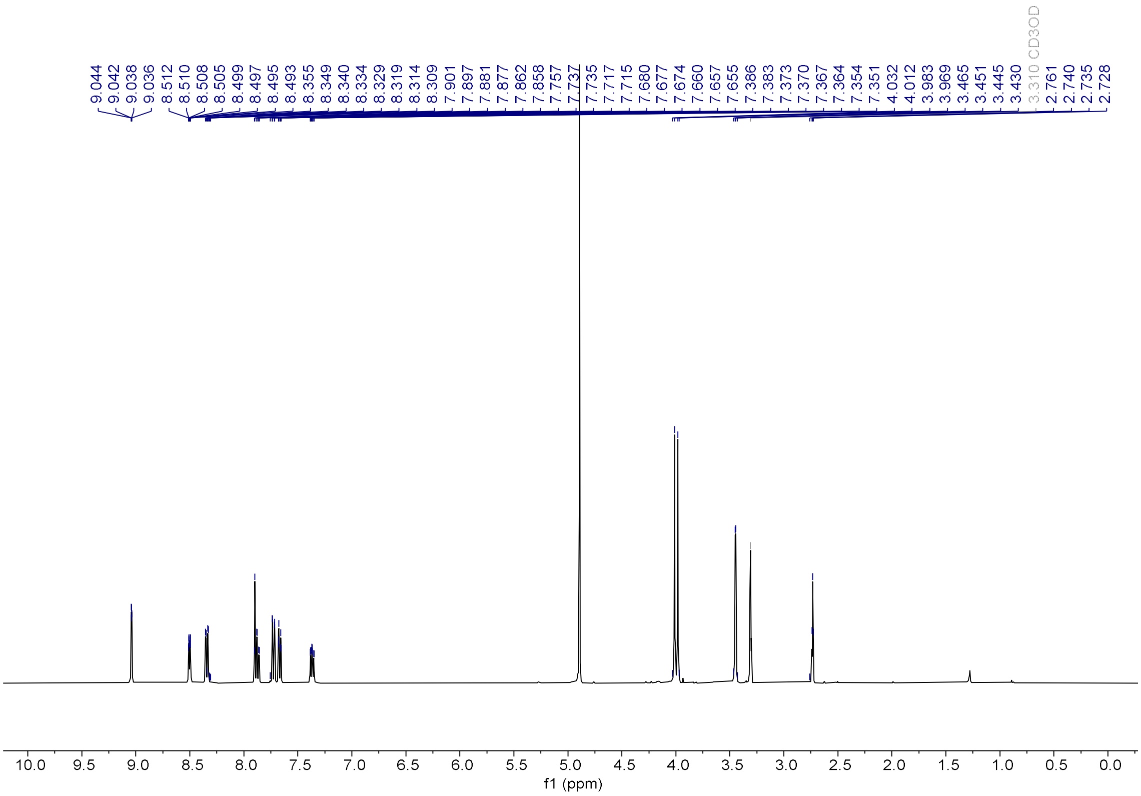

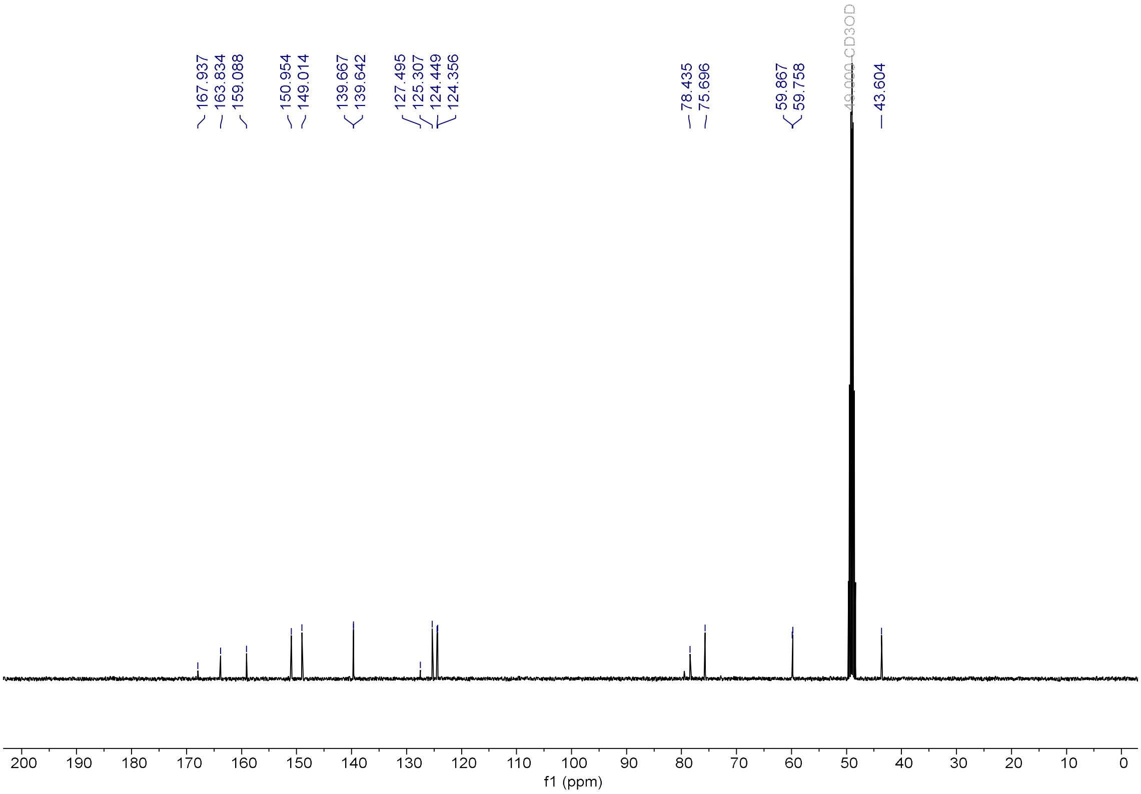

6.13 Compound (*R*)-11

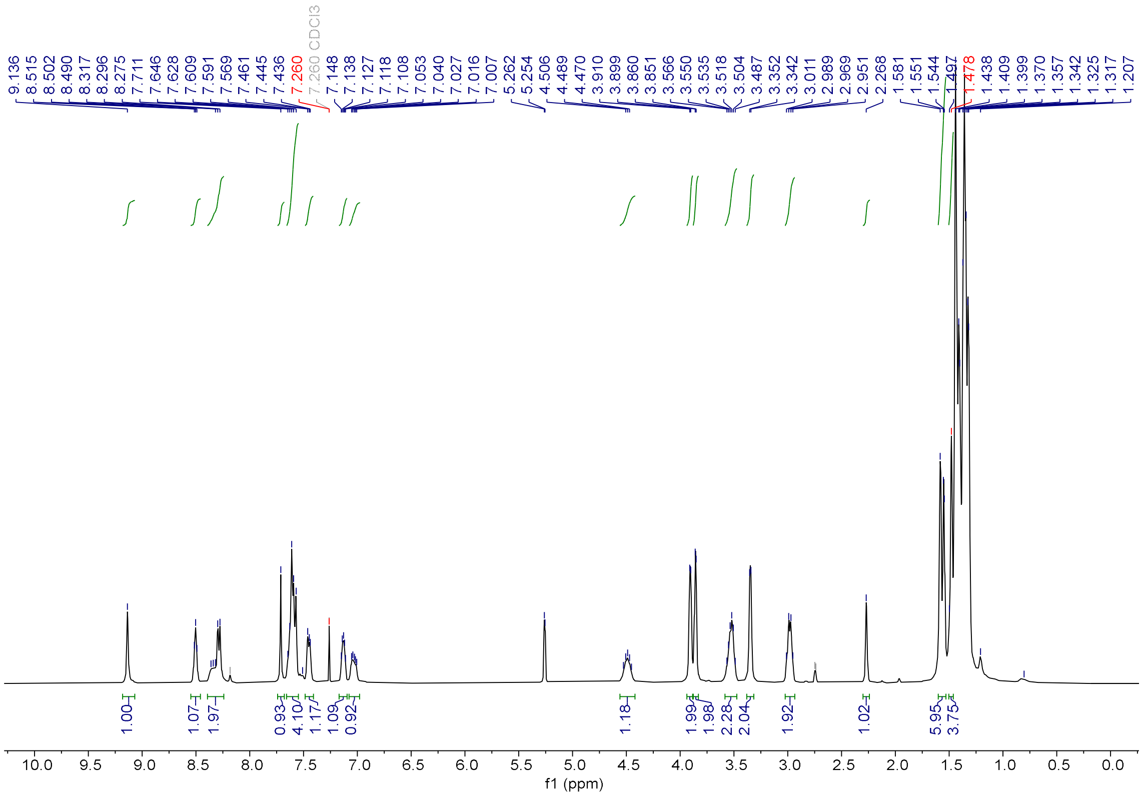

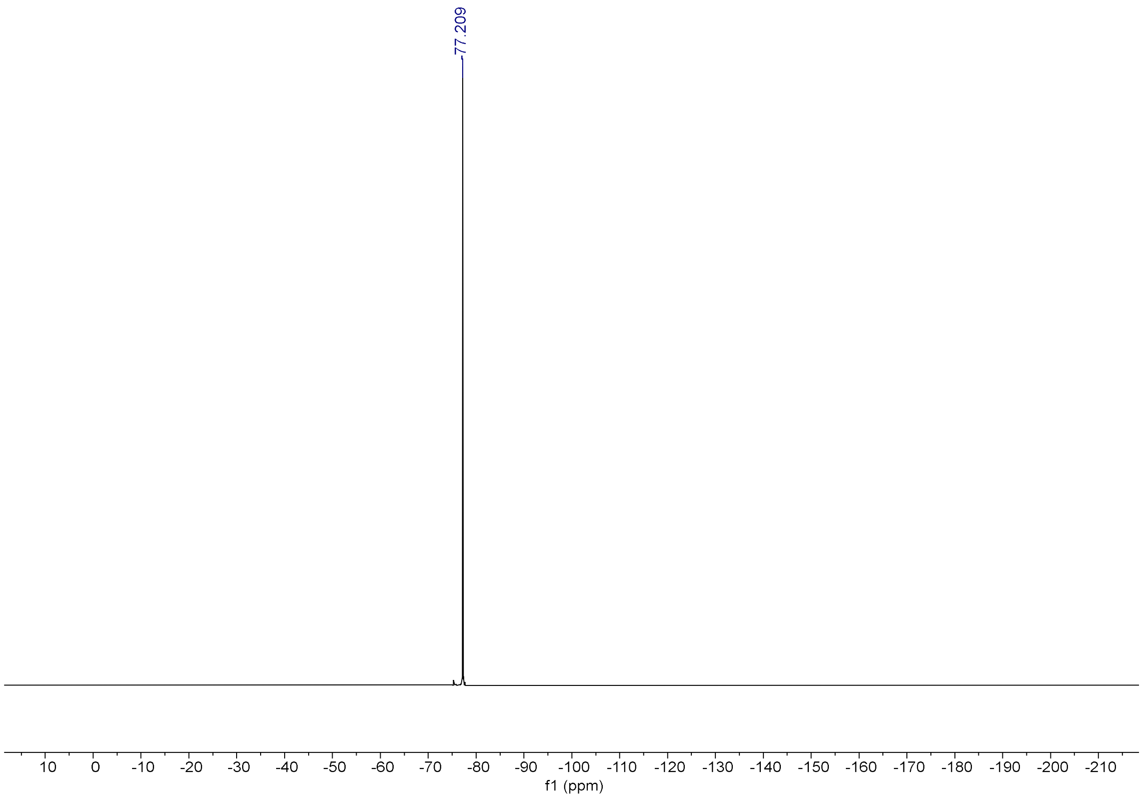

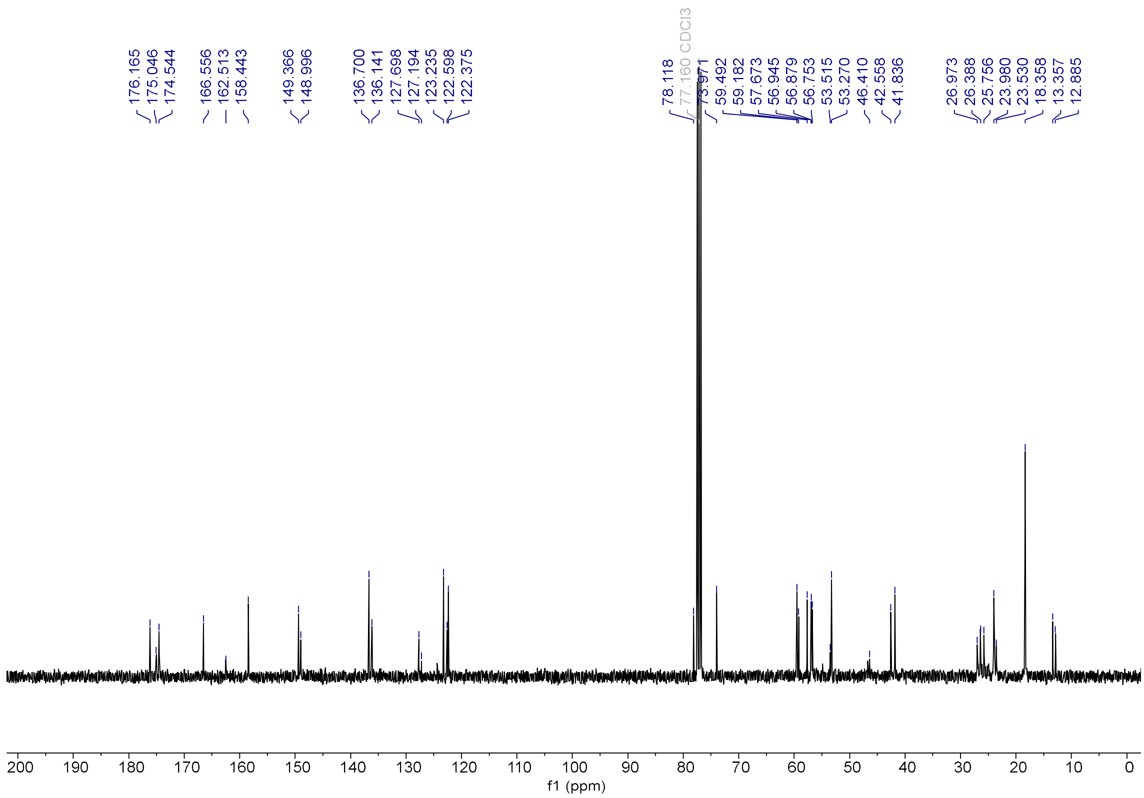
176.165
175.046
174.544
166.556
162.513
158.443
149.366
148.996
136.700
136.141
127.698
127.194
123.235
122.598
122.375
78.118
77.160 CDCl3
73.971
59.492
59.182
57.673
56.945
56.879
56.753
53.515
53.270
46.410
42.558
41.836
26.973
26.388
25.756
23.980
23.530
18.358
13.357
12.885
200 190 180 170 160 150 140 130 120 110 100 90 80 70 60 50 40 30 20 10 0
f1 (ppm)

6.14 Compound S20

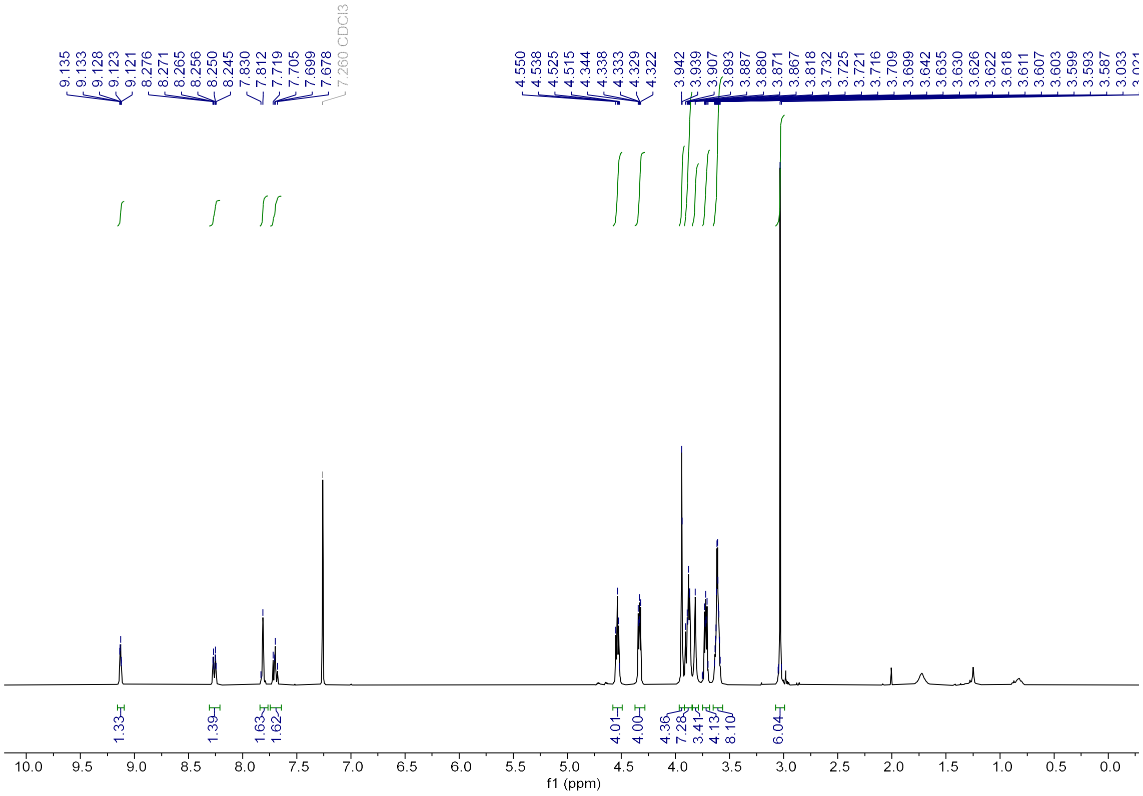

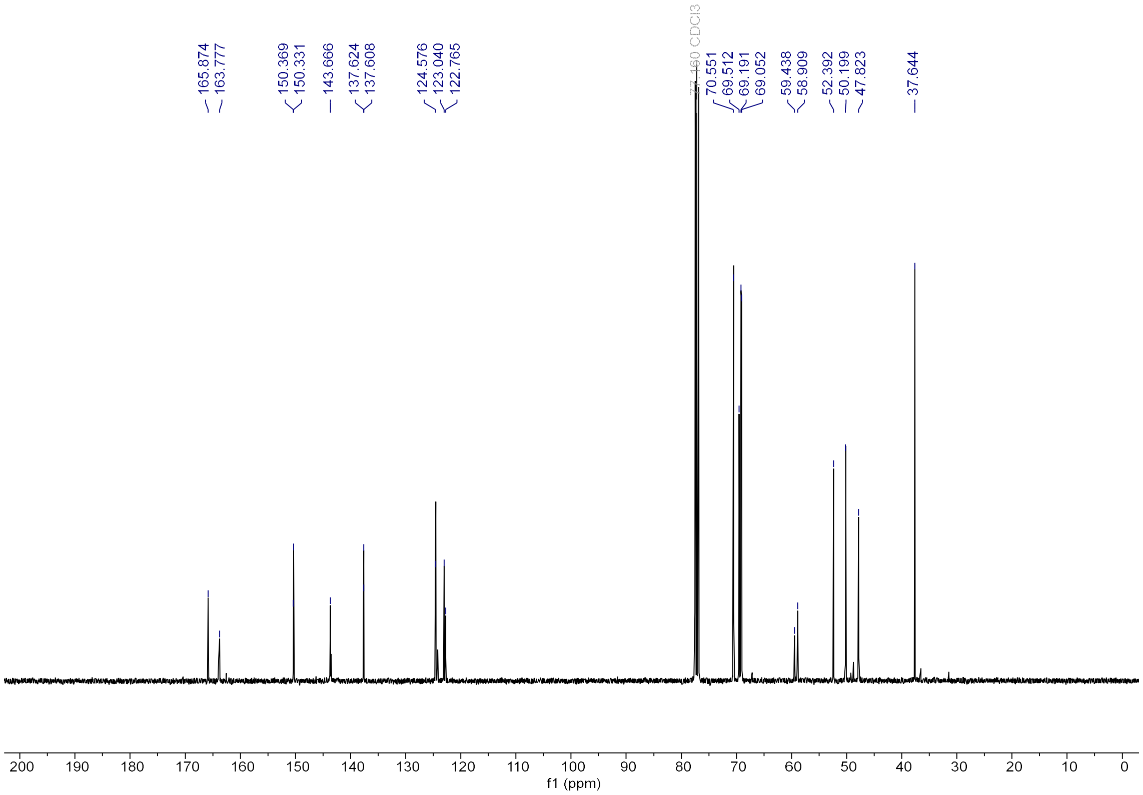

6.15 Compound S21

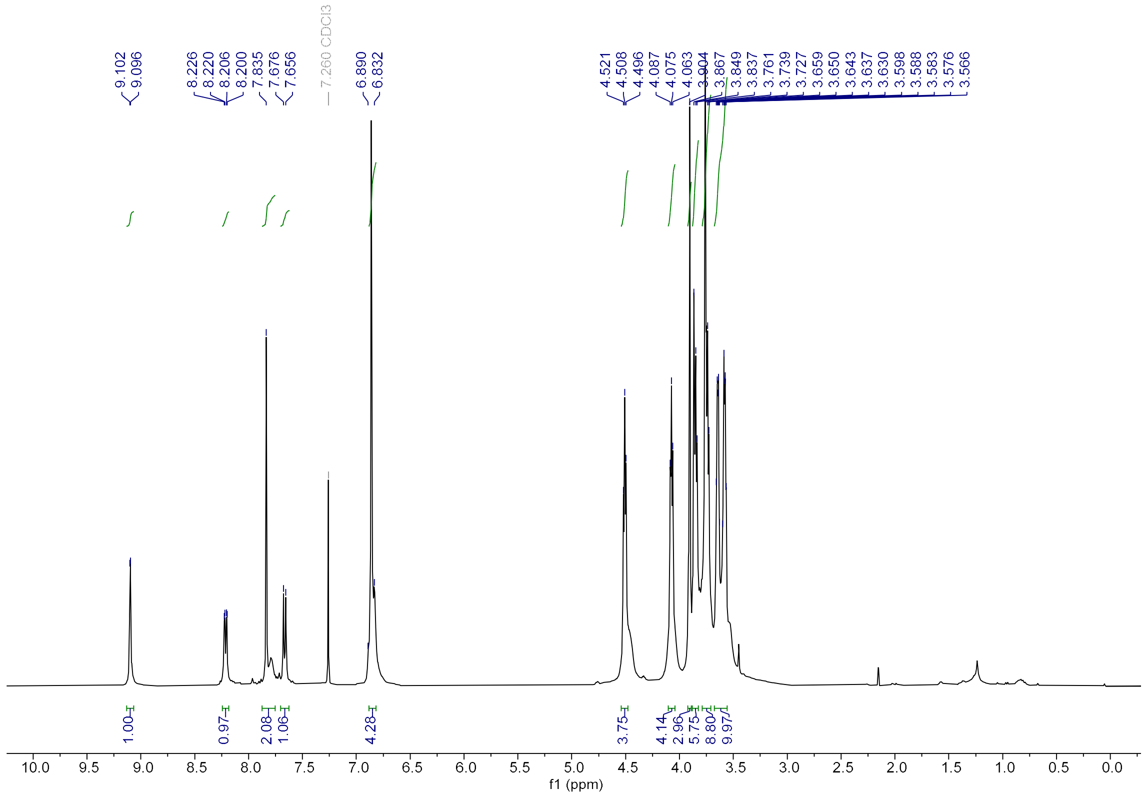

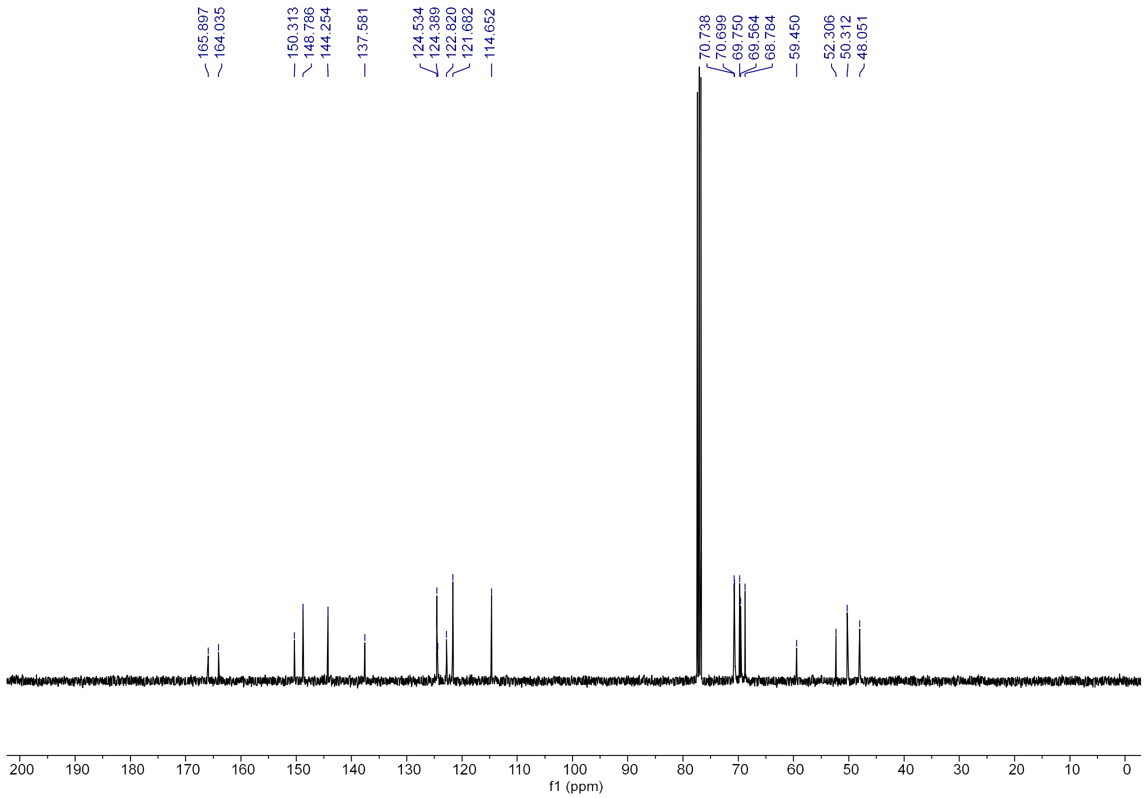

7. References

S1. G. R. Fulmer, A. J. M. Miller, N. H. Sherden, H. E. Gottlieb, A. Nudelman, B. M. Stoltz, J. E. Bercaw, K. I. Goldberg, *Organometallics*, 2010, **29**, 2176–2179.

S2. C. P. Rosenau, B. J. Jelier, A. D. Gossert, A. Togni, *Angew. Chem., Int. Ed.*, 2018, **57**, 9528–9533.

S3. H. Struthers, B. Spingler, T. L. Mindt, R. Schibli, *Chem. Eur. J.*, 2008, **14**, 6173–6183.

S4. A. D. Peters, S. Borsley, F. della Sala, D. F. Cairns-Gibson, M. Leonidou, J. Clayden, G. F. S. Whitehead, I. J. Vitórica-Yrezábal, E. Takano, J. Burthem, S. L. Cockroft, S. J. Webb, *Chem. Sci.*, 2020, **11**, 7023–7030.

S5. S. Wang, F. della Sala, M. J. Cliff, G. F. S. Whitehead, I. J. Vitórica-Yrezábal, S. J. Webb, *J. Am. Chem. Soc.*, 2022, **144**, 21648–21657.

S6. N. Eccles, B. A. F. Le Bailly, F. della Sala, I. J. Vitórica-Yrezábal, J. Clayden, S. J. Webb, *Chem. Commun.*, 2019, **55**, 9331–9334.

S7. P. Kuzmic, *Anal. Biochem.*, 1996, **237**, 260–273.

S8. C. Hübler, *Chem. Methods*, 2022, **2**, e202200006.

S9. G. Kleks, D. C. Holland, J. Porter, A. R. Carroll, *Chem. Sci.*, 2021, **12**, 10930.

S10. *Automatic DOSY Processing*, https://resources.mestrelab.com/dosy/ (accessed September 2023).

S11. G. R. Fulmer, A. J. M. Miller, N. H. Sherden, H. E. Gottlieb, A. Nudelman, B. M. Stoltz, J. E. Bercaw, K. I. Goldberg, *Organometallics*, 2010, **29**, 2176–2179.

S12. For clarity, downfield shifts of resonances ($\Delta\delta$) in NMR spectroscopy are positive while upfield shifts are negative.

S13. M. De Poli, L. Byrne, R. A. Brown, J. Solà, A. Castellanos, T. Boddaert, R. Wechsel, J. D. Beadle, J. Clayden, *J. Org. Chem.*, 2014, **79**, 4659–4675.

S14. B. A. F. Le Bailly, L. Byrne, V. Diemer, M. Foroozandeh, G. A. Morris, J. Clayden, *Chem. Sci.*, 2015, **6**, 2313–2322.
